# DR-GEM enables self-supervised machine learning for single-cell embeddings and annotations

**DOI:** 10.1038/s41467-026-77908-z

**Published:** 2026-09-24

**Authors:** Christine Yiwen Yeh, Min Woo Sun, Dixian Zhu, Livnat Jerby

**Affiliations:** 1 Department of Biomedical Data Science, Stanford University School of Medicine, Stanford, CA USA; 2 Department of Genetics, Stanford University School of Medicine, Stanford, CA USA; 3 Department of Medicine, Stanford University School of Medicine, Stanford, CA USA; 4 Stanford Cancer Institute, Stanford University School of Medicine, Stanford, CA USA; 5 Chan Zuckerberg Biohub, San Francisco, CA USA

**Keywords:** Data processing, Transcriptomics, Machine learning

## Abstract

Dimensionality reduction and clustering are instrumental to single-cell and spatial genomics data analysis. Here we show that existing methods tend to fit oversampled classes and miss more unique signals and patterns, such as rare cell types and states. We find that the problem in such cases is not primarily data scarcity per se, but the model equating abundance with importance. Addressing this, we present “Distributionally Robust and latent Group-AwarE consensus Machine learning” or DR-GEM, a self-supervised meta-algorithm that uses the reconstruction error to reorient the model attention to patterns it initially missed and applies balanced consensus learning to increase robustness and filter low-quality data. We apply DR-GEM to synthetic and real-world single-cell, spatial transcriptomics, and Perturb-seq datasets and demonstrate that it outperforms existing methods in obtaining reliable embeddings, recovering rare cell types, filtering noise, mitigating cell-level and patient-level underrepresentation, and uncovering held-out genetic and spatial features. We thus surface and address an underappreciated problem and present a self-supervised framework to help support AI-based and data-driven discoveries.

## Abstract (web summary)

Here the authors show that current dimensionality reduction methods for single-cell and spatial genomics data often favor abundant patterns and overlook rare ones. They develop DR-GEM, a self-supervised framework that diagnoses and learns from its errors to recover underrepresented biological signals.

## Introduction

Biomedical research increasingly relies on unsupervised representation learning to transform high-dimensional biological measurements into interpretable maps of cells, tissues, perturbations, and molecules. Dimensionality reduction and clustering define the geometry on which cell types are annotated^1–3^, disease subtypes are discovered^4–8^, perturbation effects are compared^9–11^, and biological hypotheses are generated^12–14^. Lower-dimensional embeddings often have higher signal-to-noise ratios compared to the original measured features and serve as the starting point for downstream clustering, labeling, differential analyses, and trajectory inference. Systematic distortions in the embeddings can thus propagate through an entire analysis workflow.

A pervasive but under-addressed source of such distortion is latent class imbalance. Biomedical datasets rarely sample classes evenly and are often dominated by common or easy-to-access cell types, disease states, ancestries, perturbation responses, or protein families. Yet underrepresented classes or rare patterns can be of substantial biological and clinical importance. For example, stem cells and progenitor populations constitute a small subset of the tissue but are critical for tissue homeostasis and regeneration^15–17^, minimal-residual disease^18^ and drug-tolerant^19^ cancer cells can be small subpopulations within the entire tumor but be the main drivers of relapse; and, in the context of large screens, a genetic perturbation may lead to a unique cell state and hence be manifested only in a small fraction of the cells in the data^10,14^.

Tools for countering class imbalance and the resulting suboptimal model performance^20^ originated largely in supervised learning and include reweighting and oversampling^21–23^, fairness-aware training^24,25^, group-distributionally robust optimization^26^, and worst-case or tail objectives such as conditional value-at-risk (CVaR)^27^ and divergence-constrained DRO^28–30^. A subset of these principles has been carried into unsupervised representation learning, for example, “fair” variants of principal component analysis (PCA) and multi-criteria dimensionality reduction that seek embeddings that equalize reconstruction error (RE) across groups^31–35^, with analogous formulations for clustering^36,37^. These methods establish that balanced objectives can be imposed on unsupervised representations, but, as they require class assignment as input, are not designed to improve de novo latent class discovery itself.

Most prevalent approaches to counter imbalance in genomics have been to extend the data collection via improved throughput^9,38–42^ and large consortiums^1,43–45^, developing tailored methods to detect rare entities^46–48^, and merging diverse datasets through unified pipelines^12,13,43,49^, reference maps^50–52^, and foundation models^53,54^. These are critical efforts, but do not directly resolve the objective-level bias of representation learning methods, which typically minimize losses averaged over data points rather than over distinct entities and patterns. The latter has the potential to be more robust to fluctuations in the data composition and, by not equating abundance with importance, better capture the underlying geometry of the data^31,32,34,55^, and better support scientific discovery.

Here we present DR-GEM (Distributionally Robust and latent Group-AwarE consensus Machine learning), a self-supervised meta-algorithm for distributionally robust dimensionality reduction and clustering. DR-GEM uses the RE as a self-supervised signal to identify and reorient the model's attention to areas where it is underperforming, and consensus learning to parse signal from noise. The results demonstrate that latent class imbalance distorts widely used workflows and is not adequately addressed by existing methods. DR-GEM outperforms existing unsupervised methods in reconstructing latent transcriptional structure in the context of latent-class imbalance and technical noise, detecting rare cell populations, and capturing held-out genetic and spatial features, as we show using synthetic and real-world single-cell^56–59^, spatial^14,60^, and perturbational^10^ datasets across a variety of platforms and biological settings.

## Results

### Minority/rare cells are poorly embedded and prone to mislabeling

**Fig. 1:**
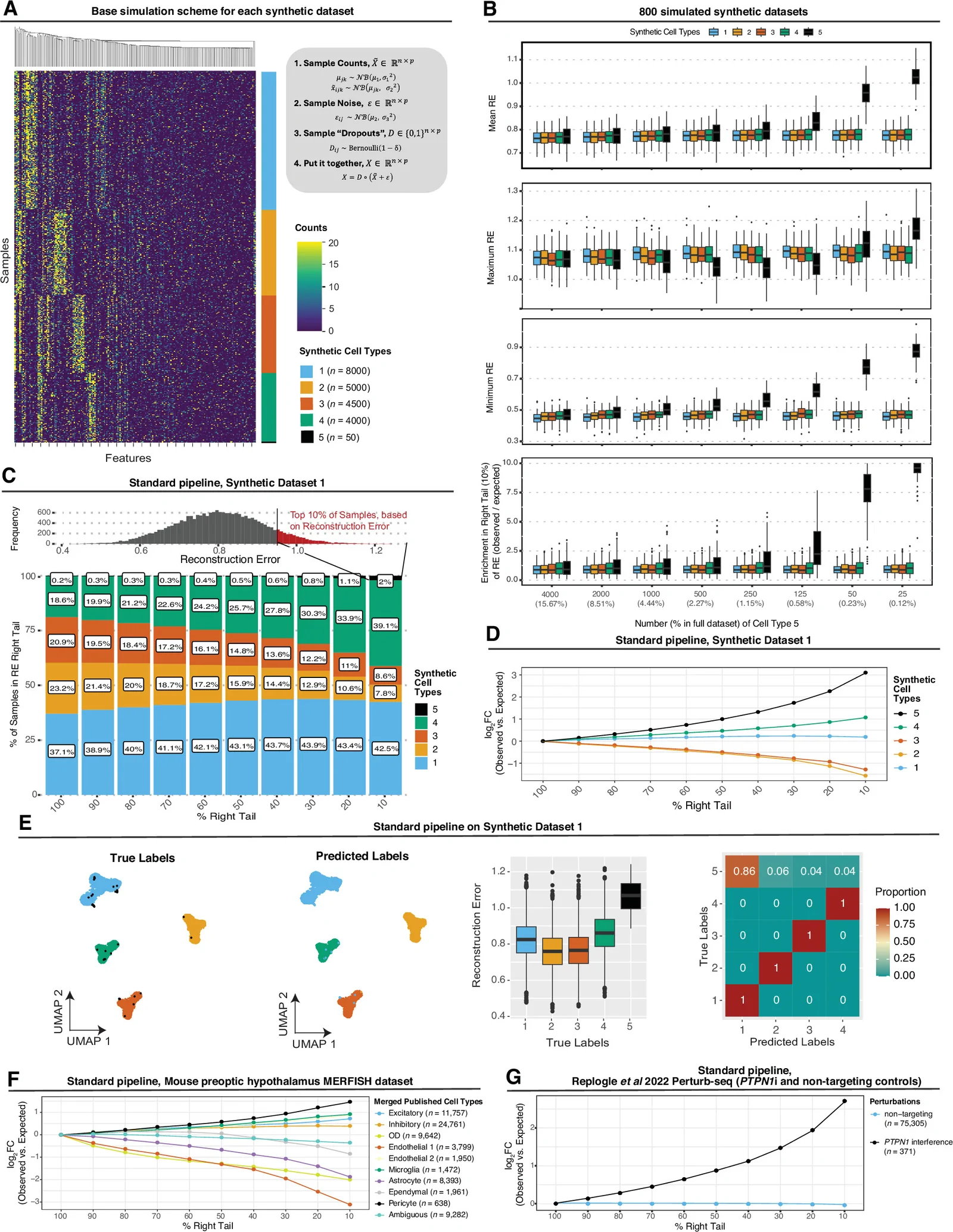
Minority/rare cells are poorly embedded and prone to mislabeling with the standard single cell analysis pipeline. **A** Heatmap of Synthetic Dataset 1: values are drawn from two-level negative binomial distributions to model gene expression of 5 synthetic cell types (**Methods**). Data generation is described for cell *i*, gene *j*, and cell type *k*. **B** From top to bottom: Mean RE, maximum RE, minimum RE, and FC enrichment in the top 10% tail of RE distribution of each cell type (fill color) in 800 simulated datasets with decreasing rare cell type frequency (*x*-axis; *n* = 100 datasets per frequency). **C** Top: histogram of RE in Synthetic Dataset 1. Bottom: Cell type percentage (*y*-axis) in the RE right-tail, shown across varying right-tail sizes (*x*-axis). **D** Observed vs. expected cell type proportion (log_2_FC; *y*-axis) in the RE right-tail (*x*-axis) of Synthetic Dataset 1. **E** Performance of the standard pipeline on Synthetic Dataset 1: UMAP visualization of samples colored by ground truth synthetic cell type labels (left) and predicted cluster labels (middle left), boxplots of RE (*y*-axis) in each ground truth synthetic cell type (*x*-axis, middle right), and proportion of predicted cell type (*x*-axis) in each of the true cell type populations (*y*-axis; right). Label colors are the same as in (**A**–**C**), depicting Synthetic Dataset 1 (*n* = 21,550 cells, with cell types labeled as 1-5 from most to least abundant: *n* = 8000, 5000, 4500, 4000, and 50). Observed vs. expected cell type proportion (log_2_FC; *y*-axis) in the RE right-tail of a mouse preoptic hypothalamus MERFISH dataset^60^ (**F**) and a Perturb-seq dataset^10^ with NTC and *PTPN1* interference (*PTPN1*i) cells (**G**), shown across varying right-tail sizes (*x*-axis). **B** and **E** Boxplots middle line: median; box edges: 25th and 75th percentiles; whiskers: most extreme points that do not exceed ± interquartile range (IQR) x 1.5; further outliers are marked individually as black points. NB negative binomial, FC fold change (observed versus expected), MERFISH multiplexed error-robust fluorescence in situ hybridization, NTC non-targeting control, PTPN1i CRISPR interference (CRISPRi) targeting *PTPN1*, *RE* reconstruction error; *OD* oligodendrocyte. Source data are provided as a Source Data file.

Single-cell analysis pipelines often string together several unsupervised algorithms: (1) dimensionality reduction, (2) clustering, (3) embedding for visualization, and (4) cluster-level annotations to cell (sub)types or states. Commonly used methods for steps 1-3 are PCA, the Louvain algorithm^61^ applied to a Shared Nearest Neighbors (SNN) graph^62^, and Uniform Manifold Approximation and Projection (UMAP), respectively, which we refer to as the standard pipeline. To empirically test and demonstrate the challenges posed by latent class imbalance in a controlled manner, we start by testing this common standard pipeline on simulated synthetic single-cell ‘omics data featuring latent class imbalance with ground truth labels (Fig. 1A). The synthetic data consists of simulated gene expression counts sampled from negative binomial distributions. Each cell is assigned to one of *K* cell types and is simulated to overexpress its cell type marker genes (Fig. 1A, **Methods**). An additional smaller set of features is added to model a secondary gene module with cell-type-invariant variation, followed by the addition of negative binomial noise ε and randomly setting a fraction of the values to 0 to simulate dropouts (Fig. 1A**, Methods**).

Using this scheme, we simulated 100 unique synthetic single-cell ‘omics datasets for each of 8 different levels of frequency of one rare cell type (Synthetic Cell Type 5) and fit PCA on all 800 simulated datasets. Based on per-cell RE, that is, the distance between the input data and the data reconstructed from the Principal Component (PC) embedding, and the ground-truth cell type annotations, cells representing the rare cell type are prone to have significantly poorer embeddings as a function of the degree of underrepresentation (Fig. 1B–D). Subsequent Louvain-SNN clustering on the latent lower-dimensional space yielded incorrect cluster assignments for the rare cells in a representative baseline synthetic dataset (Synthetic Dataset 1, Fig. 1E; *p* = 3.96 × 10^−^^20^, *W* = 1035954, *r*_rb_ = 0.93, 95% Confidence Interval (CI) [0.90, 0.95], RE of Synthetic Cell Type 5 vs. Synthetic Cell Type 1-4, one-sided Wilcoxon rank-sum test). The rare cells do not form a cluster of their own and instead are misassigned to other clusters (Fig. 1E).

Analytically, we show that the covariance of rare cell type marker genes scales with cell type frequency, further explaining why rare cells are systematically prone to high RE in the low-dimensional PC space (**Methods**). More specifically, the covariance of cell type marker genes can be represented as a weighted average of (1) their strength as markers and (2) their covariance within the cell type they represent, such that 50% cell type frequency is the tipping point when the latter becomes more dominant. Under these circumstances, PCA sensitivity in detecting the abundant cell type can also be impaired, especially if the marker genes are not strongly correlated in their expression within the cell type they represent. This is observed in the synthetic data, where although all cell types were simulated in the same manner, the variance of genes that mark the rare cell types is lower across the full dataset (Supplementary Fig. 1).

To examine this in real-world data, we compiled eight single-cell datasets spanning diverse biological contexts and data types, including scRNA-seq (profiling head and neck squamous cell carcinoma^58^, peripheral blood mononuclear cells (PBMC)^63^, human thymus development^56^, tracheal epithelium of control and mouse models of cystic fibrosis^57^), spatial transcriptomics (HGSC^14^, mouse preoptic hypothalamus^60^), imaging mass cytometry proteomics (breast cancer^59^), and Perturb-seq data^10^.

All datasets included rare cell types, rare cell states, or rare genetic perturbations. For example, in the developing thymus, rare transient progenitor and differentiation states were identified in the original study via integrated developmental trajectories, conserved external gene signatures, and independent validation across donors and held-out datasets^56^. In the tracheal epithelium of control and mouse models of cystic fibrosis dataset, rare epithelial subtypes were identified in the original study via integrated analyses with complementary bulk sequencing, histology, and mouse genotype^57^. In the Perturb-seq dataset, there are underrepresented perturbations, resulting either from uneven single-guide RNA (sgRNA) distribution in the initial library or from sgRNA fitness effects. In all cases, cells of the rare cell type, rare state, or rare genetic perturbation are 1.15–9.18 times more likely to be among the most poorly embedded cells (cells with the top 10th percentile of RE values, Fig. 1F, G, Supplementary Fig. 2, **Methods**). The estimated variance of cell type marker genes was correlated with the cell type abundance also in the real-world datasets, as shown for two spatial transcriptomics datasets (Supplementary Fig. 3).

### Existing DRO and fairness-aware algorithms are insufficient to improve rare cell embedding and labeling

As is true with most machine learning algorithms, the objective of dimensionality reduction is often to (either explicitly or implicitly) minimize an average error. Consequently, common patterns in the data dominate the model fitting process. Rare samples contribute less to the overall average error, and hence models are inherently biased to underperform on them^31,32,34,55^. These types of limitations have led to the development of DRO – a family of optimization approaches that shifts the focus from minimizing average loss to minimizing worst-case loss and considers a wider range of data distributions for robustness.

**Fig. 2:**
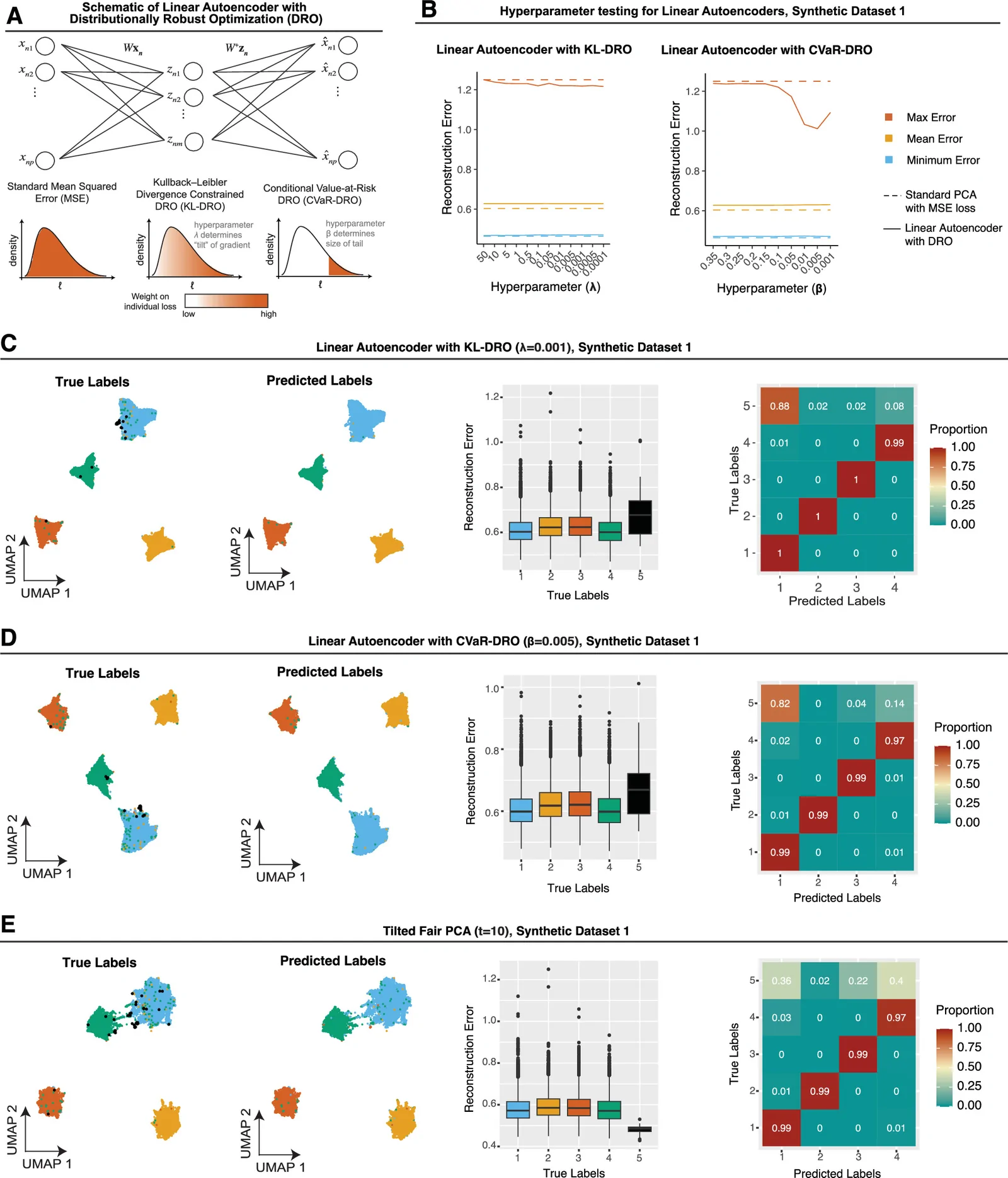
Existing DRO and fairness-aware algorithms performances in rare cell embedding and detection. **A** Schematic of PCA optimized under the KL-DRO^64^ and CVaR-DRO^27^ objectives (**Methods**). **B** Maximum (red), mean (yellow), and minimum (blue) RE (*y*-axis) of the dataset as a function of the relevant hyperparameter (*x*-axis) in PCA solved via KL-DRO (solid line, left) or CVaR-DRO (solid line, right) vs. default PCA (dotted line). Pipeline performance with PCA solved via KL-DRO (**C**), CVaR-DRO (**D**), and Tilted Fair PCA^34,65^ (**E**) on Synthetic Dataset 1. UMAP visualization of cells colored by ground truth cell type labels (left) and predicted cluster labels (middle left), colors the same as in Fig. 1A; boxplots of RE (*y*-axis) in each ground truth cell type population (*x*-axis; middle right); proportions of the predicted cell types (*x*-axis) in each true cell type population (*y*-axis; right). **C**–**E** Each boxplot depicts *n* = 21,550 cells in Synthetic Dataset 1; cell types are labeled as 1–5 from most to least abundant: *n* = 8000, 5000, 4500, 4000, and 50; boxplots middle line: median; box edges: 25th and 75th percentiles; whiskers: most extreme points that do not exceed ± IQR x 1.5; further outliers are marked individually as black points. CVaR conditional value-at-risk, DRO distributionally robust optimization, KL Kullback–Leibler divergence, MSE mean squared error, RE reconstruction error. Source data are provided as a Source Data file.

We thus turned to test DRO dimensionality reduction algorithms to examine their performance in the context of latent class imbalance and rare cell type identification (Fig. 2A). Applied to Synthetic Dataset 1, Kullback-Leibler Divergence Constrained DRO (KL-DRO^64^) and CVaR-DRO^27^ successfully achieved the DRO objectives to better minimize worst-case RE across multiple hyperparameters (Fig. 2B), but did not sufficiently improve the embedding and assignment of rare cells compared to the standard pipeline (Fig. 2C-D). The rare cells (Synthetic Cell Type 5) continue to have a significantly higher RE compared to all other cell types (KL-DRO (λ = 0.001): *p* = 2.52 × 10^−6^, *W* = 737,997, *r*_rb_ = 0.37, 95% CI [0.23, 0.50]; CVaR-DRO (β = 0.005): *p* = 8.98 × 10^−^^6^, *W* = 725,937, *r*_*rb*_ = 0.35, 95% CI [0.20, 0.48], one-sided Wilcoxon rank-sum test), do not cluster apart from the other cells, and are mislabeled (Fig. 2C, D). The lack of performance improvement with DRO may be explained by the fact that the optimization schemes do not explicitly minimize a single group’s error.

Therefore, we turned to a different conceptual approach of algorithmic fairness wherein the optimization utilizes class labels explicitly to promote fairness. However, even when providing the ground truth latent class information as input, applying Tilted Fair PCA^34,35,65^ to Synthetic Dataset 1 with an optimized hyper-parameter (*t* = 10, **Methods**) decreased the RE of the rare cell type (Fig. 2E, *p* = 1, *W* = 28466, *r*_rb_ = −0.95, 95% CI [−0.96, −0.93], RE of Synthetic Cell Type 5 vs. all other cells, one-sided Wilcoxon rank-sum test), but nonetheless did not cluster the rare cells apart from the other cells and mislabeled them (Fig. 2E).

These investigations of DRO (KL-DRO and CVaR-DRO) and algorithmic fairness (Tilted Fair PCA) suggest that existing methods and concepts for mitigating bias in dimensionality reduction are insufficient to obtain reliable low-dimensional embeddings and accurate labeling for imbalanced datasets. This prompted us to develop an end-to-end method to mitigate these problems, with a focus on single-cell data analysis.

### DR-GEM for distributionally robust dimensionality reduction and clustering

To address the limitations of existing methods, we developed DR-GEM – an iterative self-supervised framework for dimensionality reduction and clustering of datasets with latent class imbalance. DR-GEM has three main phases: (Phase I) uses the RE as a self-supervised signal to identify and reorient the model’s attention to areas where it is underperforming; (Phase II) applies consensus learning to parse signal from noise; and (Phase III) obtains a reference map and cell annotations.

**Fig. 3:**
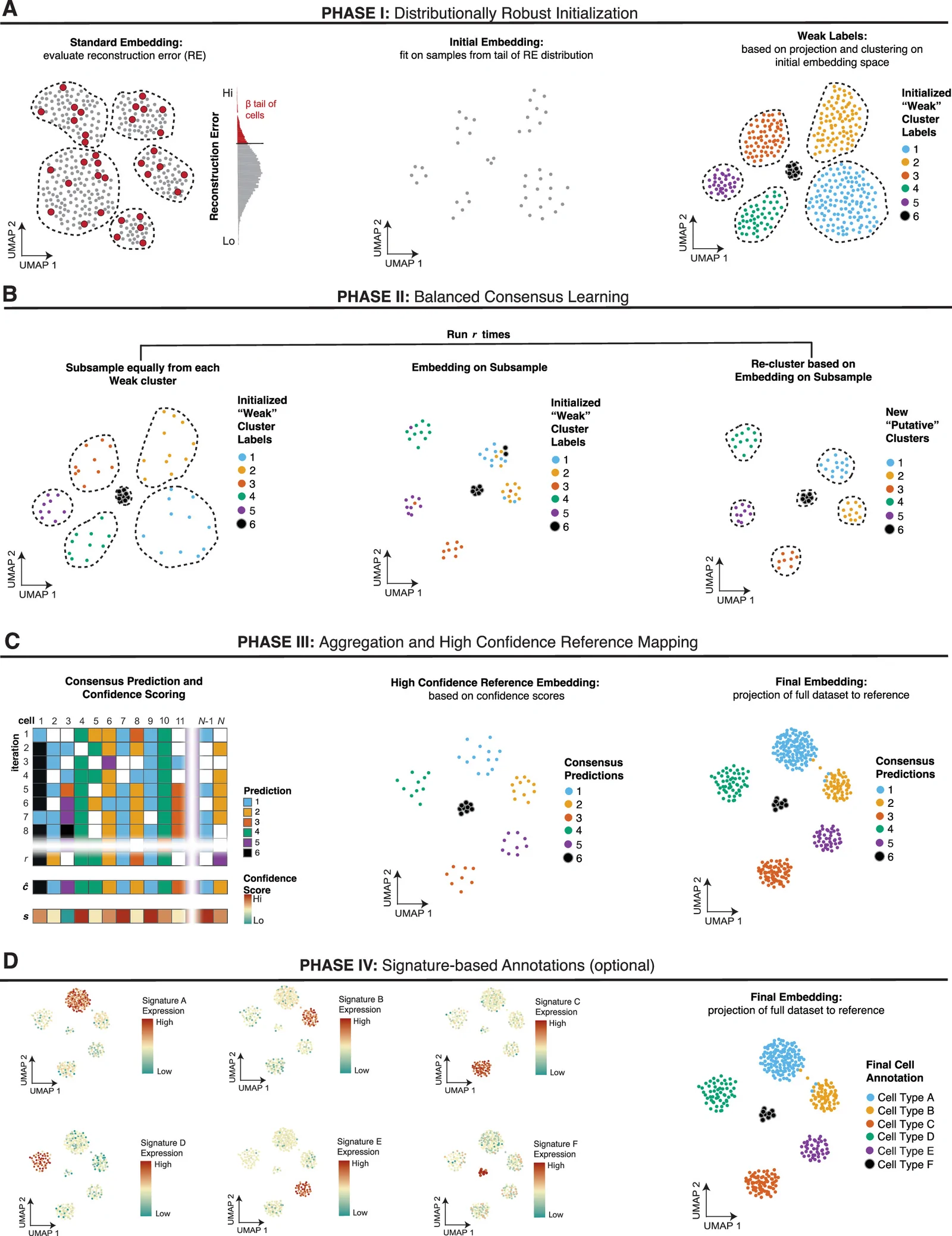
DR-GEM is a self-supervised meta-algorithm for dimensionality reduction and clustering. **A** Phase I: Standard dimensionality reduction is performed on the entire dataset to compute RE per cell (left); cells in the *β* right tail of the RE distribution are used to generate an initial embedding (*E*^(init)^, middle); all cells in the dataset are projected to *E*^(init)^ and clustered to obtain weak clustering labels (***c***^(1)^). **B** Phase II: *r* unique, random, but balanced (across ***c***^(1)^ weak cluster labels) subsamples of the dataset are separately embedded and clustered to generate *r* sets of latent class predictions. **C** Phase III: Aggregation of ensemble predictions across iterations from (B) yields consensus cluster labels ($\hat{{{{\boldsymbol{c}}}}}$) and sample label confidence (***s***) (left); a balanced subsample of high confidence cells based on score***s s*** is used to generate a new high confidence reference embedding (middle) to which all cells are projected for visualization (right). **D** DR-GEM includes optional functionality to utilize the expression of gene signatures in each cluster (left) to automatically assign clusters to known cell type/states (right). See **Methods** for more detail. Abbreviation: RE, reconstruction error.

In Phase I (Distributional Robust Initialization, Fig. 3A), DR-GEM first fits a standard embedding (*E*^(0)^) using classic dimensionality reduction (e.g., PCA) and computes the RE per cell (Fig. 3A, left). Under the key observation that rare latent populations tend to be enriched in the right tail of the RE distribution, DR-GEM fits a new embedding *E*^(init)^ on cells with the highest RE values from *E*^(0)^ (Fig. 3A, middle), thereby shifting the dimensionality reduction algorithm’s focus to these putative high-risk cells. DR-GEM then projects all remaining cells to the *E*^(init)^ subspace and uses this embedding to identify initial clusters ***c***^(1)^ (Fig. 3A, right).

In Phase II (Balanced Consensus Learning; Fig. 3B), DR-GEM applies consensus learning, a technique in ensemble machine learning where an array of models is fit, each based on a subset of the data, and the results can then be aggregated to increase accuracy and robustness. To promote latent class balance, DR-GEM randomly samples uniformly from the initial clusters ***c***^(1)^ (Fig. 3B, **Methods**), effectively upweighting rare cells across each of the *r* unique subsamples. DR-GEM fits a lower-dimensional embedding and generates cluster annotations for each of the *r* unique subsamples. This yields an ensemble of cluster predictions for each cell. Cluster annotations in each run are aligned via the Kuhn-Munkres^66,67^ algorithm to consolidate the annotations (**Methods**). Of note, DR-GEM has built-in mechanisms to track the convergence of the ensemble predictions for an automated selection of the *r* hyperparameter, as well as a modular design to add additional iterations to Phase II in a cost-effective manner (**Methods**).

In Phase III (Aggregation and High Confidence Reference Mapping; Fig. 3C), DR-GEM aggregates the solutions by assigning each cell to the cluster it is most frequently assigned to in Phase II. A confidence score is computed per cell to denote the fraction of runs where the cell was assigned to its final cluster. Lastly, DR-GEM uniformly samples high-confidence cells across the final clusters to generate a final reference embedding to which all the cells are projected for visualization and clustering. High-confidence cells are defined as cells with *s* > *t*, where *t* is a user-defined parameter. As described in subsequent sections, empirical testing shows that high-confidence cells are less likely to be contaminated with ambient RNA or be doublets, thus generating an embedding that is less impacted by these technical confounders. However, in developing tissues or other contexts where cell state transitions are expected, low confidence scores may capture biologically meaningful transitions and are recommended to be further characterized.

In an optional Phase IV (Signature-based Annotation; Fig. 3D), DR-GEM uses predefined gene signatures of cell types or cell states to assign clusters to known cell types or cell states and support end-to-end single-cell annotations, as well as intermediate cell type/state predictions (**Methods**).

As output, DR-GEM returns the reference map, the initial embedding of all cells and their projection to the reference map, along with the consensus clustering labels and confidence scores.

DR-GEM can be used with any dimensionality reduction or clustering algorithm as input. Unless specified, we applied DR-GEM with PCA and Louvain-SNN clustering, a standard pair of algorithms used in single-cell analyses.

### DR-GEM recovers latent patterns and labels in synthetic datasets with latent class imbalance

**Fig. 4:**
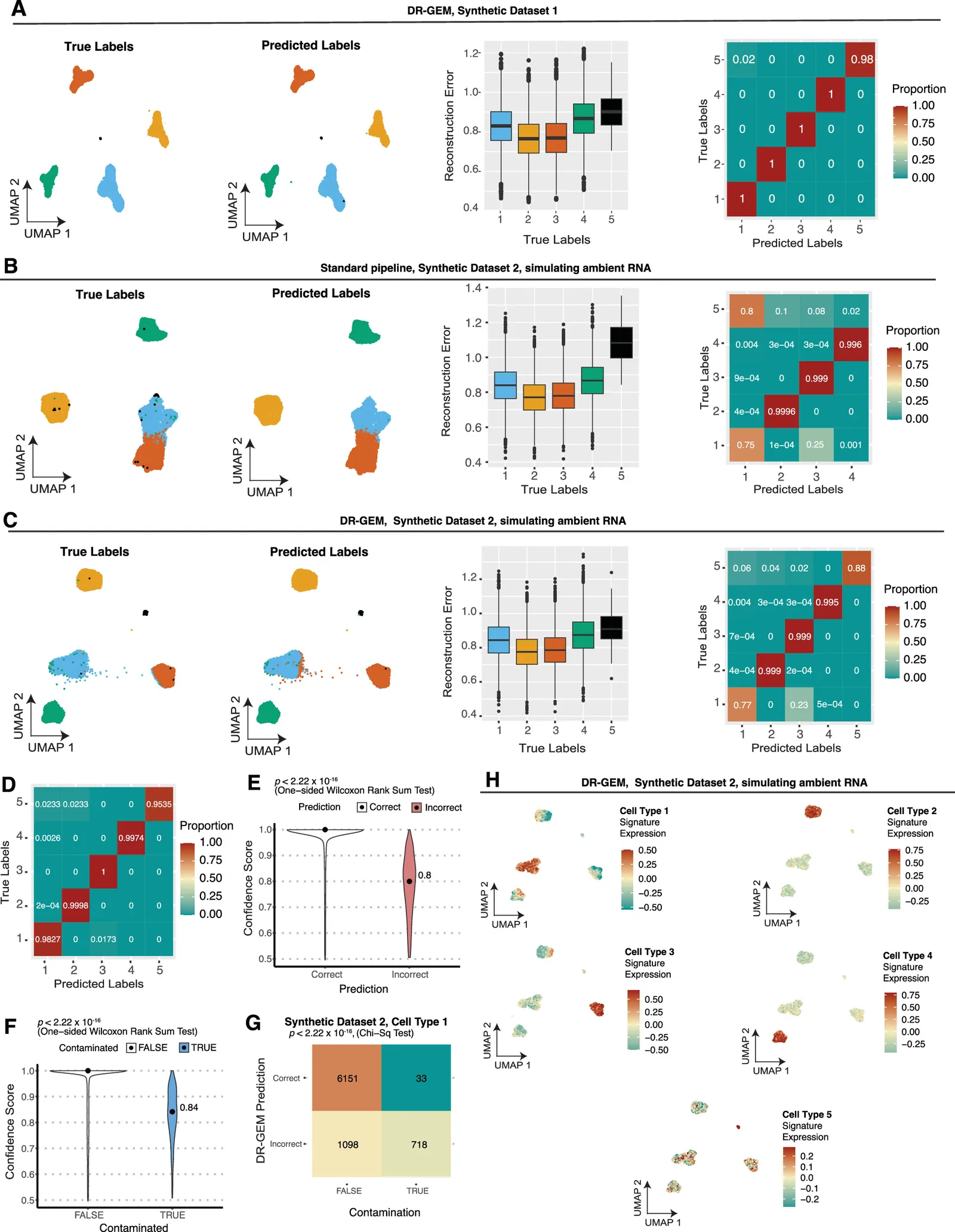
DR-GEM outperforms alternative methods and improves the embedding and labeling of rare cells in synthetic datasets. Performance of (**A**) DR-GEM on Synthetic Dataset 1 (**B**) standard pipeline on Synthetic Dataset 2 (with ambient RNA), and (**C**) DR-GEM on Synthetic Dataset 2. **A**–**C** UMAP visualization of cells colored by ground truth synthetic cell type labels (left) and predicted cluster labels (middle left), boxplots of RE (*y*-axis) in each ground truth cell type population (*x*-axis, middle right), and proportion of predicted cell type (*x*-axis) in each of the true cell type populations (*y*-axis; right). Label colors the same as in Fig. 1A. **D**–**H** Additional performance metrics for DR-GEM applied to Synthetic Dataset 2. **D** Proportion of DR-GEM predicted cell type (*x*-axis) in each of the true cell type populations (*y*-axis; right) in confidence-filtered (*s*_*i*_ > 0.95) Synthetic Dataset 2. Violin plots of DR-GEM confidence scores (*y*-axis) as a function of **E** whether the prediction was correct or not, and **F** whether a cell was contaminated or not (*x*-axis). **G** The number of Cell Type 1 cells DR-GEM predicted correctly/incorrectly (*y*-axis) stratified by contamination status (*x*-axis). (H) UMAP visualization of high-confidence cells in the high-confidence reference embedding from DR-GEM. Cells colored by overall expression of multi-feature signature of simulated Cell Types 1-5 (Methods). (A-C) *n* = 21,550 cells labeled as Cell Type 1-5 from most to least abundant: *n* = 8000, 5000, 4500, 4000, and 50. Boxplots middle line: median; box edges: 25th and 75th percentiles; whiskers: most extreme points that do not exceed ± IQR x 1.5; further outliers are marked individually as black points. **E**, **F** Violin plots: median denoted with a dot labeled with the median value.

Applying DR-GEM to Synthetic Dataset 1 shows that, unlike other pipelines (PCA, KL-DRO, CVaR-DRO, and Fair PCA; Figs. 1E, 2C–E), only with DR-GEM the rare cells form their own cluster and are correctly annotated (98%, Fig. 4A). DR-GEM balances out the RE between the cell groups (*p* = 0.78, *W* = 53270489, *r*_rb_ = -0.02, 95% CI [-0.03, 0.00], RE of Synthetic Cell Type 5 vs. all other cells, one-sided Wilcoxon rank-sum test), amplifies the gene expression variance of marker genes of Synthetic Cell Type 5 (Supplementary Data 1), and improves the prediction of the ground truth cell labels across all balanced clustering prediction performance^68^ metrics (Fig. 4A, Supplementary Data 2). As KL-DRO, CVaR-DRO, and Fair PCA did not outperform the standard pipeline, we next focused on benchmarking DR-GEM against the standard pipeline with an array of synthetic and real-world datasets.

Going beyond PCA, we test DR-GEM compared to the standard pipeline, with both applied with: (i) Kernel PCA with a polynomial (degree = 2) kernel (Supplementary Fig. 4), (ii) Latent Dirichlet Allocation^69^ (LDA, Supplementary Fig. 5A, B), and (iii) the Variational Autoencoder (VAE)-based method, scVI^70^ (Supplementary Fig. 5C, D). As with PCA, each of these methods showed that the rare cell type had elevated RE values in the embeddings obtained with the standard pipeline, such that DR-GEM improved the embedding and detection of the rare cell type (Supplementary Figs. 4, 5).

Methods such as Tilted Fair PCA^65^, class-weighted PCA^71^, and Synthetic Minority Oversampling Technique (SMOTE)^21^ are not comparable to DR-GEM as they require prior labels. However, these can be integrated with DR-GEM, such that labels from DR-GEM phase I are used to identify latent classes and rare cells provided as input for these methods. In accordance with the performances of Tilted Fair PCA (Fig. 2E), DR-GEM variant with class-weighted PCA reduced the RE discrepancy between the rare cells and the majority cell types but nonetheless did not cluster the rare cells apart from the other cells, resulting in mislabeling (Supplementary Fig. 6A). DR-GEM with SMOTE reduced the RE discrepancy and correctly clustered and annotated all cell types, including the rare cells (100% accuracy), but distorted the rare cell population expression landscape, falsely representing the rare cell type population as highly heterogeneous compared to the other cell types, raising questions concerning the validity and reliability of the SMOTE augmentation (Supplementary Fig. 6B, C).

### DR-GEM recovers signal from noisy data

While important biological signals can be rare or nuanced, technical noise can be pervasive but often unevenly distributed. For example, in scRNA-seq, mRNA molecules that have been released in the cell suspension or during tissue dissociation can contaminate the microfluidic droplets and get falsely assigned to cells. Microfluidic droplets can sometimes include more than one cell, thereby harboring signals that are a mixture of two or more cells^72–74^. In spatial transcriptomics, RNA molecules from one cell can be falsely assigned to a nearby cell due to lateral RNA diffusion and limitations of image segmentation^14,75–77^.

To test DR-GEM in such settings, we generated Synthetic Dataset 2, which features both latent class imbalance and ambient RNA (**Methods**, Supplementary Data 3). DR-GEM outperformed the standard pipeline in Synthetic Dataset 2 (Fig. 4B–H), as it: (1) equalized RE across the synthetic cell types (*p* = 1.40 × 10^-27^, *W* = 1,034,954, *r*_rb_ = 0.93, 95% CI [0.90, 0.95], and *p* = 0.75, *W* = 29,813,725, *r*_rb_ = 0.28, 95% CI [0.26, 0.30], RE of rare vs. all other cell types, one-sided Wilcoxon rank-sum test in the standard and DR-GEM pipeline, respectively; Figs. 4B, C), (2) outperformed on all balanced clustering metrics^68^ (**Methods**, Supplementary Data 2), and (3) clustered the rare cells as a separate cluster, such that 88% of the rare cells were correctly annotated (Fig. 4C), compared to 0% with the standard pipeline (Fig. 4B). DR-GEM balanced subsampling also amplified the gene expression variance of marker genes of Synthetic Cell Type 5 in Synthetic Dataset 2 (Supplementary Data 1).

DR-GEM assigned 86.9% and 99.0% of the cells with ambient RNA to their correct label when considering all contaminated cells or only those with high confidence scores (*s* > 0.95), respectively (Fig. 4D–H), while the standard pipeline assigned 84.3% of the cells with ambient RNA to their correct label. Considering only the contaminated rare cells, DR-GEM and the standard pipeline assigned 64.7% and 0% of these cells to the correct label, respectively. The confidence scores correctly identified poorly annotated cells and were significantly lower for cells with incorrect assignments (Fig. 4E) and contaminated cells (Fig. 4F).

**Fig. 5:**
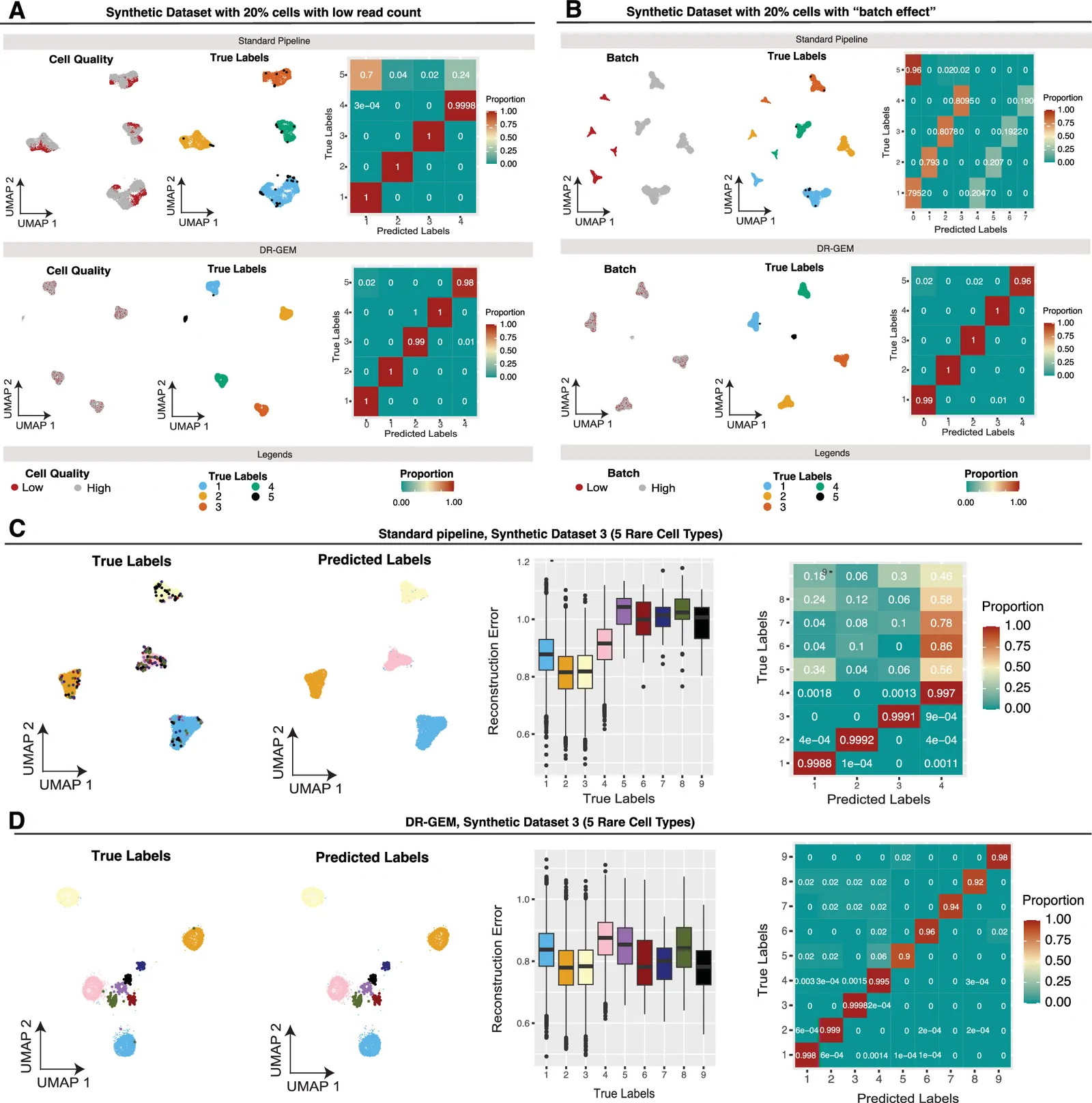
DR-GEM is performant on simulated datasets with technical noise and multiple types of rare cells. Performance of the standard pipeline (top row) and DR-GEM when applied to **A** synthetic dataset with latent class imbalance and 20% of cells simulated to have low read counts, and **B** Synthetic data with latent class imbalance and 20% of cells simulated to be impacted by batch effect: UMAP visualization of samples colored by ground truth synthetic cell type labels (left) and predicted cluster labels (middle), and proportion of predicted cell type (*x*-axis) in each of the true cell type populations (*y*-axis; right). Bottom row legends correspond to figures in the same row. Performance of standard pipeline (**C**) and DR-GEM (**D**) on Synthetic Dataset 3 with 5 rare cell types: UMAP visualization of samples colored by ground truth synthetic cell type labels (left) and predicted cluster labels (middle left), boxplots of RE (*y*-axis) in each ground truth synthetic cell type (*x*-axis, middle right), and proportion of predicted cell type (*x*-axis) in each of the true cell type populations (*y*-axis; right). **C**, **D** *n* = 21,750 cells, labeled as Cell Types 1–9 from the most to least abundant: *n* = 8000 (1), 5000 (2), 4500 (3), 4000 (4), and 50 (5-9); boxplots middle line: median; box edges: 25th and 75th percentiles; whiskers: most extreme points that do not exceed ± IQR x 1.5; further outliers are marked individually as black points.

To further test DR-GEM in the context of technical noise, we simulated two additional synthetic datasets with latent class imbalance, one where 20% of cells are simulated to have low read counts (Fig. 5A**, Methods**) and another where 20% of the cells are simulated to be impacted by batch effects (Fig. 5B**, Methods**). DR-GEM detected 98% and 96% of the rare Cell Type 5 when applied to the data simulated with low-quality cells and batch effects, while the standard pipeline falsely annotated all the rare cells in both settings (Fig. 5A, B).

Taken together, based on these simulations, transcriptomic profiles impacted by ambient RNA, low-read count, or batch effects are not enriched and are in fact more likely to be depleted, in the right tail of the RE distribution (Supplementary Fig. 7). Latent class imbalance remains the main correlate of high RE in each of these settings (*p* < 4 × 10^−^^14^, RE of rare vs. all other cell types, one-sided Wilcoxon rank-sum test in all noise-confounded synthetic datasets, Supplementary Fig. 7).

### DR-GEM is performant in detecting multiple types of rare cells

Complex tissues such as the tumor microenvironment or developing organs can often include multiple rare cell populations. To test whether DR-GEM can handle multiple rare populations simultaneously, we simulated datasets with multiple rare cell types.

First, the central hypothesis guiding DR-GEM’s design holds also in more complex settings with multiple types of rare cells. In a total of 500 synthetic datasets with either 2, 5, 10, 20, or 100 unique minority cell types, the mean RE is systematically higher for all minority cell types (*n* = 50 per population) compared with majority cell types (Supplementary Fig. 8). In accordance with this, the scRNA-seq data of tracheal epithelium^57^ includes 4 rare cell types – all 4 are most enriched in the right tail of the RE distribution (Supplementary Fig. 2F).

Second, when applied to a synthetic dataset with 5 distinct rare cell types (Synthetic Dataset 3, Fig. 5C, D), DR-GEM equalizes RE across all cell types and clusters all 5 rare cell types as their unique separate cluster, compared to the outputs of the standard pipeline, where the rare cell types do not form their own clusters and are scattered across the other clusters. DR-GEM correctly annotated 90%-98% of each of the five rare cell types, compared to 0% correct rare cell type annotations in the annotations obtained with the standard pipeline.

### DR-GEM is performant on tubo-ovarian cancer spatial transcriptomics

To test DR-GEM in end-to-end single-cell annotations in a real dataset with documented latent class imbalance and ambient RNA, we applied it to a single-cell spatial transcriptomics dataset of high-grade serous tubo-ovarian carcinoma (HGSC)^14^. The cell type annotations reported in the original paper were derived from a combination of manual curation and nested clustering, as well as data integration with scRNA-seq, tissue histology, and copy number alteration data, and are treated here as the ground truth labels.

As malignant cells can be highly heterogeneous, we applied DR-GEM in two ways: (1) only to non-malignant cells, and (2) to the entire dataset with annotations-in-the-loop. The latter was applied to examine whether annotations-in-the-loop can reorient DR-GEM to cell type level variance when applied to a dataset with high intra-cell-type variation.

**Fig. 6:**
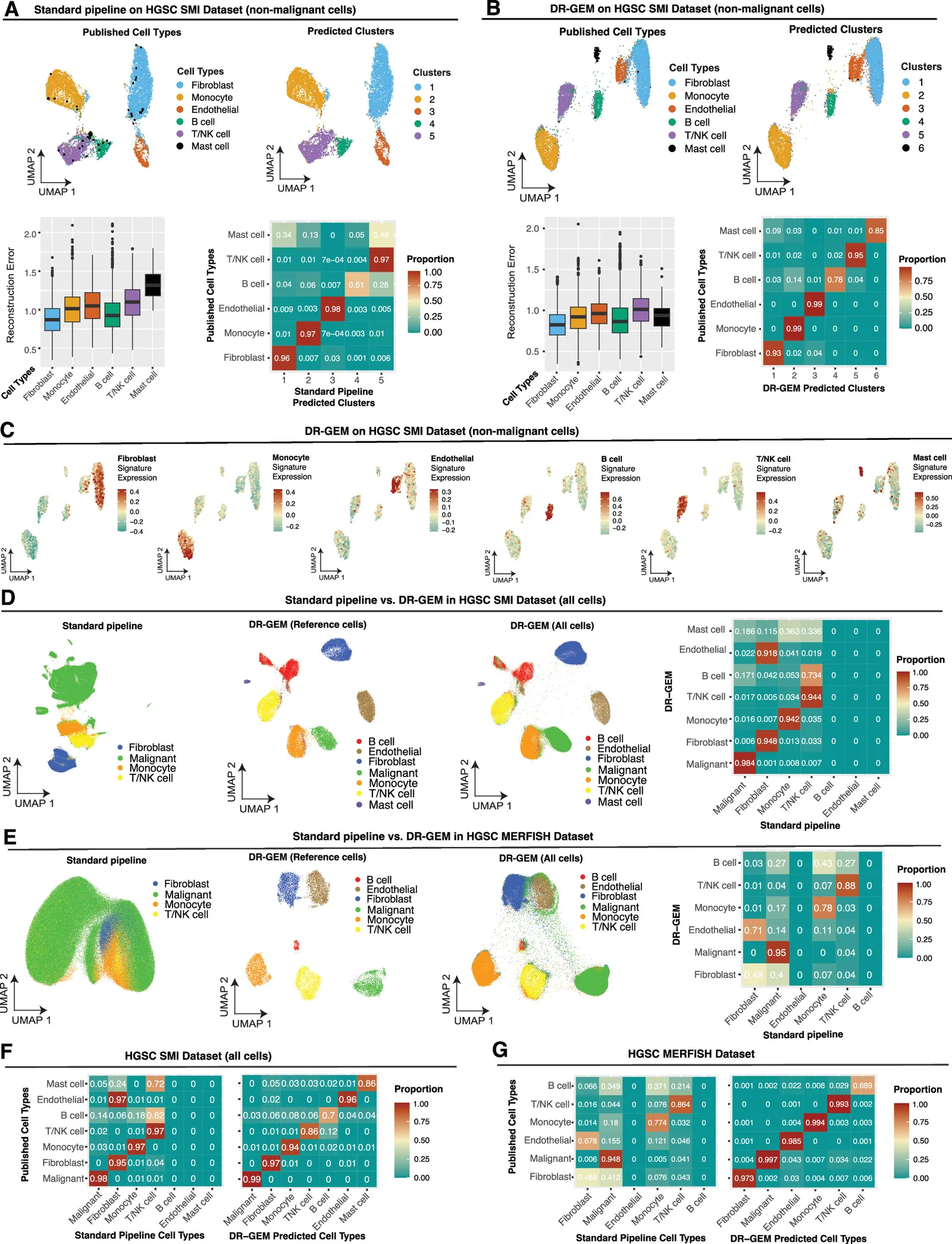
DR-GEM identifies rare cell types and mitigates ambient RNA in HGSC spatial transcriptomics. Performance of **A** standard pipeline and **B** DR-GEM when applied to non-malignant cells in the HGSC SMI dataset^14^. UMAP visualization of cells colored by published cell type labels (top left) and predicted clusters (top right), boxplots of RE (*y*-axis) in each cell type population (*x*-axis; bottom left), and proportion of predicted cell type (*x*-axis) in each of the published cell type populations (*y*-axis; bottom right). **C** UMAP visualization of HGSC SMI high-confidence cells in DR-GEM reference embedding. Cells colored by overall expression of cell type signatures derived from publicly available HGSC scRNA-seq datasets (**Methods**). Performance of standard pipeline and DR-GEM when applied to full HGSC SMI (**D**) and MERFISH (**E**) datasets. UMAP of cells colored by cell type labels derived from the standard pipeline (left); DR-GEM reference map colored by DR-GEM predicted cell types (middle left); DR-GEM reference map with all cells projected and colored by DR-GEM predicted cell types (middle right), proportion of standard pipeline cell type predictions (*x*-axis) in each of the DR-GEM cell type populations (*y*-axis; right). Proportion of standard pipeline (*x*-axis, left) and DR-GEM (*x*-axis, right) predicted cell types in the true cell type populations (*y*-axis) in HGSC (**F**) SMI, and (**G**) MERFISH datasets. **A**, **B** *n* = 82,808 cells: Fibroblast (*n* = 32,275), Monocyte (*n* = 22,791), Endothelial (*n* = 6,081), B cell (*n* = 7,705), T/NK cell (*n* = 13,627), Mast cell (*n* = 329); boxplot middle line: median; box edges: 25th and 75th percentiles; whiskers: most extreme points that do not exceed ± IQR x 1.5; further outliers are marked individually as black points. MERFISH multiplexed error-robust fluorescence in situ hybridization, NK natural killer, RE reconstruction error, SMI single-molecule imaging.

To examine performance in non-malignant cell annotations, we used the data profiled via the CosMx Single Molecule Imaging platform (SMI), sampled across 47 omentum metastatic HGSC tumors and 41 patients (**Methods**). Mast cells are the rarest cell type in this dataset, comprising 0.397% of the dataset. Analyzed with the standard pipeline, Mast cells are scattered across the embedding and do not form a Mast cell cluster, have significantly higher RE values compared to all other cell types (*p* = 5.08 × 10^−^^206^, *W* = 22135703, *r*_rb_ = 0.33, 95% CI [0.31, 0.34], RE of Mast cells vs. all other cell types, one-sided Wilcoxon rank-sum test), and are disproportionately enriched in the RE right tail (Fig. 6A; Supplementary Fig. 2A). Clustering followed by the Kuhn-Munkres matching algorithm identified one-to-one matching clusters corresponding to Fibroblasts (96% accuracy), Monocytes (97%), Endothelial cells (98%), B cells (61%), and T and Natural Killer (NK) cells (97%), but no cluster corresponded to Mast cells.

In contrast, with DR-GEM, Mast cells form a Mast cell cluster and RE values in this cluster are comparable to those of other cell types (Fig. 6B, p = 0.303, *W* = 620317, *r*_rb_ = 0.12, 95% CI [−0.02, 0.26], RE of Mast cells vs. all other cell types, one-sided Wilcoxon rank-sum test). Clustering under DR-GEM yielded one-to-one matching clusters corresponding to all cell types (Fig. 6B): Fibroblasts (93% accuracy), Monocytes (99%), Endothelial cells (99%), B cells (78%), T/NK cells (95%), and Mast cells (85%). DR-GEM also outperforms the standard pipeline on all balanced clustering metrics (**Methods**, Supplementary Data 2). Using cell type signatures previously derived from a meta-analysis^14^ of HGSC single-cell RNA-seq datasets, DR-GEM Phase IV correctly mapped the clusters to the matching cell types (Fig. 6C, **Methods**).

Similar performance improvements were observed when benchmarking DR-GEM (annotations-in-the-loop mode) using the full HGSC spatial transcriptomic data collected via SMI and MERFISH (Multiplexed Error-Robust Fluorescence In Situ Hybridization)^14^ (**Methods;** Fig. 6D–G). Applied to the SMI data, the standard pipeline detected only the top 4 most abundant cell types (Fig. 6D, F), while DR-GEM generated robust embeddings (Fig. 6D) and detected all 7 cell types (Fig. 6F), including the Endothelial, Mast, and B cells missed by the standard pipeline Applied to the MERFISH (**Methods**) data, the standard pipeline detected only the 4 most abundant cell types (Fig. 6E, G), while DR-GEM generated robust embeddings (Fig. 6E) and detected all 6 cell types (Fig. 6G), including the Endothelial and B cells missed by the standard pipeline.

### DR-GEM improves the embedding of cells from underrepresented patients and uncovers a spatially distinct malignant subclone

**Fig. 7:**
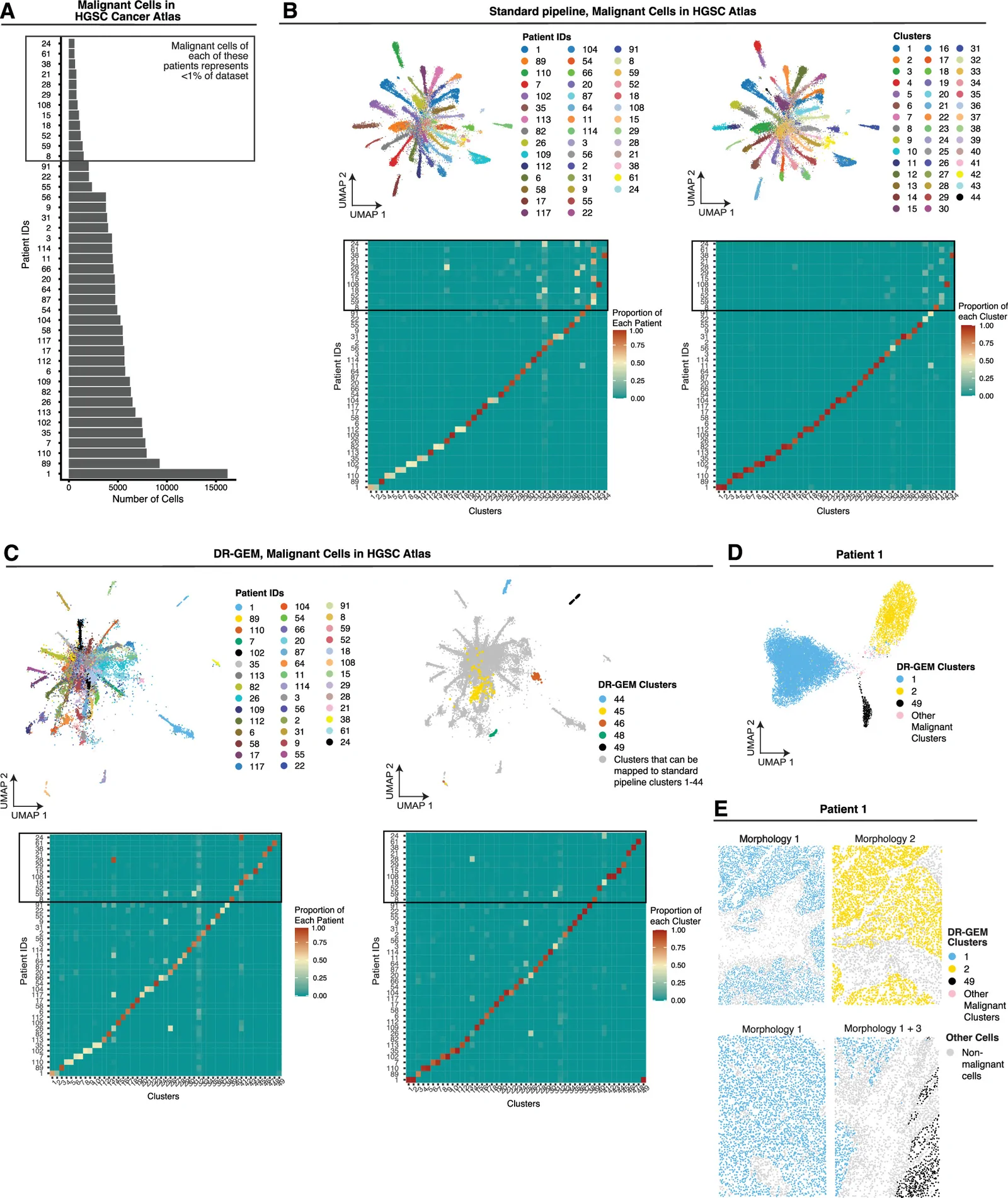
DR-GEM improves detection of rare patient-specific malignant cells and discovers a morphological subclone of malignant cells. **A** Number of malignant cells (*x*-axis) per patient. Black box indicates patients whose sampled malignant cells represent <1% of the malignant cells in the dataset. Standard pipeline (**B**) and DR-GEM performances (**C**): UMAP visualization of samples colored by patient ID (top left) and predicted cluster labels (top right), cluster proportions (fill color; *x*-axis) in each patient (*y*-axis; bottom left), and patient representation (fill color; *y*-axis) in each cluster (*x*-axis; bottom right). **D** UMAP of malignant cells from Patient #1, colored by DR-GEM’s predicted cluster. **E** Single cells plotted in situ of the 4 distinct fields of view from Patient #1 primary tumor, colored by DR-GEM cluster assignments. DR-GEM clusters 1, 2, and 49 correspond to 3 distinct morphological and spatial regions based on independent annotations by a board-certified pathologist (Methods). Source data are provided as a Source Data file.

Benchmarking DR-GEM in settings of genetic heterogeneity, we applied it to spatial transcriptomics (SMI) of malignant cells from HGSC adnexal tumors, spanning 185,071 cells from 43 patients^14^ (Fig. 7). In accordance with the well-documented pattern that malignant cells tend to cluster by patient, the embedding with the standard pipeline consists of 44 patient-specific clusters mapped to 34 out of the 43 patients, indicating that the remaining 9 patients do not have a unique malignant cell state. These 9 patients were also underrepresented in the data (<1% representation for each patient, Fig. 7A), such that amongst the 12 least represented patients (<1% representation for each patient), only 3 were represented by a patient-specific cluster (Fig. 7B). In contrast, with DR-GEM, 39 out of the 43 patients were represented by a patient-specific cluster, including 8 out of the 12 underrepresented patients (Fig. 7C), demonstrating the ability of DR-GEM to mitigate underrepresentation both at the cell and patient level. For one of the patients, DR-GEM detected 3 clusters versus 2 clusters detected with the standard pipeline (Fig. 7B-D). Projecting the cells back to the spatial coordinates shows that cluster 49 detected only with DR-GEM (*n* = 543 cells, 0.29% of all cells in the data) is spatially clustered and marks a morphologically distinct subclone previously identified and marked by a board-certified pathologist reviewing the matched H&E (Fig. 7D, E).

### DR-GEM is performant on mouse hypothalamus MERFISH data

To test DR-GEM on spatial transcriptomics data with well-defined cell types, we applied it to a MERFISH dataset of the mouse preoptic hypothalamus, which was previously annotated via an integrative approach, including data integration with scRNA-seq, nested clustering, and differential expression of specific marker genes^60^. The rarest cell type in this setting is the Pericyte, comprising 0.866% of the full dataset. These manually curated annotations were used here as the ground truth.

**Fig. 8:**
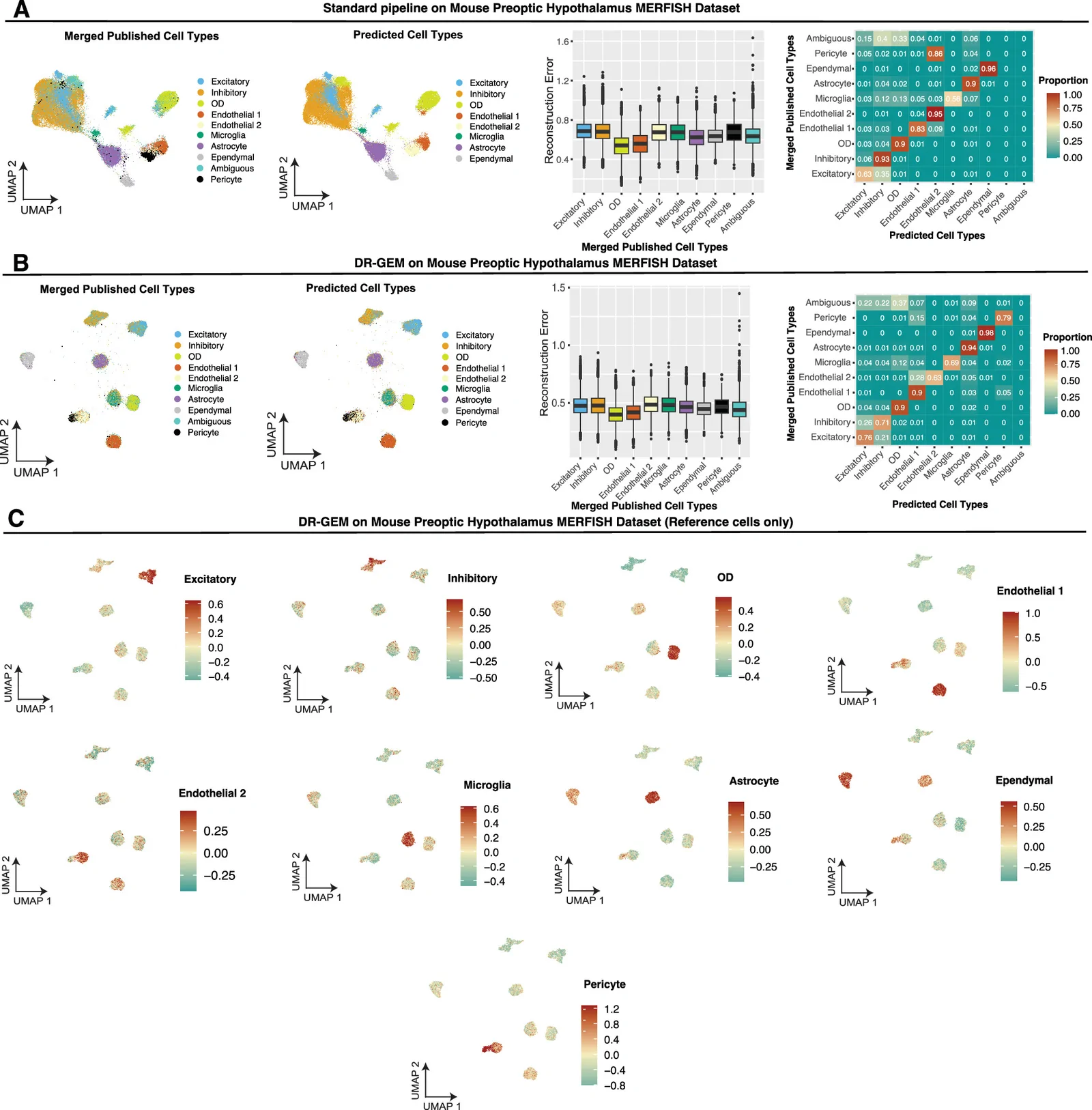
DR-GEM identifies rare cell types and mitigates ambient RNA in a mouse hypothalamus MERFISH dataset. Performance of standard pipeline (**A**) and DR-GEM (**B**) applied to MERFISH data (Animal 1) of the mouse preoptic hypothalamus^60^. UMAP visualization of cells colored by published cell type labels (left) and predicted cell type labels (middle left), boxplots of RE (*y*-axis) in each cell type (*x*-axis; middle right), and proportion of predicted cell type (*x*-axis) in each of the published cell type populations (*y*-axis; right). **C** DR-GEM reference map of the mouse preoptic hypothalamus MERFISH data (Animal 1). Cells colored by overall expression of cell type signatures derived from a paired scRNA-seq dataset of the mouse preoptic hypothalamus (**Methods**). **A**, **B** *n* = 73,655 cells: Excitatory (*n* = 11,757) neurons, Inhibitory (*n* = 24,761) neurons, Oligodendrocytes (OD, *n* = 9642), Endothelial 1 (*n* = 3799), Endothelial 2 (*n* = 1950), Microglia (*n* = 1472), Astrocyte (n = 8393), Ependymal (*n* = 1961), Pericytes (*n* = 638), Ambiguous (*n* = 9282); boxplots middle line: median; box edges: 25th and 75th percentiles; whiskers: most extreme points that do not exceed ± IQR x 1.5; further outliers are marked individually as black points. MERFISH multiplexed error-robust fluorescence in situ hybridization, OD oligodendrocyte, RE reconstruction error.

With the standard pipeline, the Pericytes are not clustered apart (Fig. 8A), have a significantly higher RE compared to all other cell types (*p* = 1.50 × 10^−3^, *W* = 203,841, *r*_rb_ = 0.24, 95% CI [0.20, 0.29], one-sided Wilcoxon rank-sum test), and are disproportionately enriched amongst the most poorly represented cells (Figs. 1F, 8A). There is a wide spread of RE for the cells previously labeled as Ambiguous (Figs. 1F, 8A). Cluster annotation predicted the following cell types: Excitatory neurons (63% accuracy), Inhibitory neurons (93%), Oligodendrocytes (OD; 90%), Endothelial 1 cells (83%), Endothelial 2 cells (95%), Microglia (56%), Astrocytes (90%), Ependymal cells (96%). None of the clusters map to Pericytes.

In contrast, with DR-GEM (annotations-in-the-loop mode), Pericytes form a distinct cluster (Fig. 8B, C) and have RE values comparable to those of all other cell types (*p* = 0.45, *W* = 1,923,741, *r*_rb_ = −0.05, 95% CI [−0.08, −0.04], one-sided Wilcoxon rank-sum test). DR-GEM yielded clusters with one-to-one matching to all cell types (Fig. 8B, C): Excitatory neurons (76% accuracy), Inhibitory neurons (71%), OD (90%), Endothelial 1 cells (90%), Endothelial 2 cells (63%), Microglia (69%), Astrocytes (94%), Ependymal cells (98%), and Pericytes (79%). In addition to the marked improvement in accuracy for Pericytes (0% to 79% accuracy), DR-GEM outperforms the standard pipeline on balanced clustering metrics (**Methods**, Supplementary Data 2). DR-GEM annotations show a more modest alignment with the original annotations for cells originally annotated as Inhibitory neurons and Endothelial 2 cells, potentially indicating that these cells may have been misannotated in the original study. Although cells labeled by the authors as Ambiguous were assigned to other cell types, their confidence score is on average lower than scores for all other cell types (*p* = 1.12 × 10^−^^177^, *W* = 244,539,325, *r*_rb_ = −0.88, 95% CI [−0.89, −0.87], one-sided Wilcoxon rank-sum test).

### DR-GEM captures responses to genetic perturbations in imbalanced Perturb-seq data

Perturb-seq datasets are becoming more widely available^10,11,14,78–80^ and often suffer from class imbalance. In a genome-scale CRISPR interference Perturb-seq dataset of K562 cells^10^ for example, the number of cells harboring each unique genetic perturbation ranges from 2 to 1963, while the number of cells with non-targeting control (NTC) sgRNAs is 75,305. We applied DR-GEM to this Perturb-seq dataset to test its ability to detect and label the transcriptional impact of a subset of perturbations vs. NTC cells. We selected representative perturbations across a spectrum of transcriptional impact magnitude (assessed based on the number of differentially expressed genes (#DEGs), **Methods**), biological functions, and representation in the dataset: *PTPN1* interference (*PTPN1*i, *n* = 371, 0.490% in dataset with NTC, #DEGs = 1844), MED19i (*n* = 299, 0.395%, #DEGs = 5528), SRRTi (*n* = 100, 0.133%, #DEGs = 1799), MRPL24i (*n* = 268, 0.355%, #DEGs = 1245), CASP8AP2i (*n* = 211, 0.279%, #DEGs = 1545), NRDE2i (*n* = 172, 0.228%, #DEGs = 1091).

**Fig. 9:**
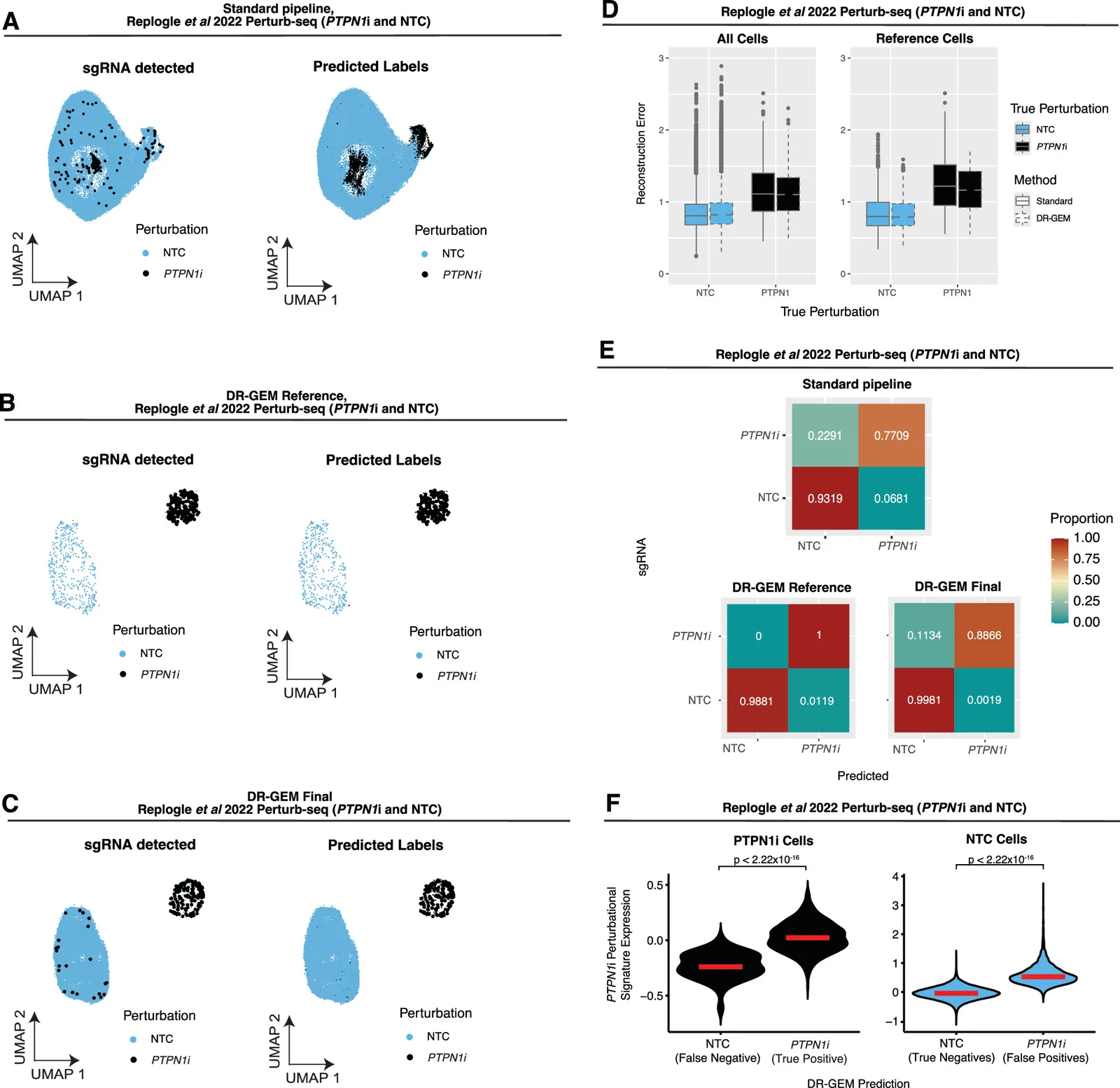
DR-GEM captures the transcriptional impact of *PTPN1* CRISPRi in Perturb-seq dataset. UMAP of K562 Perturb-seq data, showing NTC and PTPN1i cells colored by CRISPRi sgRNA detection (left) and predicted perturbations (right) in (**A**) all cells under the standard pipeline, **B** DR-GEM high-confidence reference cells, and **C** all cells with DR-GEM. **D** Boxplots of RE (*y*-axis) obtained with the Standard pipeline (solid line) and DR-GEM (dashed line), shown for all cells (left; *n* = 75,305 and 371 NTC and PTPN1i cells, respectively) or high-quality reference cells (right; *n* = 500 and 365 NTC and PTPN1i cells, respectively), stratified by sgRNA detection. Boxplot fill color corresponds to true perturbation. Boxplots middle line: median; box edges: 25th and 75th percentiles; whiskers: most extreme points that do not exceed ± IQR x 1.5; further outliers are marked individually as black points. **E** Proportion of predicted perturbations (*x*-axis) in PTPN1i and NTC cells (*y*-axis) when considering (top) all cells under the standard pipeline, (bottom left) high-confidence reference cells with DR-GEM, and (bottom right) all cells with DR-GEM. **F** Violin plots of PTPN1i signature expression (*y*-axis; **Methods**) stratified by DR-GEM prediction (*x*-axis): PTPN1i cells (left, *n* = 329 and 42 predicted as PTPN1i and NTC, respectively) and NTC cells (right, *n* = 75,162 and 143 predicted as NTC and PTPN1i, respectively). *p-*values derived from one-sided Student’s *t*-test. Red line denotes the median of each distribution. CRISPRi CRISPR interference, NTC non-targeting control, PTPN1i CRISPRi targeting *PTPN1*, RE reconstruction error, sgRNA single-guide RNA.

Analyzing PTPN1i and NTC Perturb-seq data, the UMAP visualization of the standard pipeline shows an embedding of cells that form a continuum rather than discrete clusters, with many of the PTPN1i cells scattered throughout the embedding (Fig. 9A). In contrast, with DR-GEM, there are two distinct clusters that show a clear 1-to-1 mapping to the genetic perturbation (Fig. 9B, C), with a tighter range of RE values for both PTPN1i and NTC cells (Fig. 9D). DR-GEM outperforms the standard pipeline across all balanced clustering metrics here as well (Supplementary Data 2). Using DR-GEM Phase IV to assign clusters to perturbation states shows that DR-GEM outperforms the standard pipeline in recovering ground truth annotations (Fig. 9E; DR-GEM: Accuracy = 1.00, Balanced Accuracy = 0.94, Specificity = 1.00, and Sensitivity = 0.89; Standard: Accuracy = 0.93, Balanced Accuracy = 0.85, Specificity = 0.93, and Sensitivity = 0.77).

Cells annotated in the Perturb-seq data as PTPN1i are cells in which PTPN1 sgRNA reads were detected. This annotation may be inaccurate due to ambient sgRNA and due to lack of functional interference of the target gene expression even in cells expressing the respective sgRNA. In addition, the response of cells to a genetic perturbation may be heterogeneous, resulting in visible effects on gene expression only in a subset of the perturbed cells. Using a signature of genes differentially expressed in PTPN1i vs. NTC cells shows that cells annotated as PTPN1i but predicted by DR-GEM as NTCs also have a lower expression of the PTPN1i perturbational signature compared to the other PTPN1i cells (*p* < 2.22 × 10^−^^16^, *t* (54.1) = 13.1, difference in means = 0.26, 95% CI [0.23, Inf], one-sided Student’s *t*-test; Fig. 9F, left). NTC cells predicted as PTPN1i cells based on DR-GEM have higher expression of the PTPN1i perturbational signature compared to the other NTC cells (*p* < 2.22 × 10^−16^, *t* (3972.5) = 84.4, difference in means = 0.73, 95% CI [0.71, Inf], one-sided Student’s *t*-test; Fig. 9F, right), suggesting that even in the absence of PTPN1 genetic interference some cells may exhibit a cell state similar to the PTPN1i perturbational state. Importantly, 24% of PTPN1i cells have low overall expression (OE < −0.1) of the PTPN1 perturbational signature, but 59% of these cells are nonetheless predicted by DR-GEM as PTPN1i, suggesting that DR-GEM captured perturbation effects not captured by standard differential gene expression analyses and DEG signatures.

**Fig. 10:**
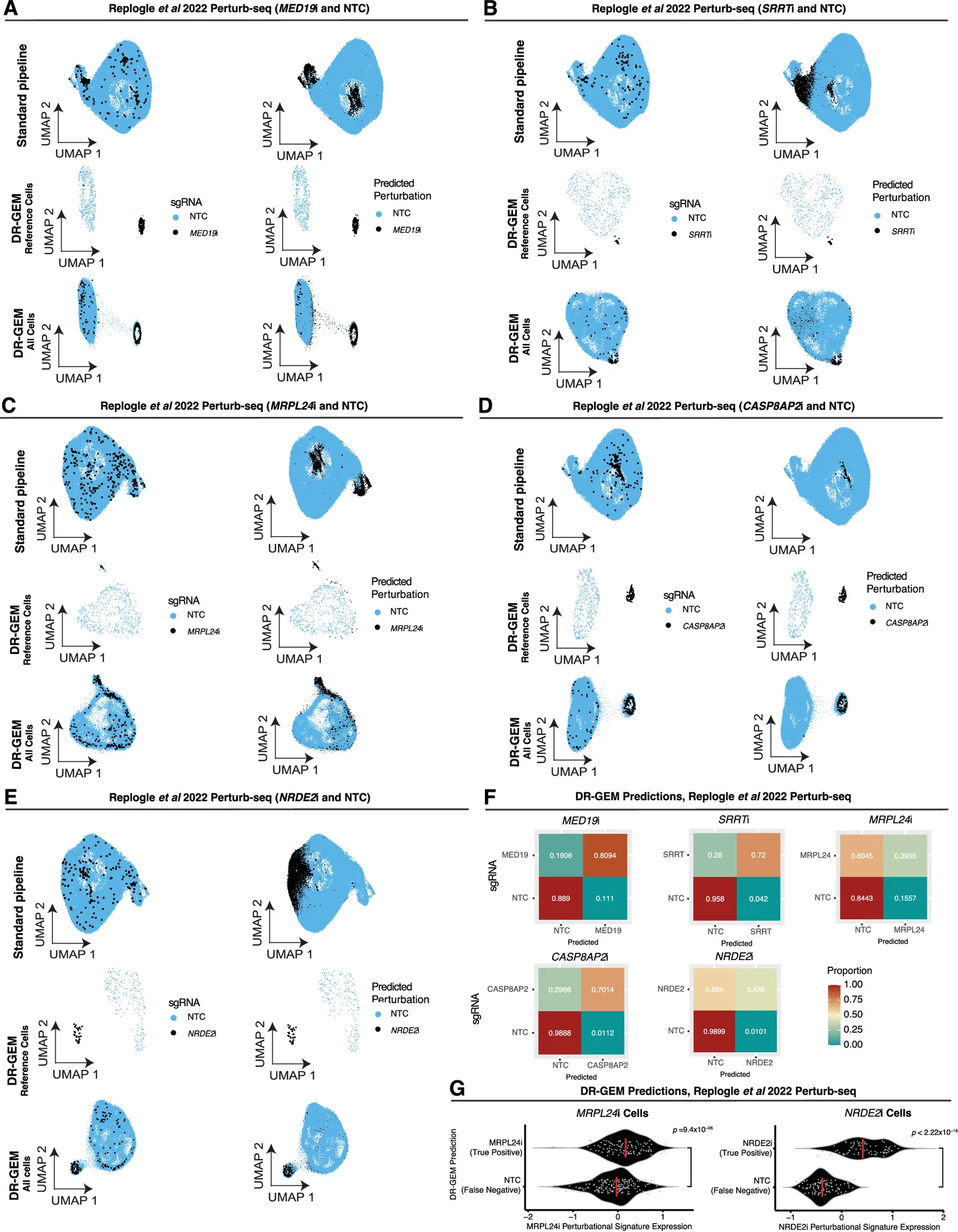
DR-GEM captures genetic perturbational states under extreme class imbalance. UMAP of K562 Perturb-seq data, showing NTC and **A** MED19i, **B** SRRTi, **C** MRPL24i, **D** CASP8AP2i, **E** NRDE2i cells colored by CRISPRi sgRNA detection (left) and predicted perturbations (right) in (top) all cells under the standard pipeline, (middle) high-confidence reference cells with DR-GEM, and (bottom) all cells with DR-GEM. **F** Proportion of DR-GEM-predicted perturbations (x-axis) in each of the true perturbation groups, as assigned based on sgRNA detection (y-axis). **G** Violin plots of (left) MRPL24i signature expression (*x*-axis) stratified by DR-GEM prediction (*y*-axis) in MRPL24i cells (*n* = 106 and 162 predicted as MRPL24i and NTC, respectively), and (right) NRDE2i signature expression (*x*-axis) stratified by DR-GEM prediction (*y*-axis) in NRDE2i cells (*n* = 75 and 97 predicted as NRDE2i and NTC, respectively). *p-*values were derived from a one-sided Student’s *t*-test. Red line denotes the median of each distribution. CRISPRi CRISPR interference, CASP8AP2i, MED19i, MRPL24i, NRDE2i, SRRTi, CRISPRi targeting *CASP8AP2*, *MED19*, *MRPL24*, *NRDE2*, and *SRRT*, respectively, NTC non-targeting control, sgRNA single-guide RNA.

Similar results were obtained for other genetic perturbations (Fig. 10), including MED19i (Fig. 10A), SRRTi (Fig. 10B), MRPL24i (Fig. 10C), CASP8AP2i (Fig. 10D), and NRDE2i cells (Fig. 10E). DR-GEM classifications outperform the standard pipeline in the prediction accuracy of the perturbed cells (Fig. 10F), Balanced Accuracy and Sensitivity across the whole dataset (Supplementary Fig. 9), and in all balanced clustering metrics (Supplementary Data 2). In the cases of MRPL24i and NRDE2i, DR-GEM detection of the perturbed cells was below 50%, yet false negative cells had a significantly lower expression of the respective perturbation signature compared to the true positive cells (MRPL24i*: p* = 9.4 × 10^−6^, *t* (236.5) = 4.5, difference in means = 0.24, 95% CI [0.15, Inf]; NRDE2i: *p* < 2.22 × 10^−16^, *t* (124.2) = 14.99, difference in means = 0.79, 95% CI [0.70, Inf], one-sided Student’s *t*-test; Fig. 10G), indicating that these cells may have not been subject to the perturbation despite the detection of the pertaining sgRNA.

These findings demonstrate how DR-GEM can be used to identify cells that were transcriptionally impacted by a genetic perturbation to better annotate Perturb-seq datasets in an unbiased manner, identify the cell state(s) elicited by genetic perturbations in a manner that is not fully captured by existing supervised (i.e., differential gene expression analyses) and unsupervised analyses (e.g., the standard embeddings and clustering), and help determine whether a genetic perturbation is eliciting a cell state(s) that is already manifested or absent in the control population.

### DR-GEM outperforms methods for rare cell type detection

Lastly, we benchmarked DR-GEM against three conceptually diverse end-to-end algorithmic frameworks for rare cell detection and/or cell type annotation: GiniClust3^47^ (the most recent of the GiniClust series), scCAD^48^, and CIARA^46^. While these methods were tested exclusively on scRNA-seq datasets, here we benchmarked performance across 9 different single-cell datasets and 14 rare cell detection tasks, spanning diverse biological contexts and data types (synthetic simulation, spatial transcriptomics, Perturb-seq, and scRNA-seq; Supplementary Data 2). DR-GEM outperformed the other three methods across 11 of 14 of the rare cell detection tasks (Supplementary Data 2).

Notably, when applied to the PBMC^63^ scRNA-seq dataset, scCAD outperformed all other methods. When applied to the developing thymus scRNA-seq dataset^56^, CIARA, a method built and tested on developmental datasets, outperformed all other methods. Yet all methods performed well on these two datasets. There were multiple instances where rare cells were completely missed by the other three methods. GiniClust3 did not detect any rare cells in the cystic fibrosis scRNA-seq data. CIARA did not detect any rare cells across all 6 Perturb-seq data subsets, as well as the MERFISH data, HNSCC, and Synthetic Data 2. scCAD did not detect any rare cells across all 6 Perturb-seq data subsets, as well as the HGSC spatial transcriptomics and Synthetic Datasets.

In summary, DR-GEM is non-inferior to GiniClust3, scCAD, and CIARA methods for the specific tasks of rare cell detection and annotations in scRNA-seq data, and significantly outperforms these methods when applied to synthetic, spatial transcriptomics, and Perturb-seq datasets. Yet, we note that, as shown throughout the study, suboptimal detection of rare cells is symptomatic of a more fundamental problem rooted in the model objective function, which is the focus of DR-GEM and this study.

## Discussion

Dimensionality reduction has become an integral part of biomedical research, providing input to downstream analyses and interpretation. The analytical and empirical results presented here show that dimensionality reduction methods can miss distinct patterns, not necessarily because the data are too sparse, but because standard objectives implicitly associate abundance with importance. This assumption can be counterproductive in basic and translational research. Less abundant cells, states, or molecules can have central roles in pathophysiological processes, and a model that captures a diversity of disease subtypes, cell states, or molecular classes can be more informative than one focused on the most frequent ones.

To address this gap, we developed DR-GEM, a framework that identifies and reorients the model’s attention to samples where performance is initially poor. Using the RE, DR-GEM identifies and upweights poorly embedded data points and subsequently uses the initial cluster assignments as weak labels to guide balanced consensus/ensemble learning to reorient the model to reproducible signal rather than stochastic noise. It thus takes principles often used in the context of supervised machine learning, such as distributionally robust optimization^20,28,29,81^, hard-example mining^82,83^, and boosting methods^84–86^ where a model detects and learns from its own errors.

DR-GEM improves upon the standard single-cell genomics analysis pipeline as well as DRO (KL-DRO^64^, CVaR-DRO^27^, Tilted Fair PCA^65^) and rare single-cell detection methods (GiniClust3^47^, scCAD^48^, and CIARA^46^) in faithfully reconstructing latent transcriptional structure and recovering held-out labels.

The self-supervision applied by DR-GEM is different from representational self-supervision, where models learn to perform a task of interest by training to predict parts of the data, as done in masked modeling (e.g., BERT^87^, masked autoencoder^88^), generative pre-training (GPT)^89^, and contrastive learning (e.g., SimCLR)^90^. Given DR-GEM performances when applied with the VAE-based method scVI (Supplementary Fig. 5C, D), an important direction will be to examine whether its error-guided learning strategy can complement foundation models in single-cell genomics and other settings where rare or underrepresented patterns are poorly learned. More broadly, DR-GEM suggests that some limitations attributed to insufficient data may be addressed by changing how the available data are modeled rather than by collecting more data, potentially providing a more cost-effective strategy for studying rare entities. Whether the same principle can also reduce the computational demands of foundation models, for example, by enabling informative patterns to be learned from fewer samples or training iterations, remains to be tested.

DR-GEM’s current limitations suggest directions for further development. Because Phase II fits and aggregates multiple balanced subsamples, its frequentist consensus-based approach is more computationally demanding than the commonly used single-pass embedding and clustering (**Supplementary Information**, Supplementary Data 4). While our analytical bounds and empirical findings indicate that a relatively small number of iterations is required to yield stable and reliable estimates (**Methods**, Supplementary Fig. 10), incorporating principles from online learning and adaptive optimization^91–93^ to update annotations dynamically as new data become available or as confidence scores start converging can help minimize computational cost. As with other dimensionality reduction methods, it is recommended to apply DR-GEM in a nested manner, first to define high-level clustering, and then separately for each cluster to define more fine-grained within-cluster heterogeneity. Automating these nested modes will be valuable for end-to-end embedding, clustering, and annotations across cluster levels.

Beyond single-cell biology, the core methodological principles of DR-GEM are applicable to other settings. In population-scale datasets like All of Us^94^, recent concerns over ancestry visualization using UMAP^95^ underscore how unsupervised embeddings can reinforce biases tied to race and ethnicity, with downstream implications for clinical AI. DR-GEM’s approach of refocusing learning on underrepresented subgroups offers a principled way to mitigate such biases. Similar opportunities exist in disease endotyping, such as in sepsis^96^, and schizophrenia^97,98^, to capture and better characterize rare subtypes, and in digital pathology^99,100^ and radiomics^101,102^, to reveal underappreciated morphological patterns in noisy image data. Similar principles may also be relevant to large protein models such as AlphaFold^103^ and ESM^104^, where some protein classes are better represented because they are easier to resolve experimentally or have more accessible evolutionary histories. These examples highlight the broader utility of incorporating self-supervision for latent class balancing in machine learning pipelines across biomedical research and biomedicine.

Together, DR-GEM addresses a fundamental limitation of dimensionality reduction: overfitting to dominant patterns while systematically misrepresenting unique ones. By grounding this observation in analytical theory and addressing it through a principled self-supervised framework, DR-GEM transforms a known statistical vulnerability into a correctable bias. As omics technologies scale to millions of cells and new modalities, and as single-cell methods move toward clinical deployment, the ability to faithfully represent rare and underrepresented populations will be increasingly consequential for data-driven discovery and applications. DR-GEM’s core principles can help advance the field toward richer and increasingly more meaningful representations of biology.

## Methods

### Analytical foundation for DR-GEM design: marker gene covariance is a function of cell type abundance

As we show below, the relative abundance of a cell type affects the covariance among its marker genes in the data, explaining the tendency of rare cell types to have higher RE values in Principal Component (PC) space. The PCs of a dataset are the eigenvectors of the original feature covariance matrix, sorted based on their eigenvalues, which are proportional to the variance explained by the respective PC.

The covariance of two genes *X* and *Y* in a population *P* of *N* cells can be represented as

1 $${Co}{v}_{P}\left(X,Y\right)={{\mathbb{E}}}_{P}\left[\left(X-{{\mathbb{E}}}_{P}\left(X\right)\right)\left(Y-{{\mathbb{E}}}_{P}\left(Y\right)\right)\right] \\={{\mathbb{E}}}_{P}\left[{XY}\right]-{{\mathbb{E}}}_{P}\left(X\right){{\mathbb{E}}}_{P}\left(Y\right)=A-B$$

For any split of population *P* to *G* groups of cells (*P*_*1*_*, … P*_*G*_) the term *A* can be represented as:

2 $$A={{\mathbb{E}}}_{P}\left[{XY}\right]={\sum}_{i\in G}{w}_{i}*{{\mathbb{E}}}_{{P}_{i}}\left[{XY}\right]={\sum}_{i\in G}{w}_{i}*\left({Co}{v}_{{P}_{i}}\left(X,Y\right)+{{\mathbb{E}}}_{{P}_{i}}\left[X\right]{{\mathbb{E}}}_{{P}_{i}}\left[Y\right]\right)$$

Where $${w}_{i}=\frac{\left|{P}_{i}\right|}{N}$$

As covariance is invariant to data centering, the property is analyzed when considering the data prior to centering. In the case that *X* and *Y* are marker genes of a specific group of cells *P*_*j*_, their average expression and covariance will be significantly lower in other groups of cells *P*_*i*_ (i≠j). By definition

3 $$\forall i\ne j:{Co}{v}_{{P}_{i}}\left(X,Y\right)\ll {Co}{v}_{{P}_{j}}\left(X,Y\right),{{\mathbb{E}}}_{{P}_{i}}\left[X\right]\ll {{\mathbb{E}}}_{{P}_{j}}\left[X\right],{{\mathbb{E}}}_{{P}_{i}}\left[Y\right]\ll {{\mathbb{E}}}_{{P}_{j}}\left[Y\right]$$

For simplicity, let the marker genes of population *j* be expressed only in *P*_*j*_.

4 $$\forall i\ne j:{Co}{v}_{{P}_{i}}\left(X,Y\right)=0,{{\mathbb{E}}}_{{P}_{i}}\left[X\right]=0,{{\mathbb{E}}}_{{P}_{i}}\left[Y\right]=0$$

*A* can be further simplified

5 $$A={{\mathbb{E}}}_{P}\left[{XY}\right]={\sum}_{i\in G}{w}_{i}*{{\mathbb{E}}}_{{P}_{i}}\left[{XY}\right]={w}_{j}*{{\mathbb{E}}}_{{P}_{j}}\left[{XY}\right]$$

And the average expression of a marker gene (e.g., *X*) in the whole population *P* is

6 $${{\mathbb{E}}}_{P}\left(X\right)={\sum}_{i\in G}{w}_{i}*{{\mathbb{E}}}_{{P}_{i}}\left(X\right)={w}_{j}*{{\mathbb{E}}}_{{P}_{j}}\left[X\right]$$

Together

7 $${Co}{v}_{P}\left(X,Y\right) 	={w}_{j} * \left({Co}{v}_{{P}_{j}}\left(X,Y\right)+{{\mathbb{E}}}_{{P}_{j}}\left[X\right]{{\mathbb{E}}}_{{P}_{j}}\left[Y\right]\right) \\ 	 -\left({w}_{j}*{{\mathbb{E}}}_{{P}_{j}}\left[X\right]\right)\left({w}_{j}*{{\mathbb{E}}}_{{P}_{j}}\left[Y\right]\right)$$

Let

8 $$C={{\mathbb{E}}}_{{P}_{j}}\left[X\right]{{\mathbb{E}}}_{{P}_{j}}\left[Y\right]$$

9 $${Co}{v}_{P}\left(X,Y\right)=A-B={w}_{j}*\left({Co}{v}_{{P}_{j}}\left(X,Y\right)+C\right)-{w}_{j}^{2}*C$$

$$={w}_{j}*{Co}{v}_{{P}_{j}}\left(X,Y\right)+{w}_{j}\left(1-{w}_{j}\right)*C$$

The covariance can thus be represented as a weighted average of marker gene strength and within cell type covariance

10 $${Co}{v}_{P}\left(X,Y\right)=\underbrace{{w}_{j} * {Co}{v}_{{P}_{j}}\left(X,Y\right)}_{\begin{array}{c}{Within\; cell\; type}\\ {covariance}\end{array}} \!\!\!+ \!\underbrace{{w}_{j}\left(1-{w}_{j}\right) * {{\mathbb{E}}}_{{P}_{j}}\left[X\right]\,{{\mathbb{E}}}_{{P}_{j}}\left[Y\right]}_{{Cell\; type\; gene\; marker\; strength}}$$

The term ${w}_{j}(1-{w}_{j})$ is at most 0.25 (when *w*_*j*_ = 0.5). It is monotonically increasing with *w*_*j*_ when ${w}_{j}\in \left[{{\mathrm{0,0.5}}}\right)$ and monotonically decreasing with *w*_*j*_ when ${w}_{j}\in \left({{\mathrm{0.5,1}}}\right]$. As the data is not centered, C≥0 and the covariance between marker genes in the relevant cell type population is expected to be positive, i.e., ${Co}{v}_{{P}_{j}}\left(X,Y\right)\ge 0$. Thus, for ${w}_{j}\in \left[{{\mathrm{0,0.5}}}\right)$ the lower *w*_*j*_ the lower the covariance of *X* and *Y* is in the entire population. When ${w}_{j}\in \left({{\mathrm{0.5,1}}}\right]$ the covariance of marker genes of population *P*_*j*_ depends more on their covariance within *P*_*j*_, and less so on the level of their expression in the cell type they mark. If the marker genes of *P*_*j*_ do not covary within *P*_*j*_, then their covariance will drop with the increase in *w*_*j*_ once *w*_*j*_ > 0.5. *w*_*j*_ = 0.5 thus represents a tipping point, above which PCA will focus more on the within (dominant) cell type variation compared to the cross-cell-type variation.

By rebalancing the data, DR-GEM hence obtains a clearer distinction between cell types for both the rare and more abundant cell types, consistent with the notion that the former is blended with the latter in unbalanced settings. This also demonstrates why a subsequent embedding per cell type population is needed, where the intra-cell-type covariation can be captured more accurately without being tied to cross-cell-type variation. DR-GEM improves cell type annotations and hence downstream cell-type-specific analyses will be less likely to be contaminated by signals coming from other cell types as misassigned rare cells.

### Synthetic data generation

Synthetic single-cell ‘omics datasets were generated using a custom simulation framework. Each dataset consists of count matrices simulating gene expression across *K* discrete cell types/states, with parameters selected to reflect common features of real-world data, such as cell type and cell type invariant variation, dropout events, overdispersion, and class imbalance with rare populations.

Gene expression counts were simulated using a two-level negative binomial sampling process. First, for each gene $j\in \left\{1,\ldots,{p}_{1}\right\}$, and cell type $k\in \{1,\ldots,K\}$, a mean expression value was drawn from a negative binomial distribution:

11 $${\mu }_{{jk}}{{{\mathscr{\sim }}}}{{{\mathcal{N}}}}{{{\mathcal{B}}}}\left({\mu }_{1},{{\sigma }_{1}}^{2}\right)$$

Where ${\mu }_{1}=2$ and ${{\sigma }_{1}}^{2}$ = 0.15. In this manner cells of the same cell type overexpress the same marker genes (i.e., genes with a high ${\mu }_{{jk}}$). Then, for each cell $i=1,\ldots,{N}_{k}$, counts were sampled gene-wise from a negative binomial distribution with cell type specific parameters conditioned on the previously drawn gene-level:

12 $${x}_{{ijk}}{{{\mathscr{\sim }}}}{{{\mathcal{N}}}}{{{\mathcal{B}}}}\left({\mu }_{{jk}},{{\sigma }_{2}}^{2}\right)$$

Where ${{\sigma }_{2}}^{2}$ = 0.5. This process was repeated independently for each cell type $k\in \left\{1,\ldots,K\right\},$ where ${N}_{1},\ldots,{N}_{K}$ cells were sampled from each cell type *k*, respectively (where ${N}_{1}+\ldots+{N}_{K}=N$) yielding a combined count matrix ${X}_{{markers}}\in {{\mathbb{R}}}^{N\times {p}_{1}}$ with known cell type labels.

An additional set of *p*_2_ features (where *p*_*2*_ < *p*_*1*,_ and *p* = *p*_*1*_ + *p*_*2*_) of cell-type independent variation was sampled from an analogous two-level negative binomial sampling process. This time, cells were randomly assigned to *K*_*2*_ cell states independent of the *K* cell type assignments to generate ${X}_{{states}}\in {{\mathbb{R}}}^{N\times {p}_{2}}$.

Concatenating *X*_*markers*_ and *X*_*states*_ yielded counts matrix ${X}_{{{{\rm{Clean}}}}}\in {{\mathbb{R}}}^{N\times p}$.

We simulated negative binomial noise matrix $\varepsilon \in {{\mathbb{R}}}^{N\times p}$ with ${\mu }_{2}=2$ and ${{\sigma }_{3}}^{2}=0.15$ such that:

13 $${\varepsilon }_{{ijk}}{{{\mathscr{\sim }}}}{{{\mathcal{N}}}}{{{\mathcal{B}}}}\left({\mu }_{2},{{\sigma }_{3}}^{2}\right)$$

Random dropout was modeled as a binary dropout mask, $D\in {\{{{\mathrm{0,1}}}\}}^{N\times p}$, generated from a Bernoulli distribution (dropout probability δ = 0.05) such that:

14 $${D}_{{ij}}\sim {{\rm{Bernoulli}}}\,\left(1-{{\delta }}\right)$$

Applying these simulated sources of noise yields a final synthetic counts matrix, $X\in {{\mathbb{R}}}^{N\times p}$:

15 $$X=D\circ \left({X}_{{{\rm{Clean}}}}+{{\varepsilon }}\right)$$

All simulations were performed with a fixed random seed for reproducibility. For subsequent testing and analysis, all simulated datasets were log-normalized and scaled.

### Standard pipeline for single-cell annotations

The standard pipeline for single-cell annotation^63^ comprises three main steps: (1) dimensionality reduction, most commonly via PCA, (2) clustering of the first PCs, (3) UMAP visualization, and (4) cluster-based annotation based on marker or signature expression.

RE was computed based on the m-dimensional PC representation. More specifically, PCA can be solved analytically via singular value decomposition (SVD) such that for input data $X\in {{\mathbb{R}}}^{N\times p}$

16 $$X=U\Sigma {V}^{{{\top }}}$$

Where $U\in {{\mathbb{R}}}^{N\times N}$ contains the left singular vectors, $\Sigma \in {{\mathbb{R}}}^{N\times p}$ is a diagonal matrix of singular values, and ${{{\rm{V}}}}\in {{\mathbb{R}}}^{p\times p}$ contains the right singular vectors. The lower-dimensional embedding $Z\in {{\mathbb{R}}}^{N\times m}$ is obtained from the top m principal components such that:

17 $$Z=X{V}_{m}={U}_{m}{\Sigma }_{m}$$

This then yields the rank-m approximation (reconstruction) of X:

18 $$\hat{X}=Z{V}_{m}^{{{\top }}}={U}_{m}{\Sigma }_{m}{V}_{m}^{{{\top }}}$$

The standard mean squared error (MSE) as RE *e*_*i*_ was calculated such that for each sample i:

19 $${e}_{i}=\frac{1}{p} {\sum}_{j=1}^{p}{\left({x}_{{ij}}-{\hat{x}}_{{ij}}\right)}^{2}$$

Standard PCA was performed using the Seurat (v5) R package. The number of PCs retained for each real-world dataset (*m*) was selected using the conventional heuristic of identifying the elbow point in the variance-explained plot, representing the inflection at which additional components contribute diminishing returns in explained variance.

### Simulation experiments of synthetic class imbalance

To systematically assess the impact of class imbalance, a series of synthetic datasets were simulated with varying levels of class imbalance. Specifically, five discrete cell types (*K* = 5) were simulated with fixed sample sizes for four of the populations (*N*_1_ = 8000, *N*_2_ = 5000, *N*_3_ = 4500, *N*_4_ = 4000) and a variable number of samples for the fifth (rare) cell type: ${N}_{5}\in \{4000,2000,1000,500,250,125,50,25\}$.

For each of the imbalance settings, 100 independent synthetic datasets were generated with *p*_1_ = 150 and *p*_2_ = 50 and a total of *p* = 200 genes using 100 unique random seeds with the two-level generative negative binomial model described in the previous section. Noise (ε) and dropouts (*D*) were independently simulated and applied to each dataset.

Each dataset was log-normalized and scaled before application of PCA. RE (${{{\boldsymbol{e}}}}$) was computed as the MSE between the scaled expression data and the reconstruction of the data from the top 10 PCs (*m* = 10). *m* was chosen here based on the elbow criterion over 100 datasets where *N*_*5*_ = 4000.

For each simulated dataset and for each cell type, the maximum, mean, and minimum RE were calculated and recorded. Furthermore, the enrichment of each cell type among the cells with the highest RE values (top 10%) was computed based on the observed to expected fold-change (FC):

20 $${{{\rm{FC}}}}=\frac{{{{\rm{cell}}}}\,{{{\rm{type}}}}\,{k}\,{{{\rm{proportion}}}}\,{{{\rm{in}}}}\,{{{\rm{the}}}}\,{{{\rm{top}}}}\,{{{\rm{decile}}}}\,{{{\rm{of}}}}\,{{{\rm{the}}}}\,{{{\rm{RE}}}}\,{{{\rm{distribution}}}}}{{{{\rm{proportion}}}}\,{{{\rm{of}}}}\,{{{\rm{cell}}}}\,{{{\rm{type}}}}\,{k}\,{{{\rm{in}}}}\,{{{\rm{the}}}}\,{{{\rm{data}}}}}$$

### Synthetic Dataset 1

A representative baseline synthetic dataset was utilized as a controlled setting to test different algorithms. This dataset was simulated with the following parameters: *N* = 21,550, *p* = 200, *p*_1_ = 150, *p*_2_ = 50, *K* = 5, *N*_1_ = 8000, *N*_2_ = 5000, *N*_3_ = 4500, *N*_4_ = 4000, *N*_5_ = 50, ${\mu }_{1}$ = 2, ${{\sigma }_{1}}^{2}$ = 0.15, ${{\sigma }_{2}}^{2}$ = 0.5. A simulated noise matrix ($\varepsilon,\,{{{\mu }_{2}=2,\sigma }_{3}}^{2}=0.15$) and dropout mask (*D*, ${{{\rm{\delta }}}}=0.05$) were also applied.

### PCA as a linear autoencoder

PCA was implemented as a single-layer linear autoencoder in order to modify the default loss function and train the model with stochastic gradient descent (Fig. 2A). It has been previously shown that an autoencoder with a single fully connected hidden layer, a linear activation function, and a squared error cost function (RE) trains weights that span the same subspace as the one spanned by the PC loading vector^105,106^. The loading vectors equivalent to those solved via SVD can be recovered from the autoencoder weights. In other words, for an input data matrix $X\in {{\mathbb{R}}}^{n\times p}$, where a m-dimensional representation $Z\in {{\mathbb{R}}}^{n\times m}$ is obtained (where m<p) and linear activation weights matrix $W\in {{\mathbb{R}}}^{p\times m}$ by minimizing mean-squared error as RE such that:

21 $${\min}_{W}||X-Z{W}^{+}|{|}_{F}^{2}\,{{{\rm{subject }}}}\,{{{\rm{to}}}}\,Z={XW},{W}^{+}W={I}_{m}$$

where ${W}^{+}$ is pseudoinverse for W. It is equivalent to solving for ${V}_{m}$ using the analytical approach for standard PCA.

The implementation of PCA as a linear autoencoder utilized the autoencoder framework in PyTorch^107^ with a single fully connected linear layer of m-dimensions. The main training routine receives the model architecture with default-initialized weights and a specific loss function (either standard RE or DRO objectives, described next). Models were trained over mini-batches using the Adam optimizer^108,109^ with a fixed weight decay for regularization. Optimization was conducted over user-defined epochs to reach convergence. Model evaluation was conducted from analysis of RE (${{{\boldsymbol{e}}}}\in {{\mathbb{R}}}^{n}$) as defined above.

### Linear autoencoder with KL-DRO

In one approach to promote fairness and robustness for PCA, KL-DRO^64^ objective was applied for PCA as a Linear Autoencoder. In this setting the standard RE is defined as:

22 $${\ell}\left(W,X\right)={\sum}_{{{{\boldsymbol{x}}}}\in X}||{{{\boldsymbol{x}}}}-W{W}^{+}{{{\boldsymbol{x}}}}|{|}_{2}^{2}$$

and the KL-DRO loss is defined as:

23 $${L}_{{{\rm{KL}}}-{{\rm{DRO}}},\lambda }\left(W,X\right)={\max }_{{{{\boldsymbol{p}}}}\in \Delta } {\sum}_{{{{\boldsymbol{x}}}}\in X}{p}_{{{{\boldsymbol{x}}}}}{\ell}\left(W,{{{\boldsymbol{x}}}}\right)-\lambda \,{{{\rm{KL}}}}\left({{{\boldsymbol{p}}}},\frac{1}{N}\right)$$

Where ${{{\rm{KL}}}}(\cdot,\cdot )$ is the Kullback-Leibler divergence between two distributions and λ regularization hyperparameter controlling robustness. This loss has been shown to be equivalent to^30^:

24 $${L}_{{{\rm{KL}}}-{{\rm{DRO}}},\lambda }\left(W,X\right)=\lambda \log \left(\frac{1}{N} {\sum}_{{{{\boldsymbol{x}}}}\in X}\exp \left(\frac{{\ell}\left(W,{{{\boldsymbol{x}}}}\right)}{\lambda }\right)\right)$$

KL-DRO and tilted empirical risk minimization^65^ are mathematically equivalent under a specific transformation of parameters. Both frameworks prioritize worst-case or rare examples by reweighting the empirical loss function in a tilted manner (Fig. 2A).

The hyperparameter λ was tuned by testing a set of values in PCA as a Linear Autoencoder:

$$\left\{0.0001,0.0005,0.001,0.005,0.01,0.05,0.1,0.5,1,5,10,50\right\}$$

The value λ that best minimized maximum RE without a high cost to overall RE (Fig. 2B) in the dataset was chosen (λ=0.001).

### Linear autoencoder with CVaR-DRO

An alternative formulation of DRO is CVaR^27^, a more classic approach with initial roots in finance and economics. CVaR-DRO loss for PCA as a Linear Autoencoder is as follows:

25 $${L}_{{{\rm{CVaR}}}-{{\rm{DRO}}},\beta }\left(W,X\right)={\max }_{{{{\bf{p}}}}\in \Delta } {\sum}_{{{{\bf{x}}}}\in X}{p}_{{{{\bf{x}}}}}\,{\ell}\left(W,{{{\bf{x}}}}\right)-{{{\rm{D}}}}_{{{\rm{CVaR}}},\beta }\left({{{\bf{p}}}},\frac{1}{N}\right)$$

where ${{{\rm{D}}}}_{{{\rm{CVaR}}},\beta }$ is the CVaR divergence. The loss can also be represented as:

26 $${{{L}}}_{{{\rm{CVaR}}}-{{\rm{DRO}}},\beta }\left({{W}},{{X}}\right)={t}_{\beta }+\frac{1}{{{N}}{{\beta }}} {\sum}_{{{{\rm{x}}}}\in {{{\rm{X}}}}}{\left({\ell}\left(W,{{{\rm{x}}}}\right)-{t}_{\beta }\right)}_{+}$$

Where ${t}_{\beta }$ is the threshold value that partitions the data to identify the top *β* fraction of reconstruction values^27^. CVaR focuses on minimizing the worst-case (highest) losses — specifically, the average loss in the highest *β*-quantile.

The hyperparameter β was tuned by testing a set of values in PCA as a Linear Autoencoder:

$$\left\{0.001,0.005,0.01,0.05,0.1,\,0.15,\,0.2,\,0.25,\,0.3,\,0.35\right\}$$

The value β that best minimized maximum RE without much cost to overall RE in Synthetic Dataset 1 was β=0.005 (Fig. 2B).

### Fair PCA

To assess a dimensionality reduction method where group-wise fairness is enforced, the multiple-objective optimization of Fair PCA with tilted reweighting was implemented. This scheme explicitly aims to balance RE across predefined groups. The input dataset $X\in {{\mathbb{R}}}^{n\times p}$ is partitioned into its known *K* groups $\{{X}_{1},{X}_{2},\ldots,{X}_{K}\}$ where ${X}_{k}\in {R}^{{N}_{k}\times p}$ contains all ${N}_{k}$ cells from group *k*. For an orthonormal projection matrix ${V}_{m}\in {{\mathbb{R}}}^{p\times m}$ fit from standard PCA, the RE for group *k* is:

27 $${{{{\boldsymbol{e}}}}}_{k,{V}_{m}}=\frac{1}{{N}_{k}}||{X}_{k}-{X}_{k}{V}_{m}{{V}_{m}}^{{{\top }}}{||}_{F}^{2}$$

Let ${V}_{m}^{(k)}\in {{\mathbb{R}}}^{p\times m}$ be the projection matrix obtained by performing PCA only on group *k*, minimizing RE only within group *k*. The disparity error for group *k* is then defined as:

28 $${{\varepsilon }}^{(k)}={{{{\boldsymbol{e}}}}}_{k,{V}_{m}}-{{{{\boldsymbol{e}}}}}_{k,{V}_{m}^{(k)}}$$

A projection ${V}_{m}$ is said to be fair if all groups experience the same disparity error:

29 $${{\varepsilon }}^{(1)}={{\varepsilon }}^{(2)}=\ldots={{\varepsilon }}^{(K)}$$

Because exact equality is difficult to achieve in practice, this requirement is relaxed, and the strength of Pareto parity^34^ is enforced by the hyperparameter *t*. Larger values of *t* force the losses on groups to be as close as possible, whereas lower values of *t* offer the flexibility of reducing the performance gap between groups less aggressively.

30 $${L}_{{{{\rm{FairPCA}}}}}\left({V}_{m},X\right)=\frac{1}{t}\log \frac{1}{N} {\sum}_{k=1}^{K}\exp \left(t\left({{\varepsilon }}^{\left(k\right)}\right)\right)$$

Fair PCA models were fit by applying an adapted version of gradient-based multi-objective optimization that has been previously described and implemented^32^. The hyperparameter t was tuned by testing the following hyperparameter values:

$$\left\{5,10,20,50,100,200\right\}$$

For application on Synthetic Dataset 1, *t* = 10 was sufficient to achieve a level of group-wise parity that successfully minimized the rare cell type RE. The group information was provided to Fair PCA, thus providing latent ground truth information to examine performance in the most favorable conditions that do not recapitulate application in real-world data.

### DR-GEM algorithm

DR-GEM is a meta-algorithm that uses the RE and cluster assignment consistency for self-supervision to obtain distributionally robust representations optimized at the level of latent classes.

#### Phase I: Distributional robust initialization

Let $X\in {{\mathbb{R}}}^{n\times p}$ be the normalized and scaled gene expression matrix of n cells and p genes; it is assumed that DR-GEM takes as input a gene expression matrix with standard pre-processing such as filtering of empty droplets or dying cells. DR-GEM begins by fitting a standard unsupervised embedding ${E}^{\left(0\right)}\in {{\mathbb{R}}}^{n\times m}$ using a user-defined dimensionality reduction method (e.g., PCA), where *m* is user-defined (e.g., based on the elbow criteria). $\hat{X}\in {{\mathbb{R}}}^{n\times p}$ is the reconstructed gene expression matrix from ${E}^{\left(0\right)}$. For each cell i∈1,...,n, a RE (${{{\boldsymbol{e}}}}\in {{\mathbb{R}}}^{n}$) is computed as the MSE between each row of X and $\hat{X}$. Cells are ranked by RE, and those at the top β percentile (user-defined, default β=0.05) are selected as the high-risk subset (${n}^{\left(0\right)}=\beta \cdot n$). A second embedding ${E}^{\left({init}\right)}\in {{\mathbb{R}}}^{{n}^{(0)}\times m}$ is learned on this subset (defined by β) alone. The remaining cells are projected onto the subspace spanned by ${E}^{\left({init}\right)}$, using the projection matrix obtained by fitting ${E}^{\left({init}\right)}$ to yield ${E}^{\left(1\right)}\in {{\mathbb{R}}}^{n\times m}$. Clustering is then applied to ${E}^{\left(1\right)}$, producing an initial label vector ${{{{\boldsymbol{c}}}}}^{\left(1\right)}\in \{1,\ldots,K\}^{n}$, referred to as initialized weak cluster labels.

#### Phase II: Balanced consensus learning

DR-GEM obtains *r* (user-defined) embeddings that yield *r* sets of cluster assignments, each based on one of *r* balanced subsamples of the data. The subsample of the dataset is drawn by uniformly sampling from each weak cluster in ${{{{\boldsymbol{c}}}}}^{\left(1\right)}$, thereby balancing the cluster sizes. The uniform sampling procedure is performed by randomly sampling $\min \left(b,{N}_{k}\right)$ cells from each cluster *k*, where b is a user-defined parameter (default *b* = 500) and ${N}_{k}$ is the number of cells in each weak cluster *k*. Let ${X}^{\left(2,r\right)}\subset X$ denote a balanced subsample of size ${n}^{(2,r)}$, dimensionality reduction projects ${X}^{\left(2,r\right)}$ to an *m*-dimensional embedding: ${E}^{\left(2,r\right)}\in {{\mathbb{R}}}^{{n}^{(2,r)}\times m}$, which undergoes subsequent clustering. Because cluster identities can be permuted between runs, labels are aligned to ${{{{\boldsymbol{c}}}}}^{\left(1\right)}$ using the Kuhn–Munkres algorithm^67^, also known as the Hungarian algorithm, via the lp.assign() function in the lpSolve R package. The Kuhn-Munkres algorithm takes as input the contingency table between two sets of categorical label predictions and determines a one-to-one label mapping. If an iteration results in a larger number of clusters compared to the number of weak labels, then multiple clusters can map to the same available weak cluster; if fewer clusters in an iteration are produced than the number of weak labels, then one or more of the weak clusters will be unmatched. Phase II yields an ensemble of cluster assignments for each cell *i*: ${{{{\boldsymbol{c}}}}}_{i}^{(2)}=\{{c}_{i,1}^{\left(2\right)},\ldots,{c}_{i,{\rho }_{i}}^{(2)}\}$ where ${\rho }_{i}$ is the number of times cell *i* was sampled across the *r* iterations.

#### Phase III: Aggregation and high confidence reference mapping

The final cluster label ${\hat{c}}_{i}$ for each cell *i* is assigned via majority vote across the ensemble ${c}_{i}^{\left(2\right)}={c}_{i,1}^{\left(2\right)},\ldots,{c}_{i,{\rho }_{i}}^{\left(2\right)}$. A confidence score ${s}_{i}\in \left[{{\mathrm{0,1}}}\right]$ is defined as the proportion of iterations in which cell *i* was assigned to ${\hat{c}}_{i}$ across all ${\rho }_{i}$ iterations. A final reference embedding is constructed using a cluster-balanced subset of high-confidence cells, defined as the ${n}_{t}$ cells with ${s}_{i} > t$, where t is a user-defined threshold (default: t = 0.95). The reference embedding is computed using the same dimensionality reduction method used in previous phases to yield a reference embedding ${E}^{\left({{{\rm{ref}}}}\right)}\in {{\mathbb{R}}}^{{n}_{t}\times m}$. All cells are then projected into this reference subspace to yield embeddings: ${E}^{\left({{{\rm{final}}}}\right)}\in {{\mathbb{R}}}^{n\times m}$ for final visualization and analysis.

#### Optional Phase IV: Signature-based annotation

DR-GEM provides optional functionality for supervised cluster annotation using curated gene signatures. The Overall Expression (OE)^14^ of each signature is computed for each cell. A cluster is assigned to a specific signature if and only if the cells assigned to it have a significantly higher OE of the signature (*t*-test *p* < 1 × 10^-10^, one-sided) compared to all other clusters. Clusters that do not meet these criteria are labeled as Undetermined. Clusters with overexpression of more than one signature undergo a tie-breaking scheme utilizing the differential OE effect sizes. Cells are then assigned to their cluster signature-based annotation.

### DR-GEM with annotations-in-the-loop

DR-GEM with annotations-in-the-loop utilizes signature-based cell annotations in Phase I and II of the algorithm. In this variant of DR-GEM, the weak cluster labels at the end of Phase I: ${{{{\boldsymbol{c}}}}}^{\left(1\right)}\in \{1,\ldots,K\}^{n}$, and the clusters derived in each *r* iteration are mapped to signatures, following the same procedure described in Phase IV. In this version of DR-GEM, the scores and final predictions are determined from the ensembles of signature labels in Phase II. The results of hyperparameter sensitivity analyses, DR-GEM convergence tracking, and additional data guiding default hyperparameter selection are provided in the Supplementary Materials (Supplementary Figs. 10–13).

### DR-GEM Phase II convergence and error bounds

Phase II convergence rate depends on the degree of latent class imbalance, the number of cells impacted by ambient RNA, and other sources of noise and properties of the input dataset. To tune *r* for convergence, a user can choose the auto-convergence mode where, after all cells have been sampled at least 5 times, DR-GEM assesses every *r’* iterations how much the annotations change for each final assigned label (δ_*k*_). If the fraction of assignments changed is below δ_target_ (default = 0.05) for all *K* classes (δ_*1*_,…,δ_*K*_), DR-GEM will terminate earlier than initially specified by *r* in this setting. A user can also start with a relatively small *r*, and subsequently add *r”* additional iterations without repeating the full run to reach convergence. General analytical bounds concerning DR-GEM convergence and error rates are provided in the **Supplementary Information**.

### Clustering and projections

Throughout the study clustering was performed by applying the Louvain algorithm^61^ on the data shared nearest neighbor (SNN) graph, as implemented in the Seurat R package (v5; ‘FindClusters’ function). All parameters were set to the default, except for the clustering resolution. For each dataset, a set of resolution values was tested under the standard pipeline; the resolution value that best optimized Adjusted Mutual Information (AMI) between ground truth labels and clusters was selected. Visualization on a 2-dimensional UMAP was performed with all default parameters within the Seurat R package (v5) for each dataset. Reciprocal PCA (RPCA) as implemented in the Seurat R package was used to project data to reference embeddings. The number of PCs used was determined at the beginning of each pipeline for each dataset tested using the elbow criteria.

### Clustering performance metrics

Clustering performance was evaluated based on the held-out ground truth labels using both standard metrics and analogous metrics that account for class imbalance^68^. The standard metrics used are the Adjusted Rand Index (ARI), AMI, Homogeneity Score, Completeness Score, and V-measure as implemented in the aricode (v1.0.3) R package. Since these metrics do not account for unequal class prevalence, a balanced version of each of these metrics (bARI, bAMI, bHomogeneity, bCompleteness, and bV-measure)^68^ was computed via https://github.com/hsmaan/balanced-clustering. These balance metrics reweight contributions from each ground truth class equally, mitigating the dominance of majority classes in the evaluation metrics. These metrics were computed for each test dataset to benchmark DR-GEM compared to the standard pipeline (Supplementary Data 2).

### Rare cell detection performance metrics

Rare cell detection performance was evaluated using confusion matrix-based metrics: Accuracy, Balanced Accuracy, Sensitivity (True Positive Rate), and Specificity (True Negative Rate) from the caret (v7.0.1) R package. Ground truth labels and predicted labels were converted to Rare vs. Not-rare labels for evaluation with these metrics.

### LDA, Kernel PCA, and scVI as alternatives to PCA

LDA, Kernel PCA, and scVI were applied within DR-GEM on Synthetic Dataset 1. LDA^69^ was implemented using the LDA() function in the topicmodels (v0.2.17) R package, with the number of topics set to 10. Otherwise, all default parameters were used, including Gibbs sampling for fitting the models. Kernel PCA was implemented using the kpca() function in the kernlab (v0.9.33) R package, with the kernel type set to polydot to implement a polynomial kernel. The degree was set to 2, and the number of target dimensions was set to 10; all other parameters were set to default. The VAE-based dimensionality method, scVI^110^, was run with the scvi-tools (v1.4.1) Python package as described at https://scvi-tools.org/. The number of latent dimensions was set to 10 with all other parameters set to default. These methods were used in place of PCA within the DR-GEM framework.

### Class-weighted PCA and SMOTE as alternatives to Phase II-III of DR-GEM

Both class-weighted PCA^71,111^ and SMOTE^21^ were applied after DR-GEM Phase I, using the weak clusters as input group labels on Synthetic Dataset 1. Class-weighted PCA was implemented with the svyprcomp() function within the survey (v4.4.8) R package. The reduced dimensions matrix was then used for clustering with SNN-Louvain and visualizing with UMAP. SMOTE was implemented with the themis (v1.0.3) R package to augment Synthetic Dataset 1; standard PCA as described above was applied thereafter, and the reduced dimensions matrix was then used for clustering with SNN-Louvain and visualizing with UMAP.

### Synthetic Dataset 2: ambient RNA simulation

To simulate contamination in synthetic single-cell data, a controlled signal mixing across specific cell types was added. First, gene expression matrix ${X}_{{{{\rm{Clean}}}}}$ was simulated. For each cell type A, a set of contaminating cell types ${S}_{{{{\rm{Contam}}}},A}$ is defined as well as a set of contamination weights (${w}_{A}$, between 0 and 0.6). For each cell i of cell type A, a contamination vector is defined as: ${x}_{{Contam},i,A}=w\cdot {x}_{B}$, where w is a contamination weight randomly sampled from ${w}_{A}$ and ${x}_{B}$ is a single cell gene expression vector of cell type B (where $B\in {S}_{{Contam},A}$) sampled from ${X}_{{{{\rm{Clean}}}}}$. Repeating this procedure for all cells $i\in \left\{1,\ldots n\right\}$ of all cell types $k\in \{1,\ldots,K\}$ yields a contamination matrix $({X}_{{{{\rm{Contam}}}}}\in {{\mathbb{R}}}^{n\times p})$ that is added to target cells: ${X}_{{{{\rm{Contam}}}}}+{X}_{{{{\rm{Clean}}}}}$ to yield a counts matrix. The specific parameters used to generate contamination in Synthetic Dataset 2 are provided in Supplementary Data 3. Noise (ε) and dropout (*D*) were then added, as in Synthetic Dataset 1. The resulting matrix was log-normalized and scaled for testing and analysis.

### Simulating low-quality cells and batch effects

Datasets with low-quality cells and batch effects were simulated by generating expression matrix ${X}_{{{{\rm{Clean}}}}}$ with the same parameters as Synthetic Dataset 1 and randomly selecting 20% of the cells to either (1) uniformly decrease their expression values by 2 counts and set all negative counts to zero to simulate low-quality cells, or (2) uniformly increase their expression by 2 counts. As in Synthetic Dataset 1, noise (ε) and dropout (D) were added to the resulting matrices, which were then log-normalized and scaled for testing and analysis.

### Simulating multiple rare populations

To systematically assess the impact of class imbalance with multiple rare populations, a series of synthetic datasets were simulated and assessed for RE. First, 4 discrete cell types were simulated with fixed sample sizes (*N*_1_ = 8000, *N*_2_ = 5000, *N*_3_ = 4500, *N*_4_ = 4000), then an additional *K*^(rare)^ discrete cell types, each with *N*_*K*_ = 50 were simulated for each of:

$${K}^{({rare})}\in \{2,5,10,20,100\}$$

For each *K*^*(rare)*^, 100 independent synthetic datasets were generated with *p*_1_ = 150 and *p*_2_ = 50 and a total of *p* = 200 genes using 100 unique random seeds as described in “Synthetic data generation”. Noise (ε) and dropouts (*D*) were independently simulated and applied to each dataset. Each dataset was log-normalized and scaled before application of PCA. RE (${{{\boldsymbol{e}}}}$) was computed from the reconstruction using the top 10 PCs (*m* = 10). For each simulated dataset, mean RE in each simulated cell type population was calculated and recorded.

Synthetic Dataset 3 was simulated as above with *K*^*(rare)*^ = 5. All other parameters were kept the same as for Synthetic Dataset 1.

### HGSC SMI dataset

For testing DR-GEM cell type annotations with spatial transcriptomics, HGSC SMI dataset was downloaded from Zenodo at (https://zenodo.org/records/12613839, “ST_Discovery.rds” file)^14^. First, DR-GEM was applied to 82,808 non-malignant cells profiled from 47 omentum tumors and 41 patients (Fig. 6A–C). Second, DR-GEM was applied with annotations-in-the-loop to all 491,792 cells from 100 tumor samples (53 adnexal, 47 Omentum) sampled from 58 patients (Fig. 6D**, F**). DR-GEM cell type annotations were compared to those obtained in the original paper.

To test if DR-GEM captures inter-patient and intra-tumor heterogeneity, it was applied to 185,071 malignant cells sampled from primary adnexal tumors of 43 patients (merging data from “ST_Discovery.rds” and “ST_Test2_HGSC1_malignant.rds”; Fig. 7). In all cases, the raw counts matrix was log-normalized and scaled prior to analysis.

### HGSC MERFISH dataset

HGSC MERFISH dataset was downloaded from Zenodo (https://zenodo.org/records/12613839, “ST_Validation2.rds” file)^14^. DR-GEM was applied with annotations-in-the-loop to the full dataset, spanning 425,578 cells from 4 adnexal tumors, each sampled from 4 patients (Fig. 6E**, G**). The raw counts matrix was log-normalized and scaled for subsequent testing. DR-GEM cell type annotations were compared to those obtained in the original paper.

### Mouse preoptic hypothalamus MERFISH dataset

Mouse hypothalamus MERFISH dataset was downloaded from Dryad (https://datadryad.org/dataset/doi:10.5061/dryad.8t8s248). The data from Animal 1 were used, consisting of *n* = 73,655 cells and 155 genes. From the raw counts matrix, transcripts per million (TPM) values were derived as previously described^60^. The TPM matrix was scaled before subsequent analysis and testing. Pairwise one-sided *t*-tests were applied to determine the number of DEGs between all unique pairs of the most granular cell subtype labels provided in the original paper. Cell subtypes with at least 30 DEGs (Benjamini-Hochberg (BH) False Discovery Rate (FDR) < 0.01, |log_2_FC|> 2) were defined as separate labels. The only cell subtypes that were kept separate were the endothelial subtypes, such that Endothelial 2 and Endothelial 3 cells were merged into Endothelial 2, whilst Endothelial 1 was kept separate.

### Perturb-seq data processing

A Perturb-seq dataset of a CRISPR interference screen in K562 leukemia cells was processed to TPM values from the raw counts data^10^. Only “core” control cells (*n* = 75,305), as defined in the original paper^10^ were used as control cells. 334 perturbations with strong transcriptional phenotype (# DEGs > 1000, as defined by the mean number of genes determined as significantly differentially expressed between perturbed and control cells via the Anderson-Darling test and Wilcoxon rank-sum test), at least 25 cells, and more than 30% knockdown efficiency were considered. From this set of filtered perturbations, 6 perturbations (PTPN1i, MED19i, SRRTi, MRPL24i, CASP8AP2i and NRDE2i) were selected to represent a range of transcriptional impact, biological functions, and frequency in the dataset.

### Publicly available scRNA-seq datasets

The mouse cystic fibrosis scRNA-seq dataset was downloaded from https://singlecell.broadinstitute.org/single_cell/study/SCP163/airway-epithelium (“trachea_10x_log2TPM.txt.gz” and “trachea_10x_metadata.txt” files) and utilized without any further processing. The human thymus development scRNA-seq dataset and integrated scRNA-seq atlas of thymic epithelial cells were downloaded from the Gene Expression Omnibus (GEO; “GSE212244 [https://www.ncbi.nlm.nih.gov/geo/query/acc.cgi?acc=GSE212244]”). Cells labeled as ‘Doublets’ were excluded, and the cell type and subtype labels identified in the original study were used as the ground truth. Raw PBMC scRNA-seq dataset was downloaded from the Seurat online tutorial at https://satijalab.org/seurat/articles/pbmc3k_tutorial.html. The cell type labels from the tutorial were utilized as ground truth labels. The head and neck squamous cell carcinoma scRNA-seq dataset was downloaded from GEO (“GSE103322 [https://www.ncbi.nlm.nih.gov/geo/query/acc.cgi?acc=GSE103322]”). The cell type labels as published were utilized as ground truth labels.

### Breast cancer imaging mass cytometry dataset processing

The raw breast cancer imaging mass cytometry dataset was downloaded from Zenodo (https://zenodo.org/records/3518284, “SingleCell_and_Metada.zip” file). Code from https://github.com/BodenmillerGroup/SCPathology_publication was utilized to regenerate the processed expression matrix for the “BasalTMA” dataset and the cell type labels. Cells labeled as both B and T cells were excluded as cell doublets from the dataset before subsequent testing and analysis.

### Benchmarking the standard pipeline, DR-GEM, GiniClust3, scCAD, and CIARA

The standard pipeline, DR-GEM, GiniClust3^47^, scCAD^48^, and CIARA^46^ were each applied to synthetic datasets (1 and 2), scRNA-seq (head and neck squamous cell carcinoma^58^, PBMC^63^, human thymus development^56^, mouse cystic fibrosis tracheal epithelium^57^), spatial transcriptomics (HGSC^14^, mouse preoptic hypothalamus^60^), and Perturb-seq data^10^. GiniClust3, scCAD, and CIARA were selected as three conceptually diverse rare cell detection methods published in the past 5 years. The 14 prediction tasks and 9 datasets were selected to represent diverse biological settings and different types of single-cell data.

For each method-dataset pair, performance was assessed across the metrics described in the “Rare cell detection performance metrics” and “Clustering performance metrics” sections. For evaluation of the rare cell detection metrics across the standard pipeline, DR-GEM, GiniClust3, and CIARA, ground truth labels and predicted labels were converted to the binary labels of Rare vs. Not-rare. As scCAD’s output is a set of detected rare cells when applied to each dataset, for evaluation across both rare cell detection metrics and clustering performance metrics, the ground truth labels across all datasets were converted to the binary labels of Rare vs. Not-rare for evaluation. Of note, GiniClust3 was not compatible with datasets with 200 or fewer features, precluding results for Synthetic Dataset 1, Synthetic Dataset 2, and the Mouse Preoptic Hypothalamus MERFISH dataset.

GiniClust3 was installed per the instructions described at https://github.com/rdong08/GiniClust3; clustering resolution for each dataset was selected as the resolution value that best optimized AMI between ground truth labels and clusters, the minimum Gini Index cutoff was set to 0.1, and all other input parameters were kept as default. scCAD was installed per the instructions at https://github.com/xuyp-csu/scCAD, and all parameters were kept as default values for testing. CIARA was downloaded as the CIARA (v0.1.0) R package. The clustering resolution for each dataset was selected as the resolution value that best optimized AMI between ground truth labels and clusters. The number of dimensions used for PCA within CIARA was kept consistent with those selected with DR-GEM for each dataset.

### Statistics and reproducibility

Statistical analyses were performed in R (v4.3.2). Two-group comparisons used one-sided t-tests for differential signature expression, and Wilcoxon rank-sum tests for all other values, reported with the test statistic, effect size, and 95% CIs; categorical associations used χ² tests. Exact tests, sample sizes, statistics, and P values are reported in the figures, legends, and Results. In all box plots, the center line denotes the median, box edges the 25th and 75th percentiles, and whiskers the most extreme values within 1.5 of the IQR, with outliers plotted individually.

Other than the synthetic datasets simulated here, all other datasets were previously published. Only cells that did not pass the original study quality control criteria were excluded. No cells were excluded based on method performance. No statistical method was used to predetermine sample size. The allocation to groups was predefined based on existing annotations and mappings to cell types, genetic perturbations, patients, and histological features. The Investigators were not blinded to the predefined allocation during analyses and performance evaluation. Random seeds were fixed for all stochastic procedures, including synthetic-data simulation and the class-balanced subsampling in DR-GEM Phase II to ensure reproducibility.

Reproducibility was assessed by evaluating the method across a wide range of datasets and data types, spanning scRNA-seq, spatial transcriptomics (SMI, MERFISH), and Perturb-seq, together with synthetic datasets in which the generative process, underlying distributions, and ground-truth labels are fully controlled. Rather than selecting a single synthetic data configuration, modeling and testing included a range of rare cell-type frequencies, multiple co-occurring rare cell types, and different types of data noise and confounders (i.e., dropouts, ambient RNA, low read counts, and batch effects). Across real datasets, performance was validated against a variety of held-out ground-truth information, including genetic perturbation identity, patient identity, spatial features, and published cell-type annotations rather than any single readout.

### Reporting summary

Further information on research design is available in the Nature Portfolio Reporting Summary linked to this article.

## Supplementary information

Supplementary Information

Description of Additional Supplementary Files

Supplementary Data 1-4

Reporting Summary

Transparent Peer Review file

## Source data

Source Data

## Data Availability

All synthetic and example datasets in this study are available as tabulated data and have been deposited in the “Zenodo database [https://zenodo.org/records/15285190]” (https://doi.org/10.5281/zenodo.15285189). The following data were used in this study and were downloaded from publicly accessible databases as described hereinafter. The HGSC data were downloaded from Zenodo under accession code “12613839 [https://zenodo.org/records/12613839]” (https://doi.org/10.5281/zenodo.8327117)^14^. The mouse preoptic hypothalamus MERFISH data were downloaded from Dryad under accession code “https://doi.org/10.5061/dryad.8t8s248 [https://datadryad.org/dataset/doi:10.5061/dryad.8t8s248]”. The K562 Perturb-seq dataset is available via “Figshare [https://doi.org/10.25452/figshare.plus.20029387]“^10^. The mouse cystic fibrosis scRNA-seq data were downloaded from the Single Cell Portal under accession code “SCP163 [https://singlecell.broadinstitute.org/single_cell/study/SCP163/airway-epithelium]”. The human thymus development scRNA-seq dataset and integrated scRNA-seq atlas of thymic epithelial cells were downloaded from GEO under accession code “GSE212244 [https://www.ncbi.nlm.nih.gov/geo/query/acc.cgi?acc=GSE212244]”. The PBMC scRNA-seq data were downloaded from the “Seurat online tutorial [https://satijalab.org/seurat/articles/pbmc3k_tutorial.html]”. The head and neck squamous cell carcinoma scRNA-seq data were downloaded from the GEO database under accession code “GSE103322 [https://www.ncbi.nlm.nih.gov/geo/query/acc.cgi?acc=GSE103322]”. The breast cancer imaging mass cytometry data were downloaded from “Zenodo [https://zenodo.org/records/3518284]” (https://doi.org/10.5281/zenodo.4607374). Any additional requests for information can be directed to, and will be fulfilled by, the corresponding author. Source data are provided with this paper.

## Code availability

DR-GEM as an end-to-end tool is available as an R package with tutorials available on GitHub (https://github.com/Jerby-Lab/drgem; https://doi.org/10.6084/m9.figshare.32938931)^112^. Python-based code for running DRO in PCA as a Linear Autoencoder and Tilted Fair PCA is also available on GitHub (https://github.com/Jerby-Lab/fair-lin-ae).
