# Supplementary material for: DR-GEM enables self-supervised machine learning for single-cell embeddings and annotations
Source: Nat Commun. 2026 Sep 24;17:11088. doi: 10.1038/s41467-026-77908-z (PMC13696685; doi:10.1038/s41467-026-77908-z)
Supplement: Supplementary file 1 [file 41467_2026_77908_MOESM1_ESM.pdf]

Supplementary Information

SUPPLEMENTARY NOTE

DR-GEM runtime, memory usage, and practical strategies for resource-limited settings

DR-GEM runtime scales primarily with the number of Phase II iterations (*r*) and the average number of cells per iteration (**Supplementary Data 4**). As datasets increase in scale in merged cohorts and with high-resolution platforms, repeated dimensionality reduction and label-matching steps may create practical bottlenecks.

For large-scale datasets, it is recommended to run Phase II with single runs executed in parallel or in batches with an auto-convergence mode between batches. The analytical and empirical convergence rates are provided to help users make informed decisions about the number of iterations needed (**Methods**). For exceptionally large datasets or limited resources, Phase II can be applied to a balanced subsample of the data. More specifically, apply Phase I to the entire data to obtain the initial embedding and weak cluster annotation for all cells. Next, based on the initial embedding from Phase I, obtain a balanced subsample of the data to be provided as input for Phase II, thus reducing the chance that rare or underrepresented cells will be subsampled. Lastly, once the DR-GEM reference embedding is obtained for this balanced subset in Phases II and III, the remaining cells can be projected onto it in Phase III.

Additional practical strategies for reducing memory and runtime include limiting the runs to highly variable features (or any other feature selection method most relevant to study system) based on the balanced embedding of Phase I, adjusting the per-cluster sampling cap, preserving sparse matrix representations where possible, and, as outlined above, parallelization of Phase II, and using the auto-convergence criterion to stop Phase II once consensus annotations stabilize.

DR-GEM Phase II is currently implemented with parallelized CPU execution across balanced resampling runs and can be further parallelized on high-performance computing clusters. Future implementations may incorporate GPU-accelerated implementations of common dimensionality reduction methods, graph construction, and clustering operations to improve throughput while preserving the underlying DR-GEM algorithm. These GPU-based optimizations can be implemented alongside future developments such as incorporating online learning for Phase II of DR-GEM as described in the Discussion.

The use of DR-GEM with annotations-in-the-loop

DR-GEM annotations-in-the-loop mode can be used to further orient the model to biological variation of interest. In annotations-in-the-loop mode, the signature dictionary can impact the output, and it is thus recommended to match the granularity of the signatures to the annotation level of interest (e.g., cell type, subtype, state). Importantly, as the criterion for assigning a cluster to a signature is relatively stringent, the risk that rare cells will be forced to predefined categories that do not adequately represent them is low. A cluster that does not meet the assignment criteria for any of the signatures is labeled as "Undetermined". Cells that are repeatedly labeled as “Undetermined” get a high confidence score and are hence likely to be included in the final reference map. Cross-dataset validation and other diagnostics such as scGraph[1] and other tools can be used to investigate DR-GEM output.

High Reconstruction Error (RE) due to feature underrepresentation

In accordance with prior studies[2], there is evidence that in datasets collected with targeted panels, high RE can also reflect and be used to identify feature imbalance. In the imaging mass cytometry data spanning 35 proteins, highly abundant stromal cells exhibited disproportionately elevated RE, in accordance with the relatively low number of stromal proteins included in the panel (**Supplementary Fig. 2D**). Importantly, due to weak cluster assignments, DR-GEM will still down sample such high RE abundant cells, resulting in a decrease in rare cell type RE (**Supplementary Fig. 11E**) and improved detection (B cells: 88% vs. 0% with the standard pipeline).

Analytically demonstrating DR-GEM Phase II convergence and error bounds

Given the input data, let S include all the data subsets consistent with the sampling procedure of Phase II, such that the total size of S is N. Let c_{ij} be the annotation of cell i in a subset $j \in S$. The target annotation of cell i is defined as the cell's most frequent annotation across all N subsets:

$$c_i^* = argmax_k \sum_{j=1}^{N} 1[c_{ij} = k] \tag{1}$$

As shown below, this is the annotation that Phase II converges to as a function of γ_i, defined as

$$\gamma_i = \sum_{j=1}^{N} \frac{1[c_{ij} = c_i^*]}{N} \tag{2}$$

The confidence score s_i is the estimate of γ_i based on $S_r \subset S$, where $|S_r| = r$.

Let $\hat{c_i}$ be the annotation that Phase II converges to within r iterations. The probability of correct labeling across r iterations thus follows a binomial distribution:

$$\Pr(\hat{c_i} = c_i^* |\, s_i \geq s_i^*) = \frac{\Pr(\hat{c_i} = c_i^*, s_i \geq s_i^*)}{\Pr(s_i \geq s_i^*)} \geq \Pr(\hat{c_i} = c_i^*, s_i \geq s_i^*) = \sum_{n=\lceil r * s_i^* \rceil}^{r} \binom{r}{n} \gamma_i^n (1-\gamma_i)^{r-n} \tag{3}$$

Thus, within 1, 3, 5, 7, 9, 17, 29, 67, and 269 iterations the probability of an error is ≤ 5% for cells with γ_i = 0.95, 0.9, 0.85, 0.8, 0.75, 0.7, 0.65, 0.6, and 0.55, respectively (**Supplementary Fig. 10A**).

The Odds Ratio (OR) between the probability of correct to incorrect labeling cell i increases as a function of s_i.

$$OR_i = \frac{\Pr(\hat{c_i} = c_i^* |\, s_i > \hat{s_i} > 0.5)}{\Pr(\hat{c_i} \neq c_i^* |\, s_i > \hat{s_i} > 0.5)} = \frac{\Pr(\hat{c_i} = c_i^*, s_i > \hat{s_i} > 0.5)}{\Pr(\hat{c_i} \neq c_i^*, s_i > \hat{s_i} > 0.5)} \geq \frac{\sum_{n=\lceil r * \hat{s_i} \rceil}^{r} \binom{r}{n} \gamma_i^n (1-\gamma_i)^{r-n}}{\sum_{n=\lceil r * \hat{s_i} \rceil}^{r} \binom{r}{n} (1-\gamma_i)^n {\gamma_i}^{r-n}} \tag{4}$$

Thus, the cells used for the reference map in Phase III are less likely to be mislabeled.

The Hoeffding's inequality for Bernoulli random variables provides an exponential bound on the probability that s_i deviates from γ_i by a specific margin t, as a function of r (**Supplementary Fig. 10B**).

$$\Pr(s_i - \gamma_i \geq t) \leq e^{-2rt^2} \tag{5}$$

$$\Pr(\gamma_i > s_i - t) \geq 1 - e^{-2rt^2} \tag{6}$$

If selecting cells with $s_i \geq 0.95$ for the reference maps, as done here, it is estimated that within 17 iterations per cell, 74.3% of the cells selected for the reference map will have a $\gamma_i > 0.75$ and thus correctly annotated with an error rate ≤ 5%, such that considering all the reference cells the total error rate is ≤ 15%.

$$\Pr(\gamma_i \geq 0.75 \mid s_i \geq 0.95, r \geq 17) \geq 1 - e^{-34 * 0.2^2} = 0.743 \tag{7}$$

SUPPLEMENTARY FIGURES

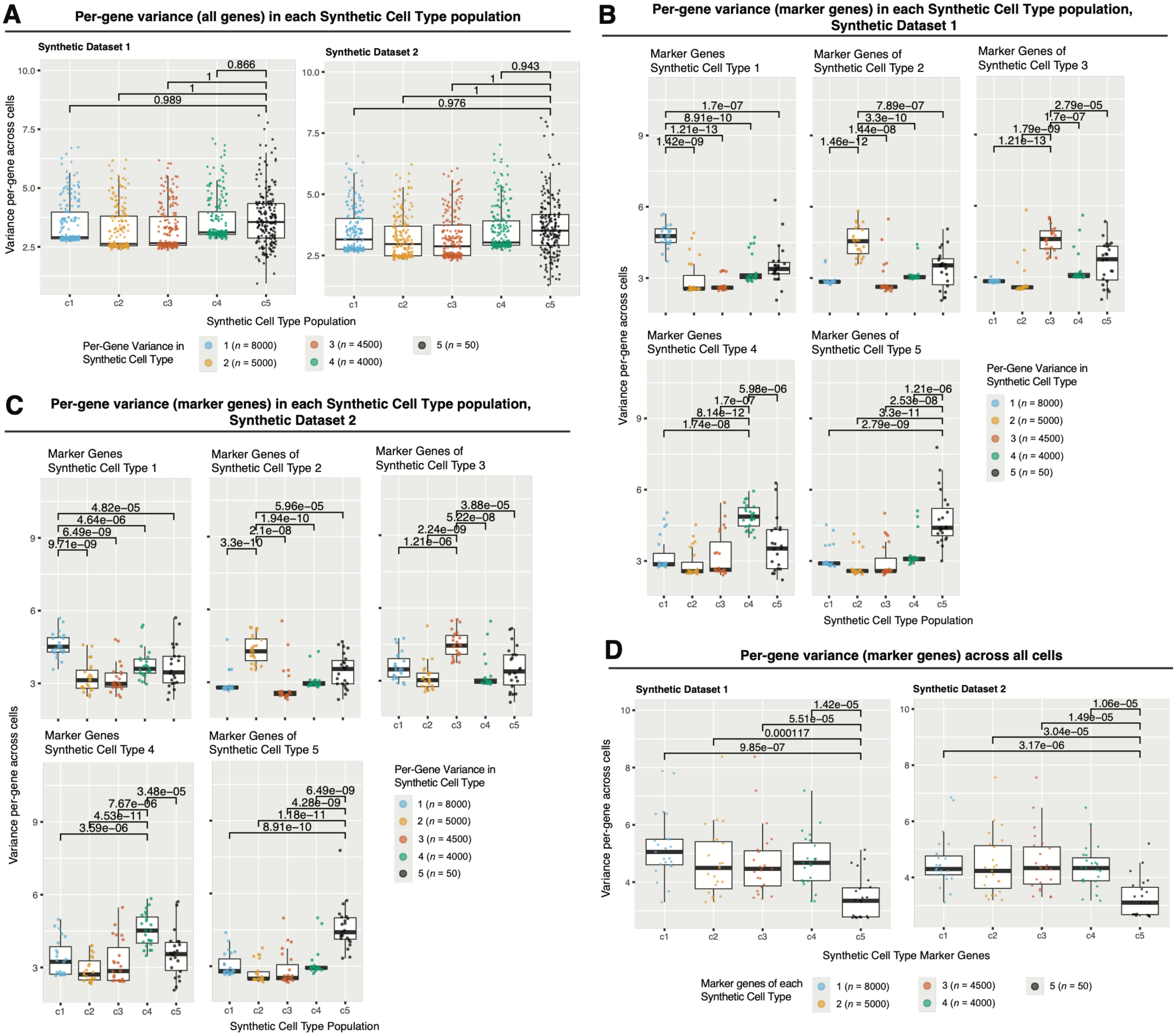

Supplementary Figure 1. Cell type abundance impacts marker gene variance in synthetic datasets. (A) Variance (y-axis) of all genes within each cell-type population (x-axis) in Synthetic Dataset 1 (left) and 2 (right). All p-values were derived from a one-sided Wilcoxon rank sum test. **(B-C)** Variance (y-axis) of cell type marker genes (subtitle) within each cell type population (x-axis) in Synthetic Dataset 1 (B) and 2 (C). **(D)** Variance (y-axis) of cell type marker genes (x-axis) in the entire Synthetic Dataset 1 (left) and Dataset 2 (right). All p-values between indicated populations were derived from a one-sided Wilcoxon rank-sum test with Benjamini-Hochberg (BH) multiple-hypothesis correction. Boxplots middle line: median; box edges: 25th and 75th percentiles; whiskers: most extreme points that do not exceed ± interquartile range (IQR) × 1.5; further outliers are marked individually as black points.

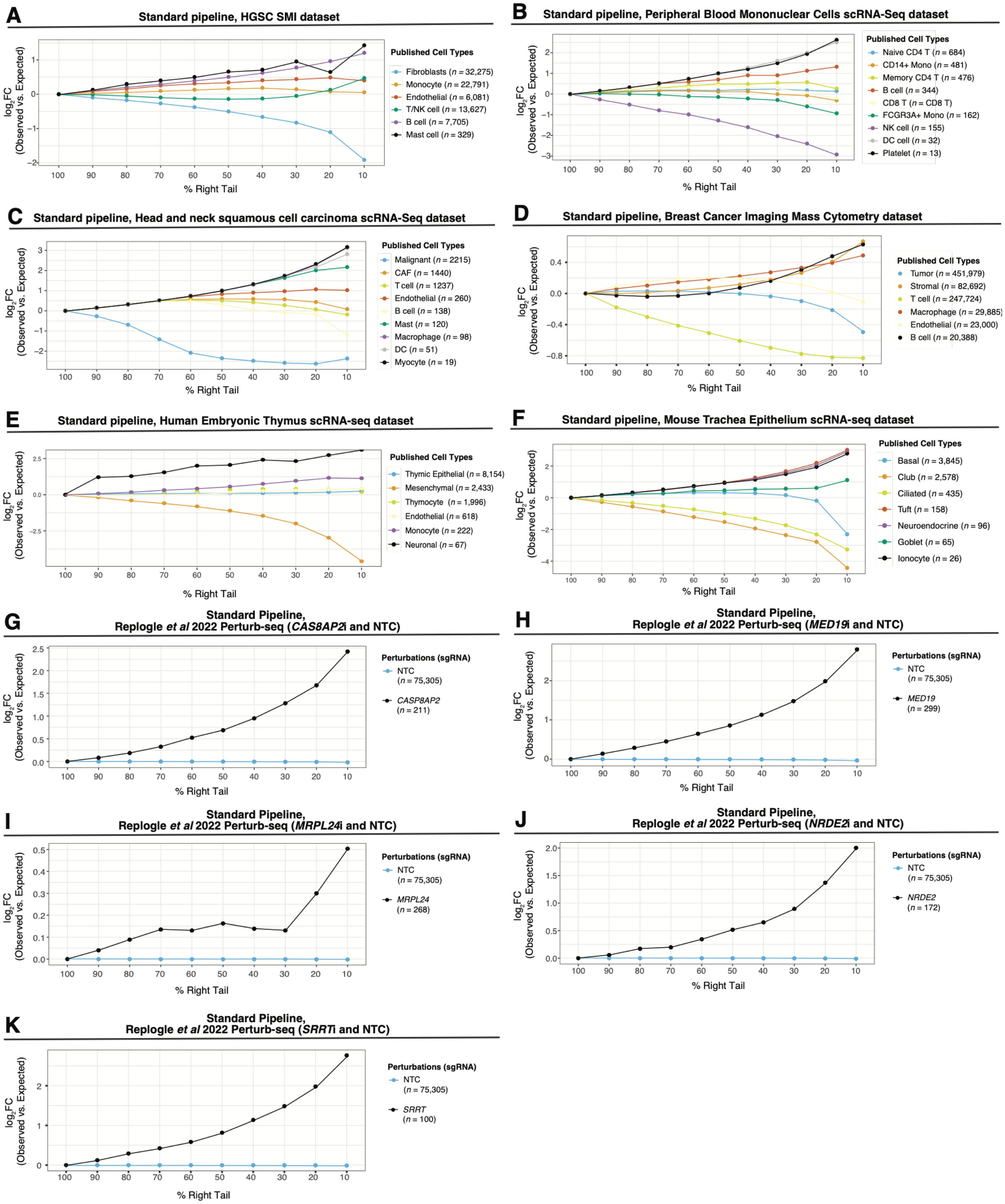

Supplementary Figure 2. Cells from minority/rare latent classes are enriched among the cells with the highest RE in standard embeddings. Log_2FC (y-axis) of observed vs. expected percentage of each class (color) in the right-tail (of varying sizes, x-axis) of the RE distribution under standard PCA in the following datasets: **(A)** spatial transcriptomics (SMI) profiling of high grade serous tubo-ovarian carcinoma (HGSC)[3], shown for metastatic HGSC sampled from the omentum, **(B)** scRNA-seq of peripheral blood mononuclear cells (PBMC) from a healthy donor[4], **(C)** scRNA-seq of head and neck squamous cell carcinoma tumors[5], **(D)** Imaging Mass Cytometry (IMC) profiling of breast cancer tumors[6], **(E)** scRNA-seq of the developing human thymus[7], **(F)** scRNA-seq of the tracheal epithelium from mouse models of cystic fibrosis and control mice[8], **(G-K)** Perturb-seq[9] profiling of K562 cells subject to CRISPR interference (CRISPRi), showing cells expressing non-targeting control or *CASP8AP2* (G), *MED19* (H), *MRPL24* (I), *NRDE2* (J), and *SRRT* (K) single guide RNAs (sgRNAs). Source data are provided as a Source Data file.

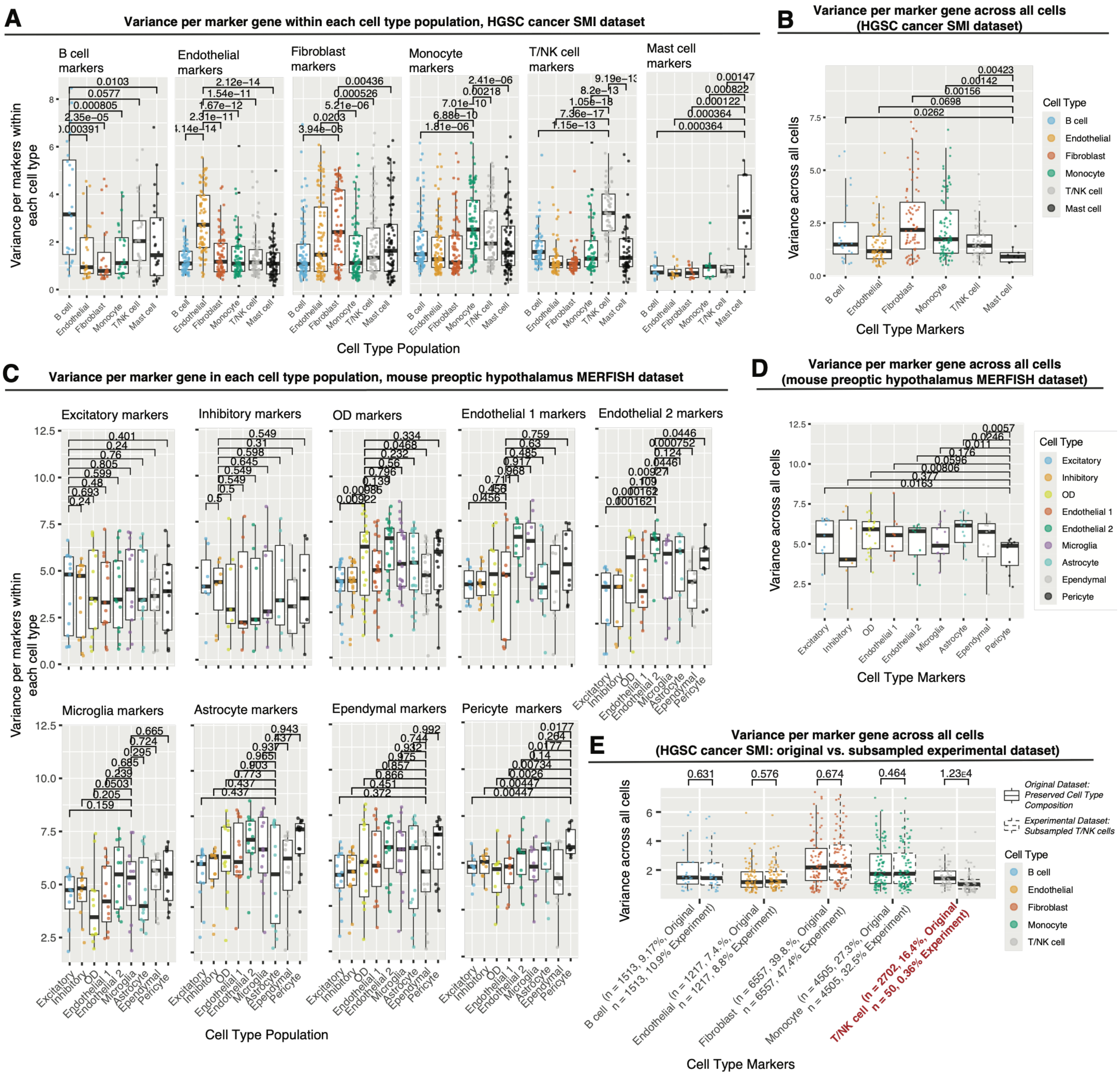

Supplementary Figure 3. Cell type abundance impacts marker gene variance in real-world datasets with latent class imbalance. (A-B) Tubo-ovarian cancer SMI dataset: **(A)** Variance (*y*-axis) of cell type marker genes (subtitle) within each non-malignant cell type population (*x*-axis); **(B)** variance (*y*-axis) of cell type marker genes (x-axis) across all non-malignant cells in the dataset. **(C-D)** Mouse preoptic hypothalamus MERFISH dataset: **(C)** Variance (*y*-axis) of cell type marker genes (subtitle) within each cell type population (*x*-axis); **(D)** variance (*y*-axis) of cell type marker genes (x-axis) across all cells in the dataset. **(E)** Gene variance (y-axis) for different cell-type marker genes (colors) in the original (solid line) and subsampled (dashed line) HGSC SMI dataset, with T/NK cells subsampled to 0.36% of the dataset. All *p*-values between indicated populations were derived from a one-sided Wilcoxon rank-sum test with BH multiple-hypothesis correction. Boxplots middle line: median; box edges: 25th and 75th percentiles; whiskers: most extreme points that do not exceed ± IQR × 1.5; further outliers are marked individually as black points.

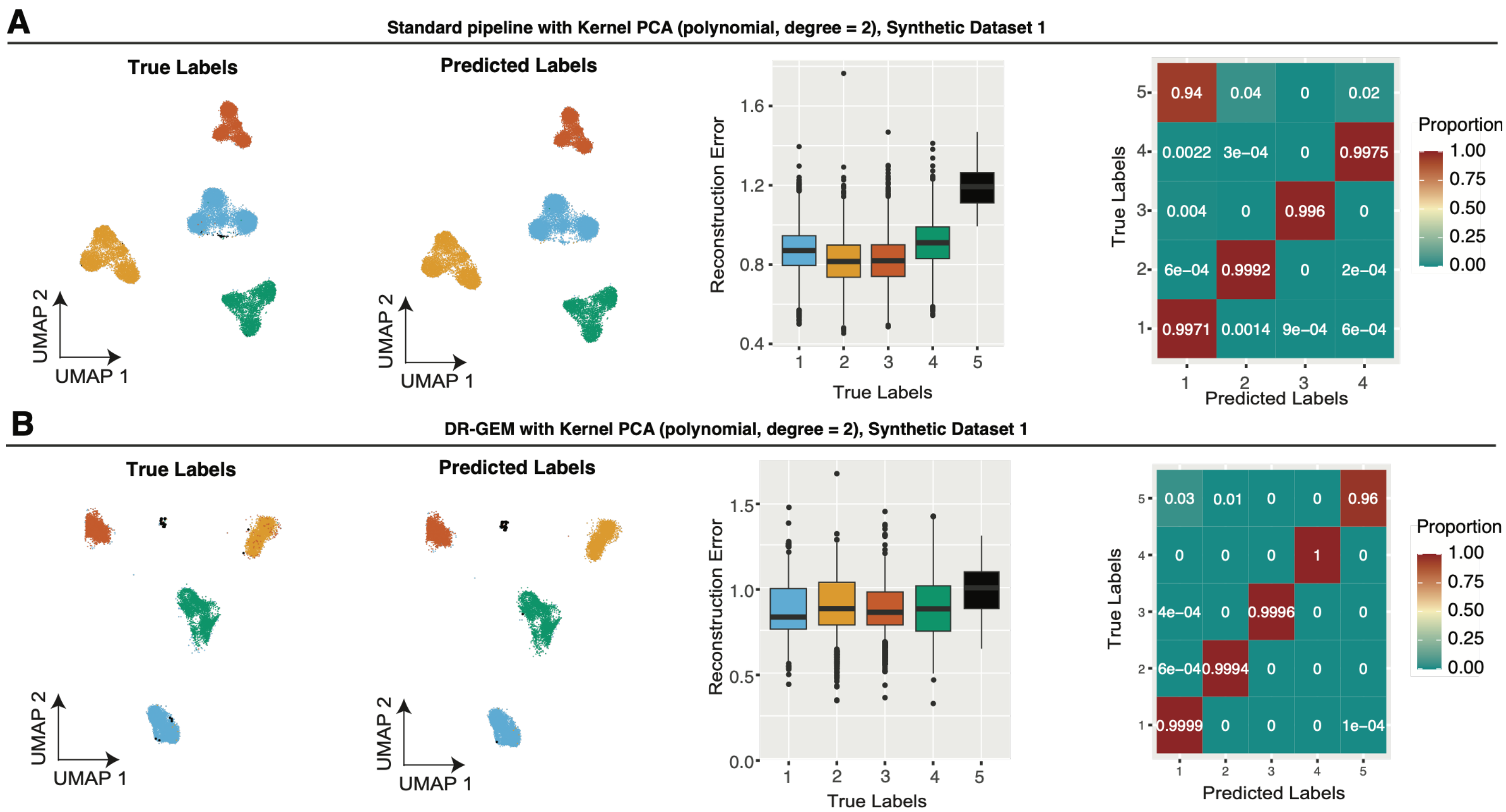

Supplementary Figure 4. DR-GEM outperforms the standard pipeline when both are applied with Kernel PCA. Performance of **(A)** the standard pipeline and **(B)** DR-GEM on Synthetic Dataset 1 with kernel PCA. **(A-B)** UMAP visualization of samples colored by ground truth synthetic cell type labels (left) and predicted cluster labels (middle left), boxplots of RE (*y*-axis) in each ground truth synthetic cell type (*x*-axis, middle right), and proportion of predicted cell type (*x*-axis) in each of the true cell type populations (*y*-axis; right). Label colors the same as in **Fig. 1.** Boxplots middle line: median; box edges: 25th and 75th percentiles; whiskers: most extreme points that do not exceed ± IQR × 1.5; further outliers are marked individually as black points.

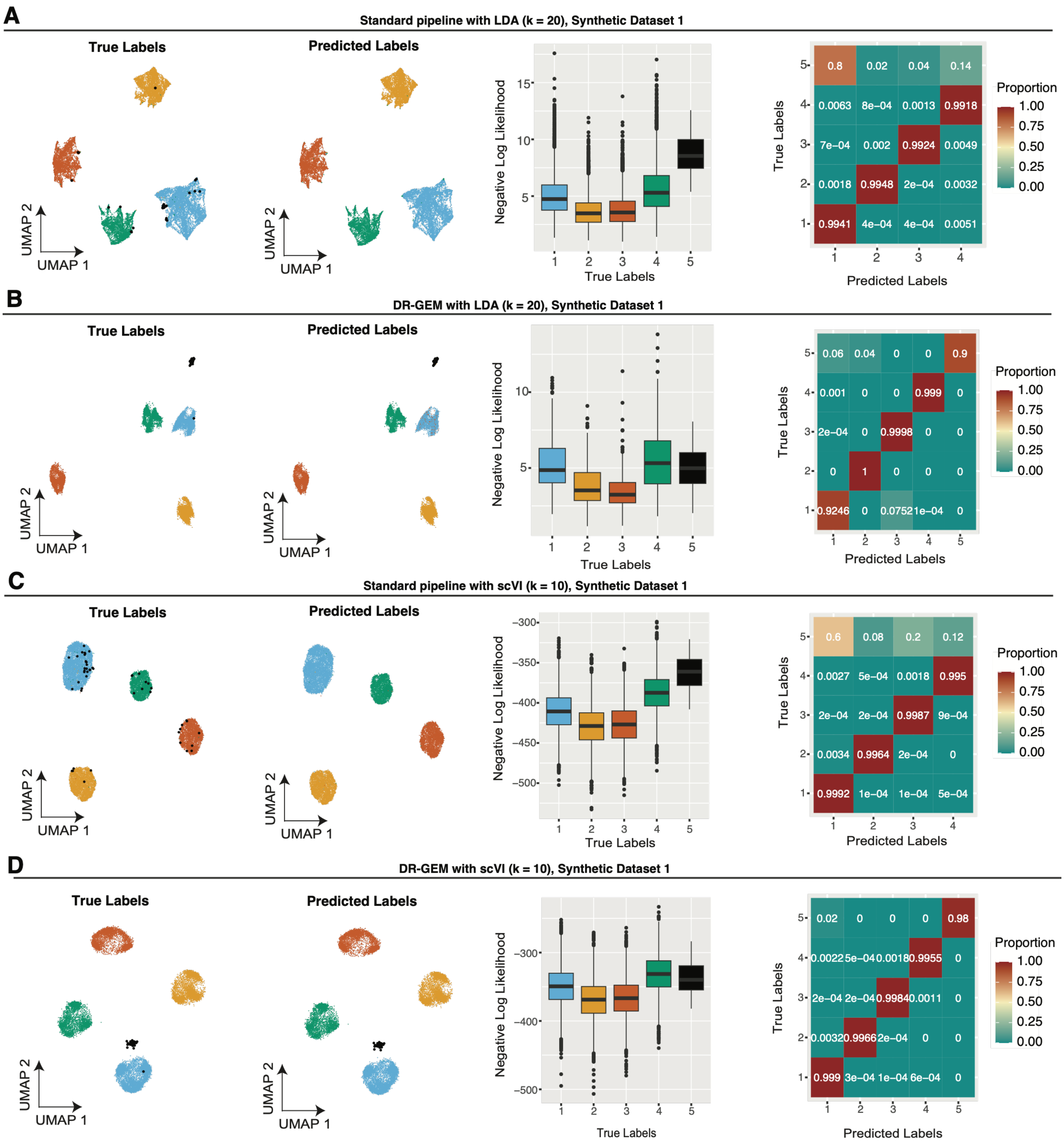

Supplementary Figure 5. DR-GEM outperforms the standard pipeline when both are applied with LDA or with scVI. Performance of **(A,C)** standard pipeline and **(B,D)** DR-GEM (**Methods**) on Synthetic Dataset 1 with **(A-B)** Latent Dirichlet Allocation (LDA) and **(C-D)** single cell Variational Inference (scVI): **(A-D)** UMAP visualization of samples colored by ground truth synthetic cell type labels (left) and predicted cluster labels (middle left), boxplots of RE (*y*-axis) in each ground truth synthetic cell type (*x*-axis, middle right), and proportion of predicted cell type (*x*-axis) in each of the true cell type populations (*y*-axis; right). Label colors are the same as in **Fig. 1.** Boxplot middle line: median; box edges: 25th and 75th percentiles; whiskers: most extreme points that do not exceed ± IQR × 1.5; further outliers are marked individually as black points.

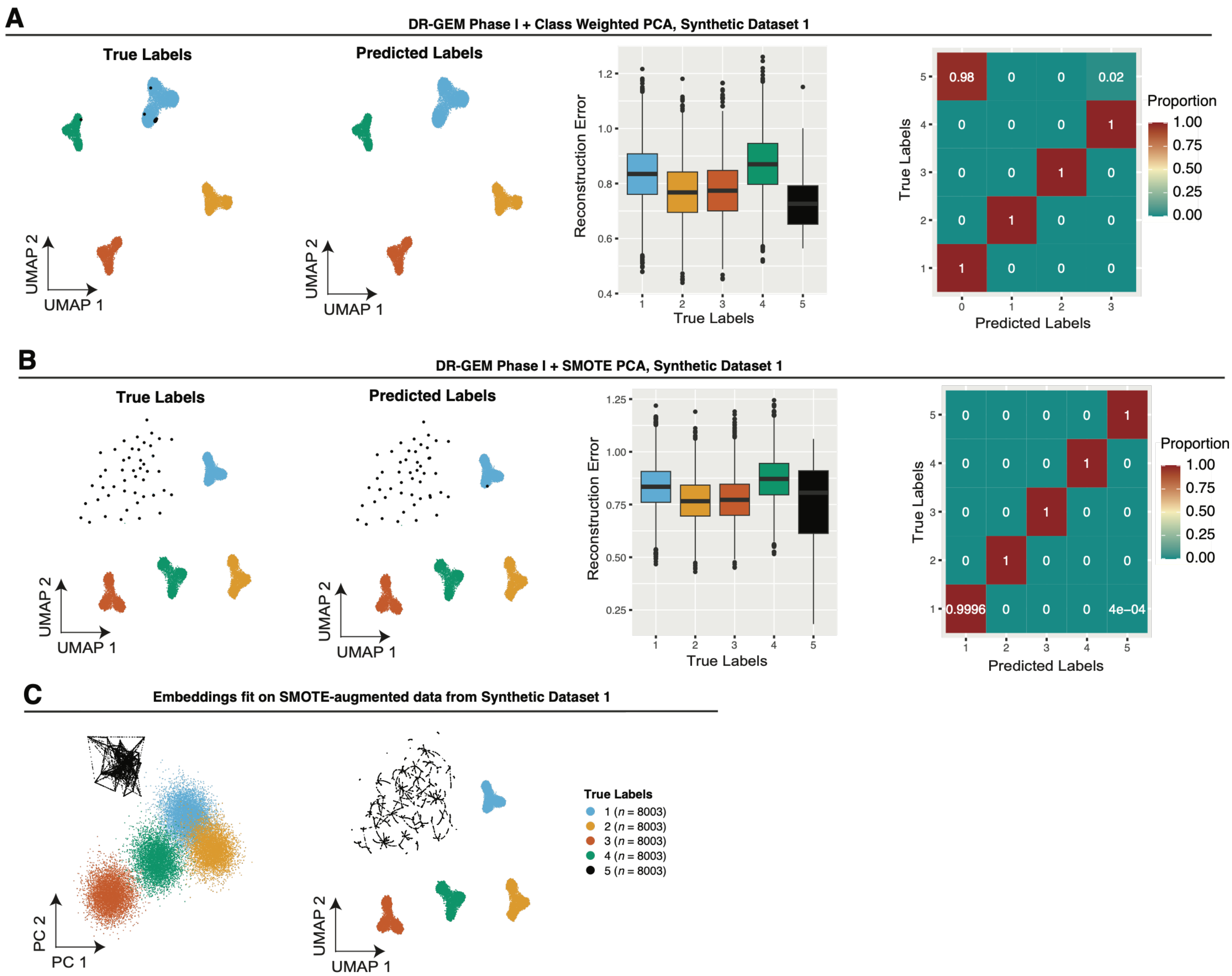

Supplementary Figure 6. Testing alternatives to DR-GEM Phase II-III. (A-B) UMAP visualization of samples colored by ground truth synthetic cell type labels (left) and predicted cluster labels (middle left), boxplots of RE (y-axis) in each ground truth synthetic cell type (x-axis, middle right), and proportion of predicted cell type (x-axis) in each of the true cell type populations (y-axis; right) using **(A)** Class weighted PCA in place of DR-GEM Phase II-III, and **(B)** SMOTE followed by PCA in place of DR-GEM Phase II-III. **(C)** Single cells from the SMOTE-augmented data of Synthetic Dataset 1, visualized in the top 2 PCs (left) and UMAP dimensions (right), colored by ground-truth synthetic cell types. Boxplots middle line: median; box edges: 25th and 75th percentiles; whiskers: most extreme points that do not exceed ± IQR × 1.5; further outliers are marked individually as black points.

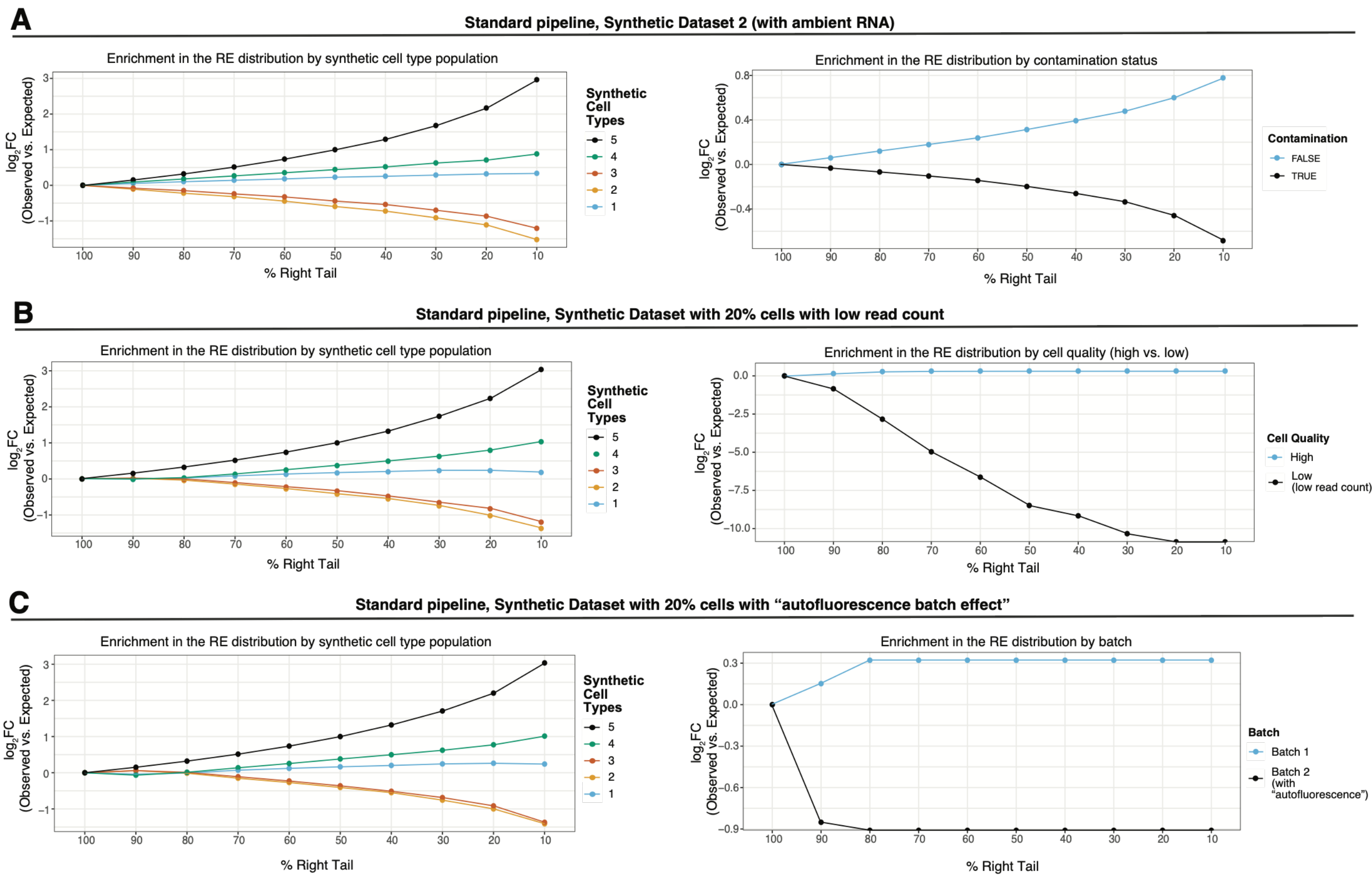

Supplementary Figure 7. High RE marks rare cell types and is not associated with noise in simulated datasets. (A-C) Log$_2$FC (y-axis) of observed vs. expected percentage of each cell subset (color) in the right-tail (of varying sizes, x-axis) colored by synthetic cell type (left) and technical noise (right), shown for: **(A)** Synthetic Dataset 2, **(B)** Synthetic dataset with latent class imbalance and 20% of cells with "low read counts", and **(C)** Synthetic data with latent class imbalance and 20% of cells impacted by "batch effect" **(Methods)**. Source data are provided as a Source Data file.

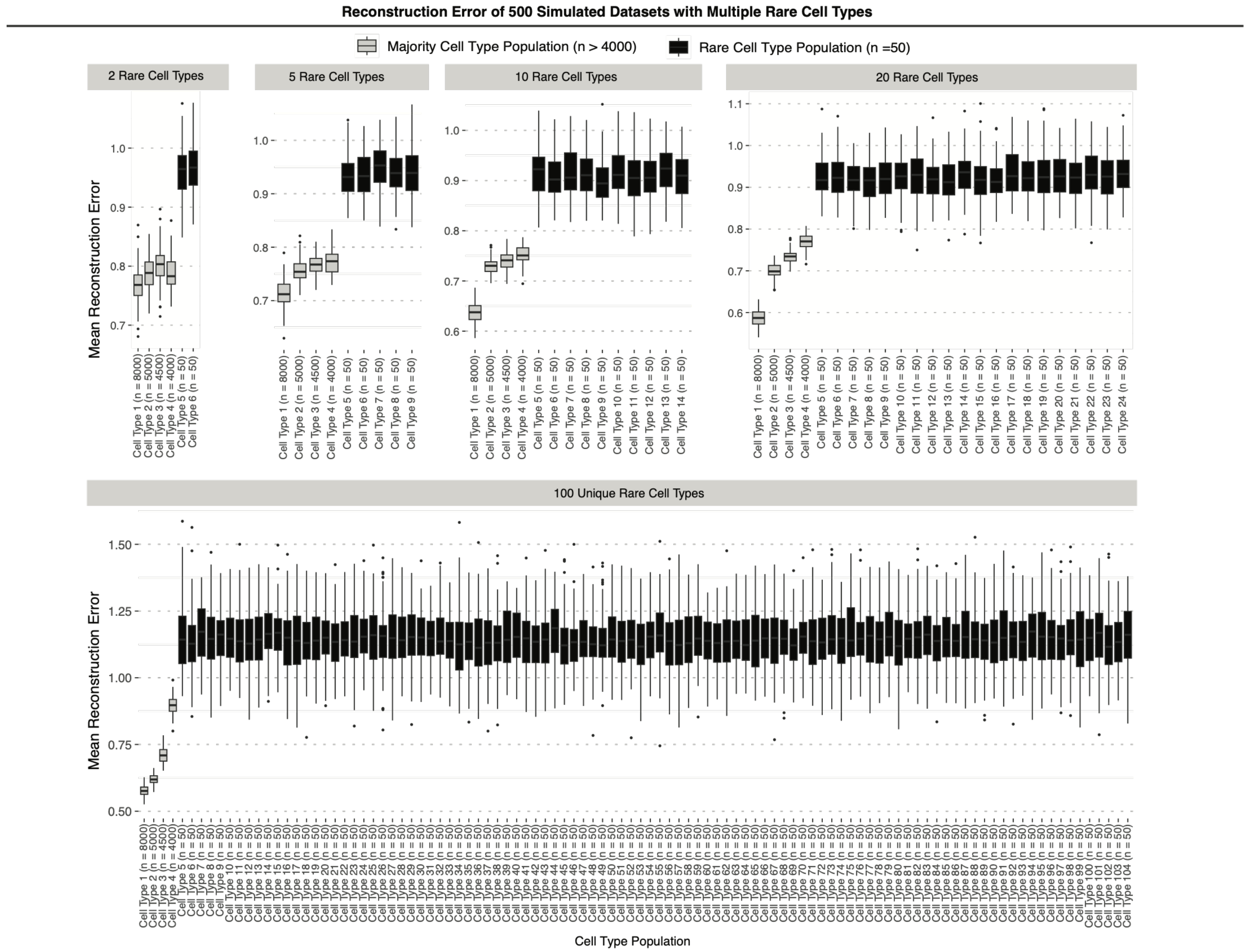

Supplementary Figure 8. High RE values mark rare cell types in datasets simulated with multiple minority/rare cell types. Mean RE (*y*-axis) of each synthetic cell type population (*x*-axis) across 100 datasets simulated from each category, that is, containing (top, from left to right) 2, 5, 10, 20, and (bottom) 100 minority/rare cell types. Boxplots: middle line: median; box edges: 25th and 75th percentiles; whiskers: most extreme points that do not exceed ± IQR × 1.5; further outliers are marked individually as black points. Source data are provided as a Source Data file.

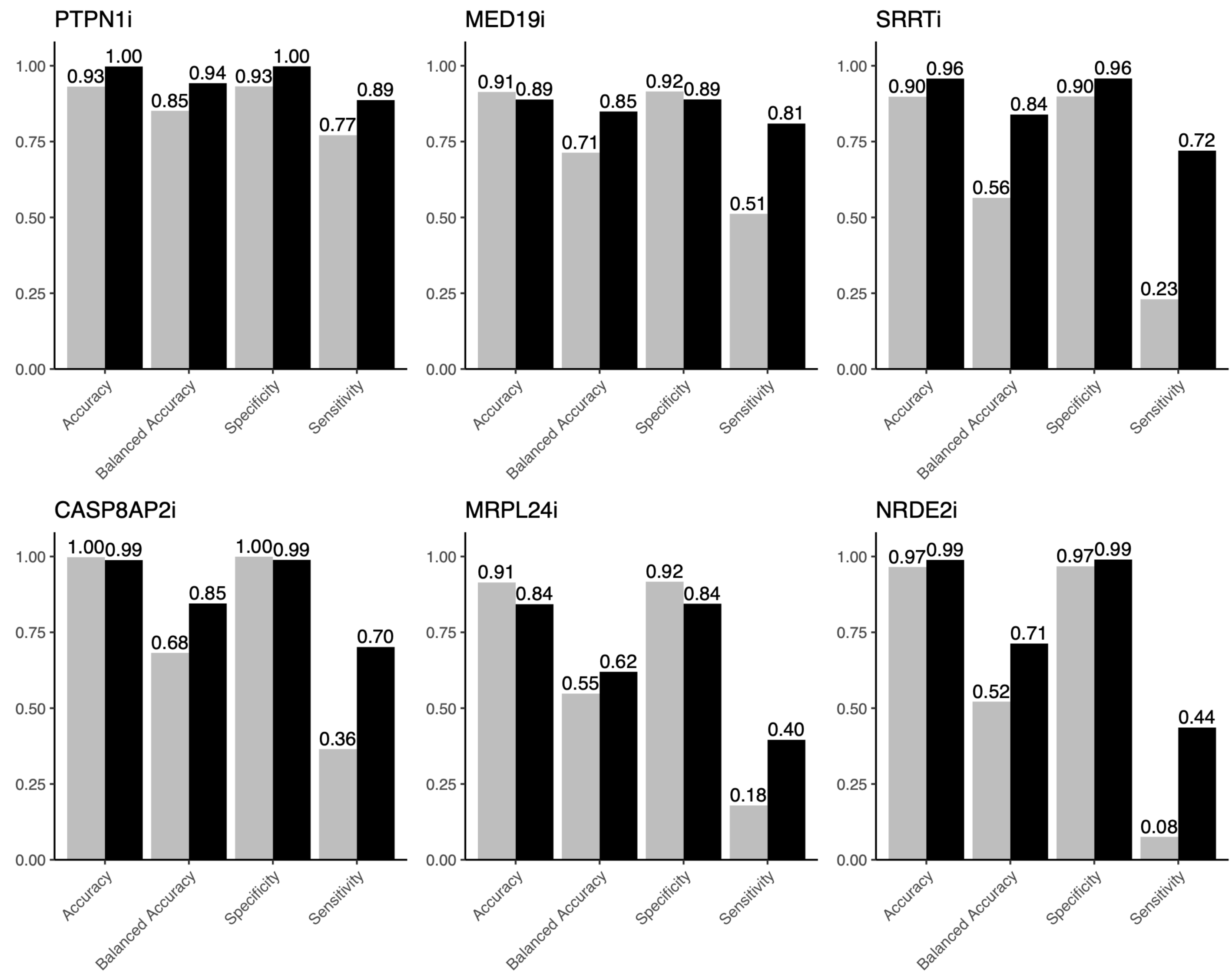

Supplementary Figure 9. Further performance metrics of the Standard pipeline vs. DR-GEM on Perturb-seq data. DR-GEM (black) vs. standard pipeline (grey) performance (*y*-axis) based on standard classification metrics (*x*-axis) in distinguishing PTPN1i (top left), MED19i (top middle), SRRTi (top right), MRPL24i (bottom left), CASP8AP2i (bottom middle), and NRDE2i (bottom right) vs. NTC cells. Abbreviations: CASP8AP2i, MED19i, MRPL24i, NRDE2i, SRRTi, CRISPRi targeting *CASP8AP2, MED19, MRPL24, NRDE2*, and *SRRT*, respectively; NTC, non-targeting control.

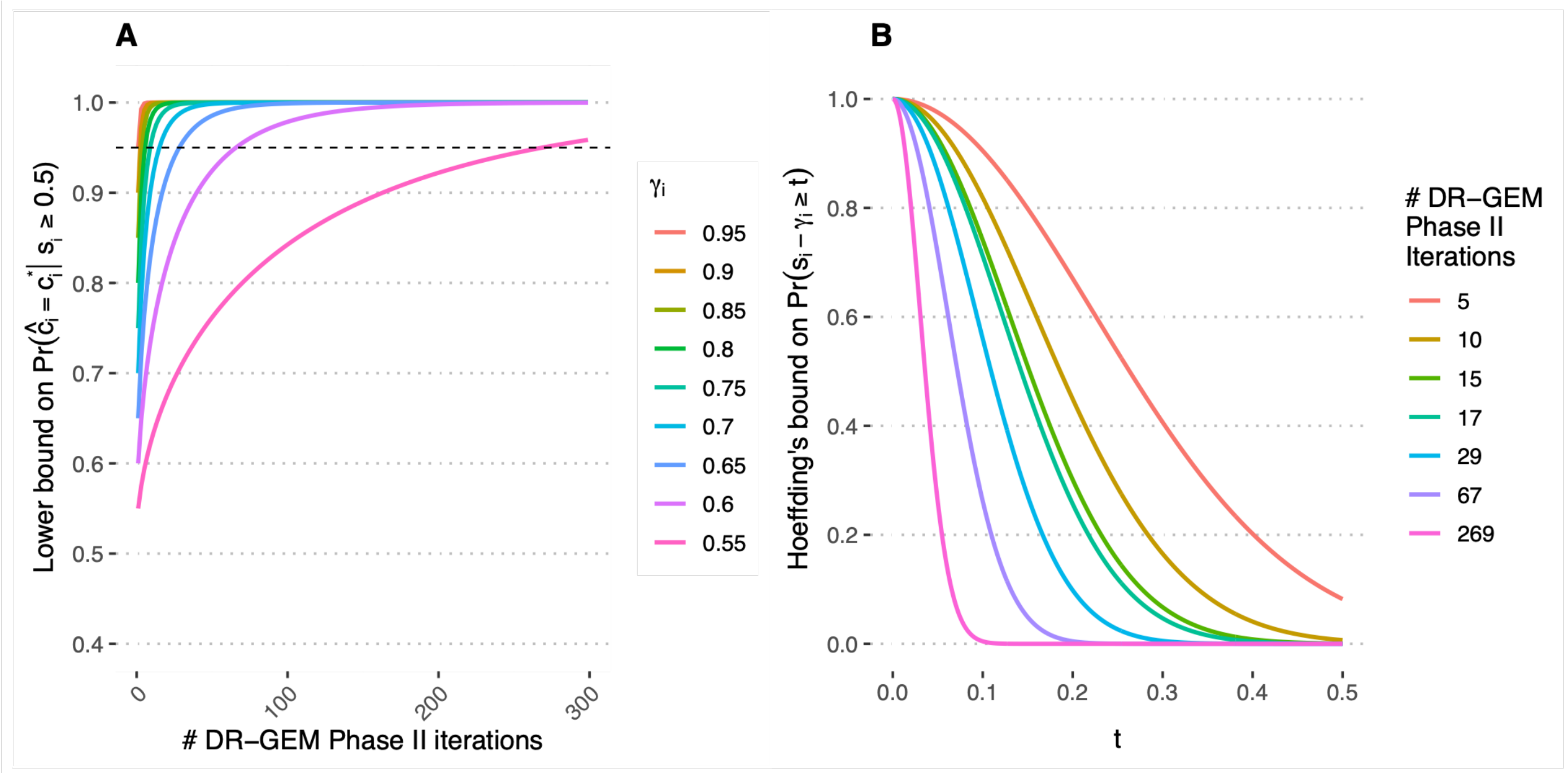

Supplementary Figure 10. Phase II convergence rate and error bounds. (A) The lower bound on the probability that a cell with $s_i^* = 0.5$ is labeled correctly (y-axis) as a function of the number of iterations run (x-axis), with each line representing a different γ_i value [0.55, 0.95]. **(B)** Hoeffding's inequality bounds of the probability (y-axis) that the confidence score s_i deviates from the γ_i by t (x-axis) as a function of the number of Phase II iterations (r, lines).

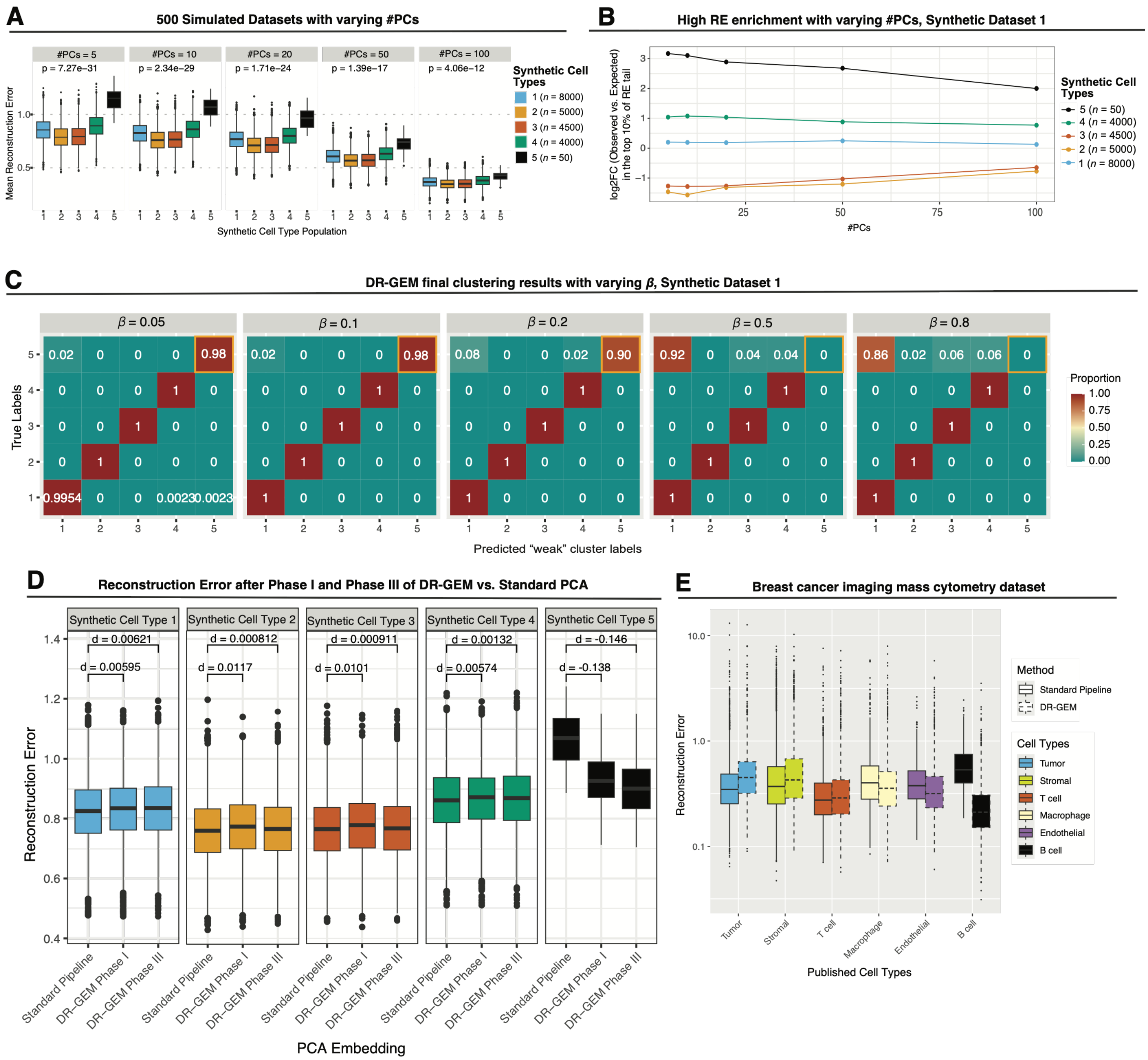

Supplementary Figure 11. Sensitivity analysis of DR-GEM hyperparameters. **(A)** Mean RE (*y*-axis) within each synthetic cell type population (*x*-axis) across 500 simulated datasets with different numbers of PCs used for reconstruction (columns). *p*-values from a one-sided Wilcoxon rank-sum test comparing Cell Type 5 (minority/rare cell type) and Cell Type 1 (majority cell type). **(B-D)** Synthetic Dataset 1 analyses: **(B)** Log_2FC (*y*-axis) of observed vs. expected percentage of each cell type (color) in the top 10% of the RE distribution with varying number of PCs used for reconstruction (*x*-axis) in Synthetic Dataset 1. **(C)** Proportion of DR-GEM's predicted cell type (*x*-axis) in each of the true cell type populations (*y*-axis) in Synthetic Dataset 1 with different β values (columns). **(D)** DR-GEM decreases RE values of minority/rare cells with minimal impact on the RE of abundant cell types. RE (*y*-axis) after: (i) standard PCA, (ii) Phase I of DR-GEM, and (iii) Phase III of DR-GEM (*x*-axis) in each cell type population (subtitles). *d* denotes the difference in mean RE from the two different embeddings fit on Synthetic Dataset 1. **(E)** RE (y-axis) across published cell types (x-axis) in the breast cancer imaging mass cytometry dataset with the standard pipeline (solid lines) vs. DR-GEM (dotted lines). (A, D-E) Boxplot middle line: median; box edges: 25th and 75th percentiles; whiskers: most extreme points that do not exceed ± IQR × 1.5; further outliers are marked individually as black points. Source data are provided as a Source Data file.

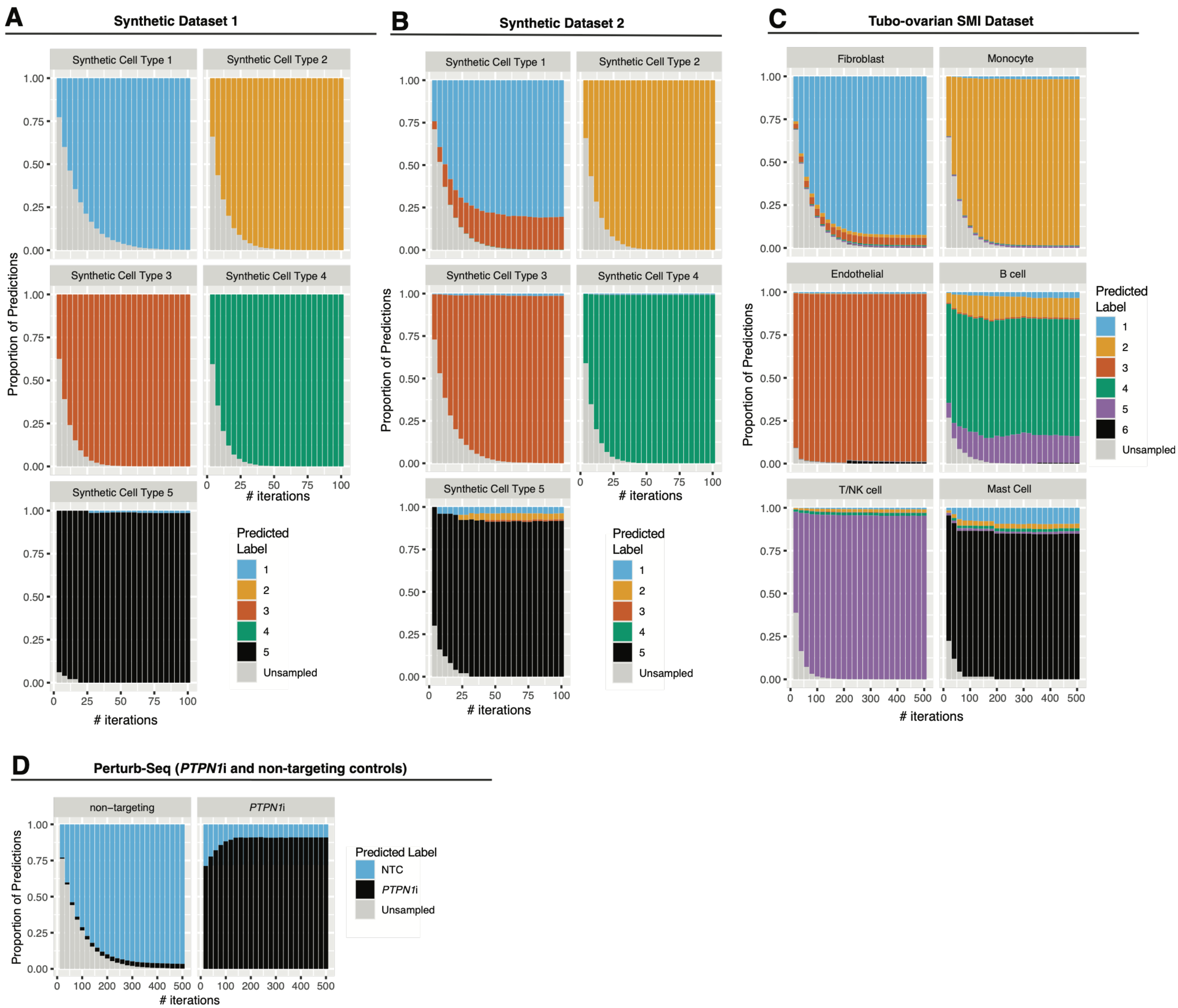

Supplementary Figure 12. Proportion of predictions across iterations in Phase II of DR-GEM. Proportion of predictions (y-axis) across iterations (x-axis) of Phase II of DR-GEM in **(A)** Synthetic Dataset 1, **(B)** Synthetic Dataset 2, **(C)** Tubo-ovarian cancer SMI dataset, and **(D)** Perturb-seq (*PTPN1*i vs. non-targeting). Subplots stratified by ground-truth labels; bar colors represent predicted labels in Phase II of DR-GEM. Source data are provided as a Source Data file.

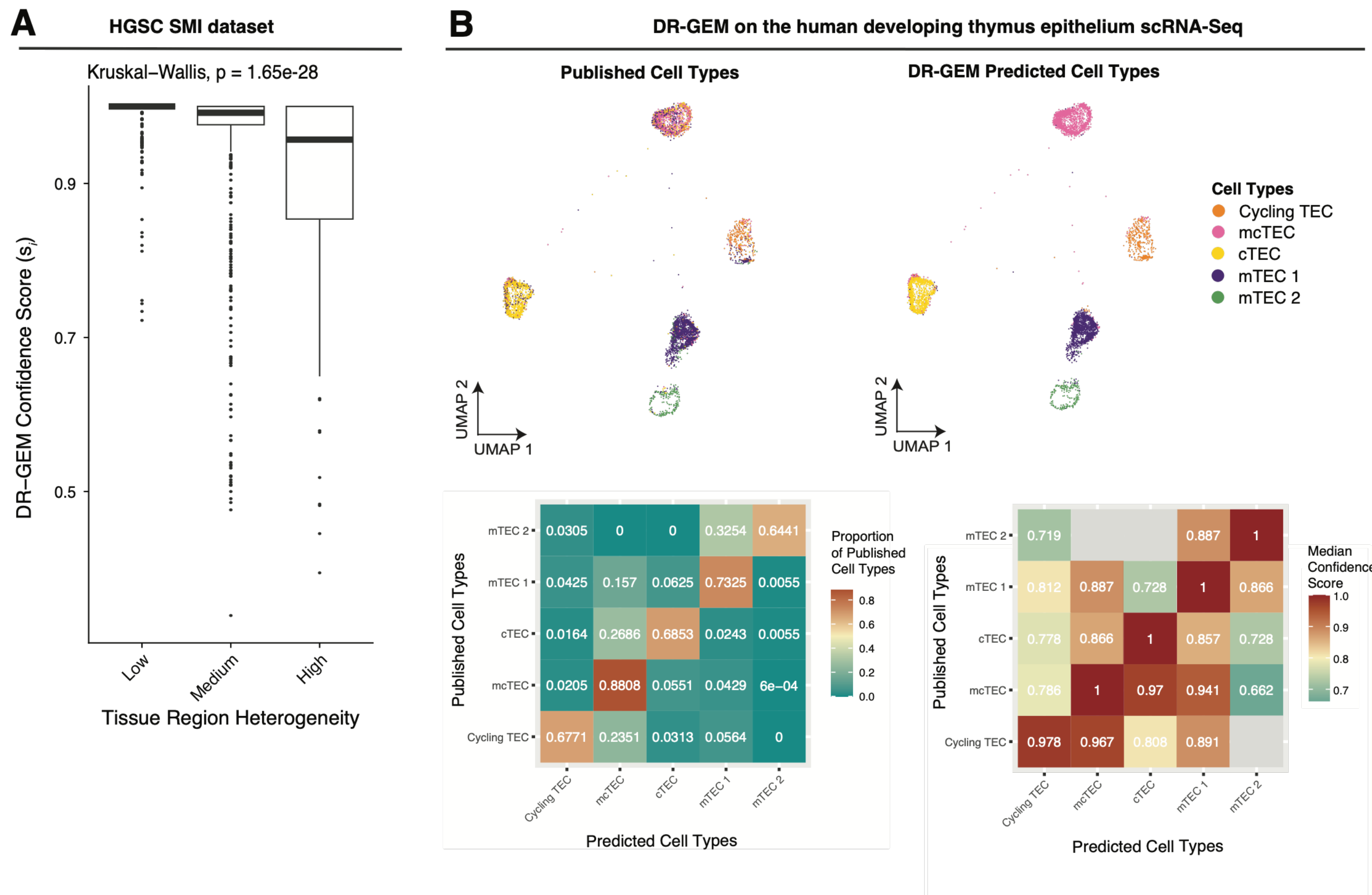

Supplementary Figure 13. DR-GEM confidence scores are associated with cell state transitions and ambient RNA. (A) DR-GEM applied to scRNA-seq dataset of the human developing thymus. Top: UMAP visualization of cells colored by published (left) and DR-GEM (right) predicted cell types. Bottom left: proportion of predicted cell type (*x*-axis) in each of the published cell type populations (*y*-axis). Bottom right: median DR-GEM confidence scores (s_i) across published cell types (*y*-axis) stratified by DR-GEM's predicted cell types (*x*-axis). **(B)** DR-GEM applied to HGSC spatial transcriptomics data: Confidence scores (s_i, *y*-axis) as a function of tissue heterogeneity (Shannon index of heterogeneity of cell types sampled within a 30um radius around each cell; High = top quartile of values, Low = bottom quartile of values, Middle = between bottom and top quartile of values, *x*-axis). Boxplot middle line: median; box edges: 25th and 75th percentiles; whiskers: most extreme points that do not exceed ± IQR × 1.5; further outliers are marked individually as black points. *p*-value derived from the Kruskal-Wallis test of difference in medians.

SUPPLEMENTARY REFERENCES

1. Wang, H., Leskovec, J. & Regev, A. Limitations of cell embedding metrics assessed using drifting islands. *Nature Biotechnology* **44**, 574–577 (2026).
2. Khani, F. & Liang, P. Feature noise induces loss discrepancy across groups. in *International conference on machine learning* 5209–5219 (PMLR, 2020).
3. Yeh, C. Y. *et al.* Mapping spatial organization and genetic cell-state regulators to target immune evasion in ovarian cancer. *Nat Immunol* **25**, 1943–1958 (2024).
4. Analysis, visualization, and integration of Visium HD spatial datasets with Seurat. https://satijalab.org/seurat/articles/pbmc3k_tutorial.
5. Puram, S. V. *et al.* Single-Cell Transcriptomic Analysis of Primary and Metastatic Tumor Ecosystems in Head and Neck Cancer. *Cell* **171**, 1611-1624.e24 (2017).
6. Jackson, H. W. *et al.* The single-cell pathology landscape of breast cancer. *Nature* **578**, 615–620 (2020).
7. Mohammed, A. *et al.* Distinct type I and II interferon responses direct cortical and medullary thymic epithelial cell development. *Science Immunology* **10**, eado4720 (2025).
8. Montoro, D. T. *et al.* A revised airway epithelial hierarchy includes CFTR-expressing ionocytes. *Nature* **560**, 319–324 (2018).
9. Replogle, J. M. *et al.* Mapping information-rich genotype-phenotype landscapes with genome-scale Perturb-seq. *Cell* **185**, 2559-2575.e28 (2022).
10. Maan, H. *et al.* Characterizing the impacts of dataset imbalance on single-cell data integration. *Nature Biotechnology* **42**, 1899–1908 (2024).
