# Supplementary material for: DR-GEM enables self-supervised machine learning for single-cell embeddings and annotations
Source: Nat Commun. 2026 Sep 24;17:11088. doi: 10.1038/s41467-026-77908-z (PMC13696685; doi:10.1038/s41467-026-77908-z)
Supplement: Supplementary file 2 [file 41467_2026_77908_MOESM2_ESM.pdf]

Description of Additiona1 Supplementary Files

Supplementary Data 1: Expression variance of marker genes of Cell Type 5 in Synthetic Dataset 1 and Synthetic Dataset 2 before DR-GEM and after Phase II DR-GEM.

Supplementary Data 2: Summary of rare cell detection metrics (**Methods**) and (balanced[10] and standard) clustering performance indices (**Methods**) across each dataset tested in this study. Orange shading in column B denotes the top-performing method across all metrics. Green shading denotes the top score for each metric for each dataset.

Supplementary Data 3: Parameter details for ambient RNA simulations in Synthetic Dataset 2. Column A lists all target cell types (cell type A); column B lists all sets of "donor" cell types for each target cell type A; and column C lists all sets of contamination weights $\boldsymbol{w}_A$.

Supplementary Data 4: DR-GEM computational resource usage for representative datasets.
