# Supplementary material for: DR-GEM enables self-supervised machine learning for single-cell embeddings and annotations
Source: Nat Commun. 2026 Sep 24;17:11088. doi: 10.1038/s41467-026-77908-z (PMC13696685; doi:10.1038/s41467-026-77908-z)
Supplement: Supplementary file 3 [file 41467_2026_77908_MOESM4_ESM.pdf]

nature portfolio

Corresponding author(s): Livnat Jerby

Last updated by author(s): Sep 2, 2026

Reporting Summary

Nature Portfolio wishes to improve the reproducibility of the work that we publish. This form provides structure for consistency and transparency in reporting. For further information on Nature Portfolio policies, see our Editorial Policies and the Editorial Policy Checklist.

Statistics

For all statistical analyses, confirm that the following items are present in the figure legend, table legend, main text, or Methods section.

n/a	Confirmed	
☐	☒	The exact sample size (n) for each experimental group/condition, given as a discrete number and unit of measurement
☐	☒	A statement on whether measurements were taken from distinct samples or whether the same sample was measured repeatedly
☐	☒	The statistical test(s) used AND whether they are one- or two-sided<br>*Only common tests should be described solely by name; describe more complex techniques in the Methods section.*
☐	☒	A description of all covariates tested
☐	☒	A description of any assumptions or corrections, such as tests of normality and adjustment for multiple comparisons
☐	☒	A full description of the statistical parameters including central tendency (e.g. means) or other basic estimates (e.g. regression coefficient) AND variation (e.g. standard deviation) or associated estimates of uncertainty (e.g. confidence intervals)
☐	☒	For null hypothesis testing, the test statistic (e.g. F, t, r) with confidence intervals, effect sizes, degrees of freedom and P value noted<br>*Give P values as exact values whenever suitable.*
☒	☐	For Bayesian analysis, information on the choice of priors and Markov chain Monte Carlo settings
☒	☐	For hierarchical and complex designs, identification of the appropriate level for tests and full reporting of outcomes
☐	☒	Estimates of effect sizes (e.g. Cohen's d, Pearson's r), indicating how they were calculated

Our web collection on statistics for biologists contains articles on many of the points above.

Software and code

Policy information about availability of computer code

Data collection	Data utilized and tested in this study were synthetically generated or already publicly available. Detailed information describing the source of each dataset used in the study is provided in the Methods.
Data analysis	DR-GEM as an end-to-end tool is available as an R package with tutorials available on GitHub (https://github.com/Jerby-Lab/drgem; doi:10.6084/m9.figshare.32938931). Python-based code for running DRO in PCA as a Linear Autoencoder and Tilted Fair PCA is also available on GitHub (https://github.com/Jerby-Lab/fair-lin-ae).<br><br>All data analyses were conducted using open-source software, including R (v4.3.2); Python (v3.9); Seurat (v5); survey (v4.4.8); themis (v1.0.3); topicmodels (v0.2.17); kernlab (v0.9.33); lpSolve; aricode (v1.0.3); caret (v7.0.1); CIARA (v0.1.0); GiniClust3; scCAD; scvi-tools (v1.4.1)/scVI; PyTorch.

For manuscripts utilizing custom algorithms or software that are central to the research but not yet described in published literature, software must be made available to editors and reviewers. We strongly encourage code deposition in a community repository (e.g. GitHub). See the Nature Portfolio guidelines for submitting code & software for further information.

Data

Policy information about availability of data

All manuscripts must include a data availability statement. This statement should provide the following information, where applicable:

- Accession codes, unique identifiers, or web links for publicly available datasets
- A description of any restrictions on data availability
- For clinical datasets or third party data, please ensure that the statement adheres to our policy

All synthetic and example datasets in this study are available as tabulated data and have been deposited in the Zenodo database (https://zenodo.org/records/15285190, doi:10.5281/zenodo.15285189). The following data were used in this study and were downloaded from publicly accessible databases as described hereinafter. The HGSC data was downloaded from Zenodo under accession code 12613839 (https://zenodo.org/records/12613839; doi:10.5281/zenodo.8327117)14. The mouse preoptic hypothalamus MERFISH data were downloaded from Dryad under accession code doi:10.5061/dryad.8t8s248 (https://datadryad.org/dataset/doi:10.5061/dryad.8t8s248). The K562 Perturb-seq dataset is available via Figshare https://doi.org/10.25452/figshare.plus.2002938710. The mouse cystic fibrosis scRNA-seq data were downloaded from the Single Cell Portal under accession code SCP163 (https://singlecell.broadinstitute.org/single_cell/study/SCP163/airway-epithelium). The human thymus development scRNA-seq dataset and integrated scRNA-seq atlas of thymic epithelial cells were downloaded from GEO under accession code GSE212244 (https://www.ncbi.nlm.nih.gov/geo/query/acc.cgi?acc=GSE212244). The PBMC scRNA-seq data were downloaded from the Seurat online tutorial at https://satijalab.org/seurat/articles/pbmc3k_tutorial.html. The head and neck squamous cell carcinoma scRNA-seq data were downloaded from the GEO database under accession code GSE103322 (https://www.ncbi.nlm.nih.gov/geo/query/acc.cgi?acc=GSE103322). The breast cancer imaging mass cytometry data were downloaded from Zenodo (https://zenodo.org/records/3518284; doi:10.5281/zenodo.4607374). Source data are provided with this paper.

Research involving human participants, their data, or biological material

Policy information about studies with human participants or human data. See also policy information about sex, gender (identity/presentation), and sexual orientation and race, ethnicity and racism.

Reporting on sex and gender	There was no sex- or gender-based analysis performed as a part of this study. Information about sex and gender was not included in any of the analyses.<br><br>The study generated synthetic data. All other datasets used were previously published. Information about the source is provided in the Data Availability section.
Reporting on race, ethnicity, or other socially relevant groupings	Information about race, ethnicity, or other socially relevant groupings was not included in any of the analyses.
Population characteristics	Patient-level information and features were not included in any of the analyses.
Recruitment	n/a
Ethics oversight	n/a

Note that full information on the approval of the study protocol must also be provided in the manuscript.

Field-specific reporting

Please select the one below that is the best fit for your research. If you are not sure, read the appropriate sections before making your selection.

☒ Life sciences ☐ Behavioural & social sciences ☐ Ecological, evolutionary & environmental sciences

For a reference copy of the document with all sections, see nature.com/documents/nr-reporting-summary-flat.pdf

Life sciences study design

All studies must disclose on these points even when the disclosure is negative.

Sample size	DR-GEM was tested on an array of synthetic and real-world datasets across a range of sample sizes and class-imbalance settings to test its performance in direct comparison to other methods. No statistical method was used to predetermine sample size.
Data exclusions	Only cells that did not pass the original study quality control criteria were excluded. No cells were excluded based on method performance. In certain analyses only a subset of the data was used as described in the main text and Method sections.
Replication	DR-GEM was tested across different types of data (i.e., simulated, scRNA-Seq, SMI, MERFISH, and Perturb-Seq) from different studies to rigorously test its performance and generalizability.
Randomization	The allocation to groups was predefined based on existing annotations and mappings to cell types, genetic perturbations, patients, and histological features. Random seeds were fixed for all stochastic procedures, including synthetic-data simulation and the class-balanced subsampling in DR-GEM Phase II to ensure reproducibility.

Blinding	The Investigators were not blinded to the predefined allocation during analyses and performance evaluation.

Reporting for specific materials, systems and methods

We require information from authors about some types of materials, experimental systems and methods used in many studies. Here, indicate whether each material, system or method listed is relevant to your study. If you are not sure if a list item applies to your research, read the appropriate section before selecting a response.

Materials & experimental systems

n/a	Involved in the study
☒	☐ Antibodies
☒	☐ Eukaryotic cell lines
☒	☐ Palaeontology and archaeology
☒	☐ Animals and other organisms
☒	☐ Clinical data
☒	☐ Dual use research of concern
☒	☐ Plants

Methods

n/a	Involved in the study
☒	☐ ChIP-seq
☒	☐ Flow cytometry
☒	☐ MRI-based neuroimaging

Plants

Seed stocks	n/a
Novel plant genotypes	n/a
Authentication	n/a
