# Supplementary material for: DR-GEM enables self-supervised machine learning for single-cell embeddings and annotations
Source: Nat Commun. 2026 Sep 24;17:11088. doi: 10.1038/s41467-026-77908-z (PMC13696685; doi:10.1038/s41467-026-77908-z)
Supplement: Supplementary file 4 [file 41467_2026_77908_MOESM5_ESM.pdf]

nature portfolio

Peer Review File

DR-GEM enables self-supervised machine learning for single-cell embeddings and annotations

Corresponding Author: Dr Livnat Jerby

This file contains all reviewer reports in order by version, followed by all author rebuttals in order by version.

Version 0:

Reviewer comments:

Reviewer #1

(Remarks to the Author)
The authors propose a novel dimensionality reduction method for single-cell analysis, addressing two key limitations of traditional approaches: (1) the distortion of rare cell type distributions in low-dimensional embeddings, and (2) sensitivity to technical noise (e.g., empty droplets). While the identified problem is compelling and biologically relevant, the methodological novelty and rigor require further clarification.

Major comments:
1. The author's question about rare cell types being mixed up is interesting. This may occur because rare cell types often exhibit lower gene expression variance compared to the majority cell types. PCA, being a linear projection method, selects axes based on feature variance. If rare cell types are poorly represented after reduction, it suggests their distinguishing gene expression signals are overshadowed by the high-variance signals of the majority cell types. I suggest authors to examine this point.
2. LDA, Kernel PCA, Class-weighted PCA and SMOTE+PCA should be tested.
3. If the method simply repeats subsampling and reprojection from scratch, how does it enhance embedding quality? The iterative process alone does not inherently guarantee better representations unless there's a mechanism that refines embeddings. It seems the core idea of this method is to select some cells for obtaining better projection matrix. Why is it a self-supervised learning techinque?
4. The high-confidence cells are selected via ensemble strategies. How does this specifically address noise from empty droplets? Since ensemble methods aggregate multiple predictions, they may reduce stochastic errors, but a clearer justification for droplet noise suppression would be helpful.
5. In Phase I, the projection matrix is derived from high reconstruction error (RE) cells. Could this bias the embeddings for other cells? If Phase II operates on these potentially skewed embeddings, how does it correct or improve them?
6. Are there mathematical guarantees (e.g., convergence properties, error bounds) ensuring that Phase II refines embeddings rather than amplifying initial biases?
Minor comments:
1. P_1 (instead of P1) should be subindexed for proper mathematical notation in Results section 1.
2. How is the second embedding learned? Is PCA applied again, using the same projection matrix to obtain embeddings for all cells?
3. In Phase II, the description states: "DR-GEM then fits r (user-specified) learners that yield r sets of predictions fit on r balanced subsamples of the data."
a) Does this mean clustering is performed *r* times on subsamples?
b) The phrasing "fit learners and make predictions" could be misinterpreted as training a supervised classifier using cell labels. If unsupervised, the wording should be clarified.
4. I do not think that direct using cluster annotation to supervise cell embeddings is a good choice (Phase IV). This may hurt other potential biological signals in the cells, as indicated by (https://www.nature.com/articles/s41587-025-02702-z). Author should discuss the potential limitation when using this optional phase.

(Remarks on code availability)
Code availability:
Code is well written and provide a comprehensive tutorial. However, the link (https://github.com/Jerby-Lab/fair-lin-ae) is invalid.

Reviewer #2

(Remarks to the Author)
I co-reviewed this manuscript with one of the reviewers who provided the listed reports. This is part of the Nature Communications initiative to facilitate training in peer review and to provide appropriate recognition for Early Career Researchers who co-review manuscripts.

(Remarks on code availability)
Code is well written and provide a comprehensive tutorial. However, the link (https://github.com/Jerby-Lab/fair-lin-ae) is invalid.

Reviewer #3

(Remarks to the Author)
This manuscript introduces a self-supervised meta-algorithm named DR-GEM, designed to address the limitations of standard dimensionality reduction and clustering methods in identifying rare cell types and distinguishing biological signals from technical noise in single-cell and spatial omics analysis. The authors propose that existing methods tend to overfit to the dominant patterns in the data, thereby ignoring or incorrectly embedding rare cell populations. DR-GEM addresses this issue through an innovative multi-phase framework that integrates hard-example identification based on reconstruction error, balanced ensemble learning, and the construction of a high-confidence reference atlas. The authors demonstrate the superiority of DR-GEM over standard analysis pipelines on a series of simulated datasets, spatial transcriptomics datasets, and Perturb-seq datasets, particularly in recovering rare cell clusters and accurately annotating cell states.
This work touches upon a central and important problem in the field of single-cell analysis. However, the manuscript in its current form has several issues that need to be addressed. Overall, at the algorithm design level, this study seems to have systematically overlooked a large number of algorithmic tools specifically designed for single-cell transcriptomics data, which often inherently account for the statistical properties of single-cell data (such as sparsity and overdispersion). Additionally, there is a lack of comparison with existing methods specifically designed for identifying rare cell types. It is debatable how far DR-GEM’s demonstrated superiority exceeds that of existing methods. In terms of experimental design, the study focuses more on recovering known rare cell types and fails to adequately demonstrate its ability to discover novel, unrevealed rare cell populations in large datasets.
Specifically, the authors need to address the following major and minor concerns.
Major Concerns
1.The core mechanism of DR-GEM relies on the initialization in Phase I, which uses cells with high reconstruction error (RE) to construct the initial embedding. This is a critical step, but its robustness is a concern. In many real-world datasets, cells with high RE may not be driven by rare biological signals but rather by insufficiently filtered technical noise, such as cell doublets, low-quality cells, or edge cells from batch effects. In such cases, DR-GEM's initialization step might focus on the wrong signals, and the subsequent balanced sampling and ensemble learning could amplify rather than correct this initial bias. We suggest the authors provide supplementary experiments to systematically evaluate the performance of DR-GEM in these more challenging scenarios.

2.The method essentially relies on the core assumption that "rare cells tend to have high reconstruction errors." However, the universality of this assumption in real data needs stronger evidentiary support. First, we recommend that the authors explicitly state the theoretical basis behind this assumption in the main text. Second, the number of datasets currently used to support this assumption is relatively limited. We suggest the authors to validate it on a more diverse set of public datasets (especially those containing experimentally verified rare cell types) to enhance the persuasiveness of this core assumption.

3.A major weakness of this manuscript is the lack of comprehensive comparisons with relevant state-of-the-art methods in the field.

In Figure 1, the authors use PCA as the basic dimensionality reduction tool to demonstrate the problem but do not compare it with other non-linear dimensionality reduction or embedding methods specifically designed for single-cell data (such as VAE-based models like scVI). Given that these methods have advantages in modeling the characteristics of single-cell data, re-evaluating the phenomenon of "high RE in rare cells" using them as a baseline would make the conclusions more convincing. It is also necessary to clarify whether the DR-GEM framework itself is affected by the choice of the underlying dimensionality reduction/reconstruction method.

Other advanced algorithms aimed at improving the detection of rare cell types already exist in the field (e.g., GiniClust). A comprehensive benchmark comparison of DR-GEM with these more challenging state-of-the-art methods is crucial for an objective assessment of its performance and novelty.

4.An important frontier in current single-cell biology is the exploratory analysis of large-scale cell atlas datasets to discover novel, functionally important rare cell subpopulations. However, all experiments in this manuscript are limited to validating the ability to recover "known" rare cell types. We strongly recommend that the authors apply DR-GEM to one or more large, incompletely annotated cell atlas datasets and attempt to identify new candidate rare cell types that can be validated by marker genes. This would greatly enhance the practical application value and impact of the method.

Furthermore, the simulation and real-data test scenarios in this study mostly involve only one major rare cell type. However,

in real complex tissues (such as the tumor microenvironment or developing organs), there are often multiple rare cell populations with different abundances. Whether DR-GEM's initialization and balanced sampling phases can handle multiple high-RE rare populations simultaneously is an unverified key question. We suggest the authors design simulation experiments involving multiple rare cell types to evaluate the performance and robustness of DR-GEM in such more realistic and complex scenarios.

5.The validation on real datasets relies on the annotations from the original studies as the "gold standard." This introduces a potential risk of circular reasoning, as the original annotations themselves may have been obtained through similar computational pipelines. We suggest the authors discuss this limitation and explore other validation strategies.

6.DR-GEM introduces several hyperparameters that could have a significant impact on the results. The manuscript lacks detailed guidance on the selection of these parameters and a systematic sensitivity analysis. We recommend that the authors provide a thorough analysis showing the impact of different choices within a reasonable parameter range on the final clustering results and rare cell identification performance to demonstrate the method's stability and ease of use.
Minor Concerns
1.When using non-linear dimensionality reduction methods (like Variational Autoencoders, VAEs) instead of PCA, their reconstruction error (RE) calculation and interpretation differ from PCA. How does the DR-GEM framework standardize or adjust the definition of RE to accommodate different dimensionality reduction algorithms, ensuring its effectiveness as a "hard example" metric is comparable across different algorithms?

2.In Phase II, when the number of clusters generated in an iteration is inconsistent with the number of initial "weak label" clusters (e.g., due to dynamic changes in clustering resolution), how does the Kuhn-Munkres alignment strategy handle this? Is there a mechanism for merging or splitting clusters?

3.The final step of DR-GEM is to project all cells onto the reference embedding space constructed from high-confidence cells. What is the main consideration behind adopting this projection method rather than directly using the original embeddings of all cells combined with consensus clustering labels for visualization? Could this approach potentially lose unique variation information from cells not selected for the reference set?

4.In a cell population that is highly heterogeneous but numerically dominant (such as tumor cells with high intrinsic heterogeneity), outlier cells within it may also exhibit high RE. In this case, would DR-GEM's initialization step mistakenly focus on this internal heterogeneity rather than on a truly independent rare cell type?

5.The simulated data used in the manuscript are mainly based on discrete cell types with clear boundaries. However, many biological processes manifest as continuous transcriptional trajectories. DR-GEM relies on discrete "weak labels" for balanced sampling, and this mechanism may face challenges when dealing with continuous data structures. We suggest the authors to discuss or experimentally validate their method's potential performance on such data.

6. The iterative process of DR-GEM seems focused on optimizing and stabilizing existing cluster assignments. If a genuinely existing rare cell type fails to form an independent cluster (even a small one) in the initial clustering, can DR-GEM "create" this new cluster in subsequent steps? Does this, to some extent, limit the algorithm's ability to discover entirely new, unforeseen cell types?

7.We suggest the authors provide a more detailed explanation of what types of cells generally constitute those with high confidence scores, and whether this score is related to the abundance or heterogeneity of specific cell types.

8.To more intuitively demonstrate the working mechanism of DR-GEM, the authors could consider adding a supplementary figure that visually illustrates the evolution of a rare cell cluster from its initial "weak label" to the final consensus label (e.g., the change in the number of cells it contains and its purity). This would also help to illustrate the necessity of the consensus clustering step.
Typographical Errors
In the caption of Figure 1B, "synthetic" is misspelled as "dynthetic".

(Remarks on code availability)

Version 1:

Reviewer comments:

Reviewer #1

(Remarks to the Author)
Authors have addressed most of my comments, which is good and I appreciate their efforts. However, the mathematical guarantees of their methods are still weak as I mentioned in previous comment 5/6. It would be better for the author to give a better illustration.

Point 5, especially point 6. The author provides empirical convergence analysis, an auto-convergence mechanism, and a

confidence score, but doesn't actually provide a formal convergence proof or error bounds. Therefore, this point should be further clarified.

(Remarks on code availability)
The code is good now.

Reviewer #2

(Remarks to the Author)
I co-reviewed this manuscript with one of the reviewers who provided the listed reports. This is part of the Nature Communications initiative to facilitate training in peer review and to provide appropriate recognition for Early Career Researchers who co-review manuscripts.

(Remarks on code availability)

Reviewer #3

(Remarks to the Author)
The revised manuscript and the supplementary materials are comprehensive, and the authors have rigorously addressed the concerns raised in the first round of review. We have no remaining major issues regarding the main content of the manuscript. We recommend that the authors briefly discuss the following minor points in the Discussion or Supplementary Information.

1. The new auto-convergence feature in Phase II is a practical addition. However, as spatial transcriptomics datasets continue to grow rapidly (such as merged cohorts with millions of cells or Visium HD data), the repeated dimensionality reduction and matching steps will still take a heavy toll on memory and runtime. To help users manage VRAM and computing bottlenecks, we suggest noting DR-GEM's resource usage for large-scale data or future GPU optimization plans in the Supplementary Information.

2. One interesting functionality is the "annotations-in-the-loop" mode for label mapping. How does the model behave when the user-provided signature dictionary is incomplete? Specifically, is there a risk that this mode might force unknown rare cells sharing basic lineage markers into existing categories? A brief discussion on the algorithm's ability to robustly retain such cells as "Undetermined" would be valuable to ensure that novel subpopulations are not inadvertently masked.

3. The authors suggest using the "annotations-in-the-loop" mode to manage the massive internal heterogeneity of dominant "mega-groups" like malignant cells. If the initial splits predominantly capture the intra-tumor heterogeneity of a mega-group, is there a risk that rare microenvironmental cells are overlooked?

4. The authors noted that stromal cells exhibited high reconstruction error in the imaging mass cytometry dataset due to "feature-bias" rather than cell rarity. To what extent might severe feature-imbalance skew the Phase I initialization in targeted panels? We suggest adding a note in the Discussion on whether users should preprocess or adjust highly skewed targeted panels before running the algorithm.

5. The authors have thoroughly clarified that their method is termed "self-supervised" because it leverages reconstruction error (RE) and weak label consistency as internal supervisory signals. However, with the fast growth of foundation models in single-cell and spatial omics, the term "self-supervised learning" now mostly makes people think of masked modeling or contrastive learning. At its core, DR-GEM's mechanism is actually closer to bootstrapped ensemble learning and distributionally robust optimization. To avoid confusing the readers, we suggest adding a short discussion in the manuscript. This discussion should simply point out the differences between DR-GEM's signal-driven self-supervision and the common "representational self-supervision" used in deep learning models today.

(Remarks on code availability)

Version 2:

Reviewer comments:

Reviewer #1

(Remarks to the Author)
Author has addressed my concerns. I have no more questions.

(Remarks on code availability)

Reviewer #2

(Remarks to the Author)
I co-reviewed this manuscript with one of the reviewers who provided the listed reports. This is part of the Nature Communications initiative to facilitate training in peer review and to provide appropriate recognition for Early Career Researchers who co-review manuscripts.

(Remarks on code availability)

Reviewer #3

(Remarks to the Author)
My questions have been resolved, and I have no further concerns.

(Remarks on code availability)

Open Access This Peer Review File is licensed under a Creative Commons Attribution 4.0 International License, which permits use, sharing, adaptation, distribution and reproduction in any medium or format, as long as you give appropriate credit to the original author(s) and the source, provide a link to the Creative Commons license, and indicate if changes were made.
In cases where reviewers are anonymous, credit should be given to 'Anonymous Referee' and the source.
The images or other third party material in this Peer Review File are included in the article's Creative Commons license, unless indicated otherwise in a credit line to the material. If material is not included in the article's Creative Commons license and your intended use is not permitted by statutory regulation or exceeds the permitted use, you will need to obtain permission directly from the copyright holder.
To view a copy of this license, visit https://creativecommons.org/licenses/by/4.0/

We thank the reviewers for their thoughtful comments and helpful feedback. We were pleased to see that the Reviewers appreciate the technical problems we surface and the biological relevance of our work. We addressed the Reviewer's comments by application of DR-GEM to additional datasets, new analyses, benchmarks of DR-GEM against other methods, and mathematical proofs, along with further clarifications, revised text, and figures. Below we highlight the key revisions, followed by a point-by-point response to each one of the questions and comments raised by the reviewers.

The key highlights of our revisions are:

Strengthening DR-GEM analytical foundation

- **Analytically demonstrating that rare cell type gene covariance scales with cell type frequency**, explaining why rare cells are systematically prone to high reconstruction error (RE) in PCA-based dimensionality reduction.
- **Analytically demonstrating that class imbalance can also hamper the sensitivity of abundant cell type detection**, as the covariance of their marker genes is more strongly linked to their covariance within the cell type they represent.
- **Empirically showing that cell type frequency impacts estimated variance and covariance of marker genes** in diverse simulated and real-world data.
- **High RE values are not associated with technical artifacts**, such as cell multiplets (or ambient RNA), low-quality cells, or batch effects. DR-GEM is also highly performant on these technically confounded datasets.
- **Targeted sequencing can also yield high RE values for cells that are underrepresented due to feature bias**, as evidenced in a breast cancer imaging mass cytometry dataset.

Comprehensive benchmarking shows that DR-GEM outperforms diverse methods

- Alternative DR-GEM variants with **Class-weighted PCA** and **SMOTE-PCA**, demonstrating the advantages of the DR-GEM pipeline and the limitations of these methods.
- DR-GEM outperforms the standard pipeline also when both are applied with **Kernel PCA (polynomial), Latent Dirichlet Allocation (LDA)**, and the Variational Autoencoder (**VAE)-based method, scVI**. In all settings, rare/minority cell types have elevated RE, and DR-GEM corrects this bias.
- Benchmarking DR-GEM against three conceptually distinct **rare cell type detection methods (GiniClust3, scCAD, and CIARA) across 9 single cell datasets**, showing DR-GEM outperforms these methods across diverse biological contexts and data types.

Applications to complex datasets

- **DR-GEM performance generalizes to settings with multiple concurrent rare populations**, correctly annotating 90–98% of each rare type, compared to 0% with the standard pipeline. Its central hypothesis holds even in extreme cases, such as data simulated with 100 unique minority cell types and in a real-world tracheal epithelium scRNA-seq dataset with 4 minority/rare cell types.
- **DR-GEM improves the representation of underrepresented patients and discovers a previously undetected, histologically validated malignant subclone in a large-scale spatial transcriptomic dataset.**
  - **Inter-patient heterogeneity**: With both DR-GEM and the standard pipelines, most malignant cells clustered by patient; however, with the standard pipeline, 75% of underrepresented patients were not assigned a patient-specific cluster, compared to only 33% with DR-GEM.
  - **Intratumor heterogeneity**: DR-GEM uncovered a novel cluster (0.29% of cells) from one patient that maps to a spatially and morphologically distinct subclone, previously identified and marked by a board-certified pathologist reviewing the matched H&E.

Sensitivity analyses, guidelines, and new functionalities for robust hyperparameter selection

- **Hyperparameter sensitivity analyses** track the impact of the number of PCA dimensions, Phase I RE threshold (β), and Phase II iteration count (r).
- **New code functionality for automatic hyperparameter selection** of r, alongside convergence tracking to monitor progress, and efficient addition of iterations with minimal computational cost.

- Demonstration that low confidence scores can result from (i) ambient RNA signal bleeding in spatial single cell data, (ii) cell doublets or multiplets in droplet-based data, and (iii) cell state transitions in single cell datasets with more continuous structure.

To facilitate the review process, the new parts added to the revised manuscript are marked in blue. The results of new analyses are depicted in new figures (new **Fig. 5, 7**, new **Supplementary Figs. 1, 2E-F, 3-8, 10-12**).

Reviewer #1 (Remarks to the Author):

The authors propose a novel dimensionality reduction method for single-cell analysis, addressing two key limitations of traditional approaches: (1) the distortion of rare cell type distributions in low-dimensional embeddings, and (2) sensitivity to technical noise (e.g., empty droplets). While the identified problem is compelling and biologically relevant, the methodological novelty and rigor require further clarification.

We thank the Reviewer for the thorough review, insightful feedback, and appreciation of our work. Addressing the Reviewer's comments, we substantially augmented and revised the manuscript with an array of new analytical and empirical demonstrations along with comprehensive benchmarking against various alternative methods and across diverse data types and biological contexts. We believe this substantially improved the study and clarifies the novelty and rigor of our method, as described below.

Major comments:

1. The author's question about rare cell types being mixed up is interesting. This may occur because rare cell types often exhibit lower gene expression variance compared to the majority cell types. PCA, being a linear projection method, selects axes based on feature variance. If rare cell types are poorly represented after reduction, it suggests their distinguishing gene expression signals are overshadowed by the high-variance signals of the majority cell types. I suggest authors to examine this point:

We thank the Reviewer for surfacing the need to investigate and demonstrate this property. We revised the manuscript to show how the relative abundance of a cell type impacts the (co)variance of its marker genes in the entire data. We show this both analytically and empirically, as described below.

<u>a) Analytically demonstrating the cell type frequency to marker gene covariance link</u>

The relative abundance of a cell type impacts the covariance of its marker genes in the data, explaining the propensity of rare cell types to have higher RE values in Principal Component (PC) space. The PCs of a dataset are the eigenvectors of the original feature covariance matrix, sorted based on their eigenvalues. The eigenvalues are proportional to the variance explained by the respective PC.

The covariance of two genes X and Y in a population P of N cells can be represented as

$$Cov_P(X,Y) = \mathbb{E}_P\left[\left(X - \mathbb{E}_P(X)\right)\left(Y - \mathbb{E}_P(Y)\right)\right] = \mathbb{E}_P[XY] - \mathbb{E}_P(X)\mathbb{E}_P(Y) = A - B$$

For any split of population P to G groups of cells ($P_1, \ldots P_G$) the term A can be represented as:

$$A = \mathbb{E}_P[XY] = \sum_{i \in G} w_i * \mathbb{E}_{P_i}[XY] = \sum_{i \in G} w_i * \left(Cov_{P_i}(X,Y) + \mathbb{E}_{P_i}[X]\mathbb{E}_{P_i}[Y]\right)$$

Where $w_i = \frac{|P_i|}{N}$

As covariance is invariant to data centering, the property is analyzed when considering the data prior to centering. In the case that X and Y are marker genes of a specific group of cells P_j, their average expression and covariance will be significantly lower in other groups of cells P_i ($i \neq j$).

By definition

$$\forall i \neq j: Cov_{P_i}(X,Y) \ll Cov_{P_j}(X,Y), \mathbb{E}_{P_i}[X] \ll \mathbb{E}_{P_j}[X], \mathbb{E}_{P_i}[Y] \ll \mathbb{E}_{P_j}[Y]$$

For simplicity, let the marker genes of population j be expressed only in P_j.

$$\forall i \neq j: Cov_{P_i}(X,Y) = 0, \mathbb{E}_{P_i}[X] = 0, \mathbb{E}_{P_i}[Y] = 0$$

A can be further simplified

$$A = \mathbb{E}_P[XY] = \sum_{i \in G} w_i * \mathbb{E}_{P_i}[XY] = w_j * \mathbb{E}_{P_j}[XY]$$

And the average expression of a marker gene (e.g., X) in the whole population P is

$$\mathbb{E}_P(X) = \sum_{i \in G} w_i * \mathbb{E}_{P_i}(X) = w_j * \mathbb{E}_{P_j}[X]$$

Together

$$Cov_P(X,Y) = A - B = w_j * \left(Cov_{P_j}(X,Y) + \mathbb{E}_{P_j}[X]\mathbb{E}_{P_j}[Y] \right) - \left(w_j * \mathbb{E}_{P_j}[X] \right) \left(w_j * \mathbb{E}_{P_j}[Y] \right)$$

Let

$$C = \mathbb{E}_{P_j}[X]\mathbb{E}_{P_j}[Y]$$

$$Cov_P(X,Y) = A - B = w_j * \left(Cov_{P_j}(X,Y) + C \right) - w_j^2 * C$$

$$= w_j * Cov_{P_j}(X,Y) + w_j\left(1 - w_j\right) * C$$

The covariance can thus be represented as a weighted average of marker gene strength and within cell type covariance

$$Cov_P(X,Y) = \underbrace{w_j * Cov_{P_j}(X,Y)}_{\substack{Within\ cell\ type \\ covariance}} + \underbrace{w_j\left(1 - w_j\right) * \mathbb{E}_{P_j}[X]\mathbb{E}_{P_j}[Y]}_{Cell\ type\ gene\ marker\ strength}$$

As shown in **PBP Fig. 1** below, the term $w_j(1 - w_j)$ is at most 0.25 (when $w_j = 0.5$) and is monotonically increasing with w_j when $w_j \in [0,0.5)$.

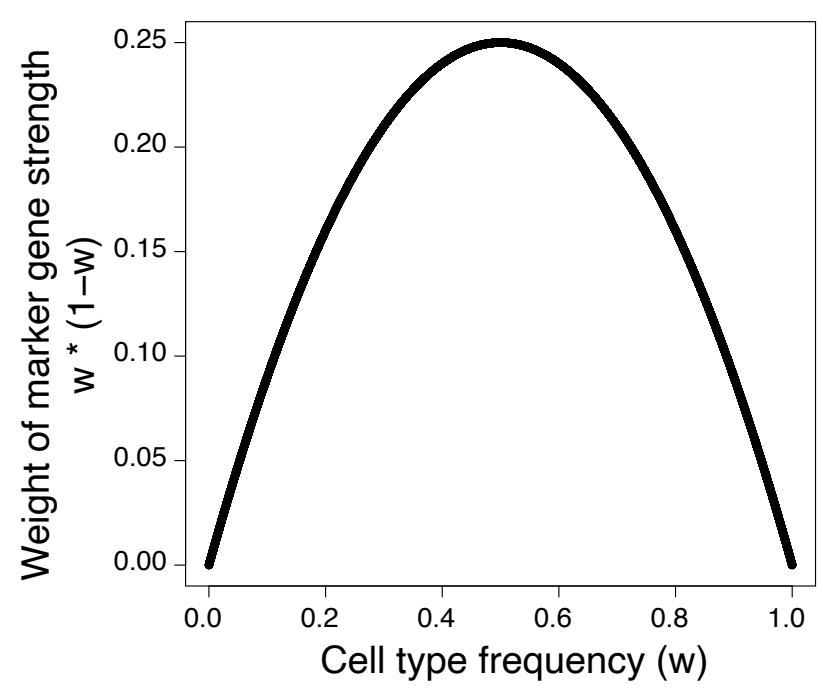

PBP Fig. 1

As the data is not centered, $C \geq 0$ and the covariance between marker genes in the relevant cell type population is expected to be positive, i.e., $Cov_{P_j}(X,Y) \geq 0$.

Thus, for $w_j \in [0,0.5)$ the lower w_j the lower the covariance of X and Y is in the entire population.

When $w_j \in (0.5,1]$ the covariance of marker genes of population P_j depends more on their covariance within P_j, and less so on the level of their expression in the cell type they mark. If the marker genes of P_j do not covary within P_j, then their covariance will drop with the increase in w_j once $w_j > 0.5$. $w_j = 0.5$ thus represents a tipping point, above which PCA will focus more on the within (dominant) cell type variation compared to the cross-cell-type variation.

By rebalancing the data DR-GEM hence obtains a clearer distinction between cell types for both the rare and more abundant cell types, consistent with the notion that the former is blended with the latter in unbalanced settings. This also demonstrates why a subsequent embedding per cell type population is needed, where the intra-cell-type covariation can be captured more accurately without being tied to cross-cell-type variation. DR-GEM improve cell type annotations and hence this downstream cell-type-specific analyses will be less likely to be contaminated by signals coming from other cell types as misassigned rare cells.

<u>b) Cell type frequency impacts marker gene covariance in simulated data</u>

In our synthetic datasets, all synthetic cell types are drawn from generative models with the same parameters, meaning, the same number of marker genes and gene variance for marker and non-marker genes (new **Supplementary Fig. 1**, **Methods**).

Supporting the analytical observations described above we now show that:

- The variance of rare cell type marker genes within the rare cell type is comparable to the variance of other cell type marker genes within the cell type they represent (new **Supplementary Fig. 1B-C**).
- Rare cell marker genes have significantly lower variance in the entire dataset compared to the variance of marker genes of other cell types (new **Supplementary Fig. 1D**).
- In accordance with the well documented mean-variance relationship of negative binominal distributions observed in real-world RNA-sequencing datasets, the variance of a marker gene is higher within the cell type it marks compared to its variance within other cell type populations (new **Supplementary Fig. 1B-C**).

c) Cell type frequency impacts marker gene covariance in real-world data

In both the SMI dataset profiling tubo-ovarian cancer tumors[1], and a MERFISH dataset profiling the preoptic hypothalamus[2], the within-cell-type variance of marker genes is invariant to cell type population size (new **Supplementary Fig. 3A, C**), whilst marker genes of rare cell type populations exhibit systematically lower variance across all the cells in the data (new **Supplementary Fig. 3B, D**). Further demonstrating this, T/NK cells compromise 15.4% of the data (*n* = 2,702) in the SMI tubo-ovarian cancer dataset, such that uniformly subsampling T/NK cells (0.36%, *n* = 50) decreases the variance of T/NK cell markers across the entire dataset (new **Supplementary Fig. 3E**).

Taken together, markers of rare cell type populations have low (co)variance in the entire population due to the low frequency of the cell type they mark, supporting DR-GEM's design rationale to up-sample cells with a high RE to mitigate the bias of estimated (co)variance under class imbalance.

These new analyses and findings are described and visualized in the revised manuscript (pages 3-5, lines 115-122, 137-140, 163-165; **Methods** pages 35-36, lines 786-834; new **Supplementary Figs. 1, 3**).

2. LDA, Kernel PCA, Class-weighted PCA and SMOTE+PCA should be tested.

We thank the reviewers for their suggestion to test these algorithms. By doing so we now demonstrate the modularity of the DR-GEM framework and benchmark certain components of DR-GEM to other approaches, as described below.

(1) DR-GEM framework modularity

We previously tested DR-GEM compared to the standard pipeline with both pipelines applied with standard PCA. To address the Reviewer's comment, we extended these tests by applying both DR-GEM and the standard pipelines to Synthetic Dataset 1 with other dimension reduction algorithms: (i) Kernel PCA with a polynomial (degree = 2) kernel (new **Supplementary Fig. 4**), (ii) Latent Dirichlet Allocation (LDA)[3] (new **Supplementary Fig. 5A-B**), and (iii) scVI[4] (new **Supplementary Fig. 5C-D**). In all three cases, DR-GEM improved the embedding of the minority/rare cell type and increased detection of the minority/rare cell type after clustering.

(2) Alternatives to Phase II-III of the DR-GEM framework

Class-weighted PCA and SMOTE followed by PCA both require labels. Thus, these methods cannot be used instead of PCA throughout the DR-GEM framework, but can be use as PCA alternatives in Phases II and III of DR-GEM, using the weak cluster labels from Phase I as input.

DR-GEM Phase I followed by class-weighted PCA with the weak cluster labels as input labels, was able to decrease the RE of the minority/rare cell population in Synthetic Dataset 1 (new **Supplementary Fig. 6A,** middle right). However, upon subsequent clustering and UMAP embedding on the class-weighted embedding space, the minority/rare cell population is poorly represented under UMAP visualization and mislabeled (new **Supplementary Fig. 6A,** left and middle left). Class-weighted PCA is similar to "Tilted Fair PCA" (**Fig. 2E**) in that both methods utilize explicit labels, reduce the RE discrepancy between the minority/rare cells and the majority cell types but are nonetheless unable to cluster the minority/rare cells apart from the other cells, resulting in mislabeling.

In contrast, DR-GEM Phase I followed by SMOTE followed by PCA reduced the RE discrepancy between the minority/rare cells and the majority cell types, correctly clustered and annotated all cell types, including the minority/rare cells (100% accuracy, new **Supplementary Fig. 6B,** right). DR-GEM can thus be used to identify minority/rare cells for downstream SMOTE-augmented analyses, including PCA and UMAP (new **Supplementary Fig. 6B**). However, investigating the PC and UMAP space reveals distortions of the minority/rare cell population expression landscape after SMOTE augmentation (new **Supplementary Fig. 6C**). More specifically, although the data was simulated with the same level of intra-cell-type variation across the five different cell types, including the rare one, the SMOTE embedding falsely depicts the rare cell type population as highly heterogenous compared to the other cell types, raising questions concerning the validity and reliability of the SMOTE augmentation.

In addition, applying DR-GEM with either class-weighted PCA or SMOTE-PCA does not include mechanisms to assess per-cell embedding and annotation robustness (i.e., per-cell confidence score s_i from DR-GEM) and to mitigate effects driven by ambient RNA (i.e., reference embedding of cells with high confidence scores). These aspects of DR-GEM are particularly important when analyzing spatial transcriptomics (**Figs. 6,8**) and other types of data confounded by ambient RNA (**Fig. 4B-H**).

These new analyses and findings are described and visualized in the revised manuscript (page 7, lines 262-280; **Methods**, pages 43-44, lines 1107-1126; new **Supplementary Fig. 4-6**).

3. If the method simply repeats subsampling and reprojection from scratch, how does it enhance embedding quality? The iterative process alone does not inherently guarantee better representations unless there's a mechanism that refines embeddings. It seems the core idea of this method is to select some cells for obtaining better projection matrix. Why is it a self-supervised learning technique?

We thank the Reviewer for surfacing the need to clarify these points.

As alluded to by the Reviewer, DR-GEM primarily improves the embedding by up-sampling rare cell type populations and obtaining a more balanced representation of different cell types, as shown in response to comment #1 and based on the empirical findings that rare cell types are more abundant among cells with high RE (e.g., **Fig. 1C-E,** new **Supplementary Fig. 2**). This is achieved in Phase I, namely, prior to any subsampling (Phase II) and reprojection (Phase III).

As the first balanced embedding ($E^{(init)}$) is based on a subset of the cells, the purpose of Phase II is to identify the cells with a distinct cell type identity, which will then be used to generate the final reference map in Phase III. This is particularly important in spatial transcriptomics data, where some cells have more ambient RNA and have a lower signal to noise ratio. More specifically, ambient contamination from imperfect cell segmentation in single-cell spatial 'omics or multiplets in single cell data result in variable levels of contamination, such that highly contaminated cells are characterized by unstable cluster assignments across balanced resamples, yielding low consensus scores s_i. Thus, in Phase III such cells are down-weighted when fitting the reference embedding, so the final mapping is less biased by ambient signals. Empirically supporting this, simulations of ambient RNA show that DR-GEM is significantly better at mitigating the effects of ambient RNA relative to the standard pipeline (**Fig. 4B-G**).

As alluded to by the Reviewer, applying Phase II with subsampling that just randomly selects a subset of cells from the data will no inherently guarantee better representations and hence it is important to subsample in a balanced manner across the initial cluster annotations obtained in Phase I and use the ensemble agreement scores to select the cells for the final reference map. We note that, the cells selected to generate the final reference map may not necessarily be those with the highest RE computed in Phase I.

As for the notion of self-supervision, we refer to DR-GEM as a self-supervised method because it uses:

1. **Reconstruction error (RE) as a self-supervisory signal.** In Phase I, DR-GEM first computes per-cell RE under a standard reconstruction mean-squared error. It then uses only a subset of cells in the right-tail of the RE distribution (β) to form an initial embedding. This procedure uses the RE distribution learned from the first fit as a diagnostic tool to upweight "at-risk" cells.

2. **"Weak" labels to balance representation.** In Phase II, DR-GEM utilizes initialized labels as "weak" (or "soft") labels to draw balanced subsamples. These weak labels were also learned from the data itself.
3. **Ensemble agreement for confidence-weighting.** In Phase III, DR-GEM computes a per-cell consensus score s_i and then uses high-confidence cells (with $s_i > t$) to fit a reference embedding. This reduces bias from outliers/contamination. The consensus scoring is also learned from the algorithm itself.

We revised the **Methods** to note why we consider DR-GEM as "self-supervised" (page 41, lines 1,001-1,003).

4. The high-confidence cells are selected via ensemble strategies. How does this specifically address noise from empty droplets? Since ensemble methods aggregate multiple predictions, they may reduce stochastic errors, but a clearer justification for droplet noise suppression would be helpful.

We thank the Reviewer for flagging the need to clarify these aspects and terminology. Because DR-GEM improves dimensionality reduction, it indirectly addresses drop**outs** (stochastic zero inflation of gene expression). DR-GEM is not designed to address noise from empty drop**lets**, which are typically removed upstream during standard QC (e.g. using UMI/gene thresholds to filter). We revised the **Methods** section to explain that users are recommended to filter out empty droplets before providing the data as input for DR-GEM (page 41, lines 1,005-1,006).

Briefly, DR-GEM mitigates drop**outs** from the functionality of Phase IV, which annotates cells in a manner that draws signal from other similar cells (in the same cluster) and uses cell type gene signatures (instead of just a single or handful of maker genes). These strategies underlying Phase IV of DR-GEM are not unique to DR-GEM and are routinely implemented to overcome sparsity in both bulk RNA-seq and scRNA-seq.

We have deemphasized discussion of empty droplets and dropouts, instead, we provide new analyses and discussion of DR-GEM's performance under common technical noise (systematic low read counts, batch effects, and multiplets/ambient RNA; page 8, lines 312-329; **Methods**, page 44, lines 1,128-1,161; **Fig. 4B-H,** new **Fig. 5**, new **Supplementary Fig. 7, 12A**).

5. In Phase I, the projection matrix is derived from high reconstruction error (RE) cells. Could this bias the embeddings for other cells?

DR-GEM improves cell type annotations in a manner that is most notable for minority/rare cell types but, as now shown analytically in response to comment #1 does in fact reorient the embedding to obtain a better cell type representation for abundant cell types as well (**Methods**, pages 35-36, lines 786-834). Empirically supporting this, when applied to Synthetic (**Figs. 4, 5**) and real-world data (**Figs. 6-10**), DR-GEM clusters a rare cell type apart from other cell types and hence also prevents the rare cells from "contaminating" the clusters of other cell types. We note that DR-GEM embeds the data based on the highest source of variation. In dataset that include multiple cell types variation is primarily due to cross-cell-type variation, but can also be due to other biological differences, as somatic genetic variation or exogenous genetic manipulation, as we show in the context of malignant inter-patient variation (new **Fig. 7**) and Perturb-seq screens (**Figs. 9-10**), respectively.

As demonstrated in response to comment #1 above, irrespective of which method is used to obtain the embedding of the entre dataset and cell type annotations, it is recommended to proceed with cell-type-specific (or cluster-specific) analyses to further identify gene modules and cell states within each cell type, where the covariance within a cell type will not be tied to the covariance across cell types. As DR-GEM better annotates cells to cell types (or any other high level distinct groups), it helps provide better parsing of the cells for these downstream analyses.

To further address the Reviewer's question, we examined the embeddings obtained in Phase I and Phase III when DR-GEM is applied to Synthetic Dataset 1. While DR-GEM significantly decreases the RE of the rare /minority cell population, it does so without causing a significant increase in the RE of other cells (new **Supplementary Fig. 10D**).

If Phase II operates on these potentially skewed embeddings, how does it correct or improve them?

Phase II operates on the initial solution in Phase I to take advantage of the benefits of DRO, but treats the weak labels produced in Phase I as provisional rather than definitive. A cell can be reassigned to a different cluster in Phase II based on the majority vote. Moreover, when DR-GEM is applied with annotations-in-the-loop, the clusters of Phase II are not mapped to those obtained in Phase I. In Phase III, clusters are redefined and DR-GEM does not enforce the reference map clusters to match the clusters obtained in Phase I. Lastly, in testing DR-GEM across a total of 9 datasets (revised **Supplementary Table 2**), there is no evidence that DR-GEM misses clusters that would otherwise form with the standard pipeline or generates pseudo-clusters that do not represent true variation (e.g., ground-truth annotations in synthetic data, well-established cell (sub)types in spatial transcriptomics data, genetic perturbations in Perturb-seq data).

6. Are there mathematical guarantees (e.g., convergence properties, error bounds) ensuring that Phase II refines embeddings rather than amplifying initial biases?

We now provide analytical demonstration (described in response to comment #1; revised **Methods**, pages 35-36, lines 786-834), along with the empirical confirmation that Phase II results in a more accurate representation of marker gene variance that is less prone to sample bias (new **Supplementary Table 1**).

As we now show by tracking Phase II convergence across iterations (new **Supplementary Fig. 11**), convergence depends on the degree of latent class imbalance in the input dataset and the number of cells impacted by ambient RNA and other sources of noise. For example, in the Perturb-seq data (new **Supplementary Fig. 11D**), there is a steady increase in prediction accuracy with each iteration, until the prediction accuracy plateau above ~150 iterations. As expected, the number of iterations required to sample each cell type population scales with the size of the population. To support users in selecting the number of Phase II iterations to reach convergence, we have now added a new optional functionality where:

(1) DR-GEM assesses every *r1* iterations after all cells have been sampled at least 5 times how much the annotations change for each final assigned label (δ_k). If for all K classes/labels, the change of proportion of assignments ($\delta_1 \ldots \delta_K$) are below δ_{target} (default=0.05), DR-GEM can terminate earlier than initially specified by *r*.
(2) A user can add *r2* additional iterations without repeating the full run to reach convergence.

Regarding error, the score s_i computed in Phase III serves as an empirical error estimate of each cell type label.

These new analyses and features are presented and discussed in the revised manuscript (pages 5-6, lines 115-122, 223-225; **Methods**, pages 35-36, lines 786-834, page 42, lines 1,052-1,061; new **Supplementary Fig. 11,** new **Supplementary Table 1**).

Minor comments:

1. P_1 (instead of P1) should be subindexed for proper mathematical notation in Results section

Thank you. We have fixed this in the text; page 4, line 128.

2. How is the second embedding learned? Is PCA applied again, using the same projection matrix to obtain embeddings for all cells?

In Phase I of DR-GEM, the second embedding $E^{(init)} \in \mathbb{R}^{n^{(0)} \times m}$ is learned from fitting PCA to only the cells in the top β percentile of the RE distribution. The remaining cells are projected onto the subspace spanned by $E^{(init)}$ (by using the projection matrix obtained by fitting $E^{(init)}$ to yield $E^{(1)} \in \mathbb{R}^{n \times m}$ for all cells). We have modified the language in the **Methods** to clarify this (page 41, lines 1,012-1,015).

3. In Phase II, the description states: "DR-GEM then fits r (user-specified) learners that yield r sets of predictions fit on r balanced subsamples of the data." (a) Does this mean clustering is performed *r* times on

subsamples? (b) The phrasing "fit learners and make predictions" could be misinterpreted as training a supervised classifier using cell labels. If unsupervised, the wording should be clarified.

This is indeed an unsupervised process, and we apologize for the inappropriate terminology. We revised the text to explain that clustering is performed *r* times and then the clusters obtained within each subsample are matched to the initial cluster labels, referred to as $c^{(1)}$, obtained in Phase I (page 41, lines 1,017-1,018). We revised the manuscript throughout to refrain from referring to this process as fitting learners and making predictions.

4. I do not think that direct using cluster annotation to supervise cell embeddings is a good choice (Phase IV). This may hurt other potential biological signals in the cells, as indicated by (https://www.nature.com/articles/s41587-025-02702-z). Author should discuss the potential limitation when using this optional phase.

We appreciate that tools like scGraph, referred to by the Reviewer, can run topology-preservation diagnostics to examine whether the embedding preserves the distance across clusters (or cell types). Phase IV is based on the assumption that cells of the same cell type will cluster together and overexpress the cell type signature compared to other clusters. It does not rely on inter-cluster distances. To address the Reviewer's comment, we revised the Discussion to refer to scGraph as a valuable diagnostic tools to investigate DR-GEM output (page 14, lines 577-581) and provide additional outputs to compare and contrast DR-GEM and the standard embeddings (page 6, lines 240-245).

Reviewer #1 (Remarks on code availability):

Code availability: Code is well written and provide a comprehensive tutorial. However, the link (https://github.com/Jerby-Lab/fair-lin-ae) is invalid.

We are glad the Reviewer finds that the code is well written. We apologize that https://github.com/Jerby-Lab/fair-lin-ae, including the DR-GEM R package, was not accessible online at the time of the first submission. We have now made it publicly available for review.

Reviewer #2 (Remarks to the Author):

I co-reviewed this manuscript with one of the reviewers who provided the listed reports. This is part of the Nature Communications initiative to facilitate training in peer review and to provide appropriate recognition for Early Career Researchers who co-review manuscripts.

We thank this reviewer for taking the time to co-review. The thoughtful comments and questions have all been very helpful in improving our work.

Reviewer #2 (Remarks on code availability):

Code is well written and provide a comprehensive tutorial. However, the link (https://github.com/Jerby-Lab/fair-lin-ae) is invalid.

We thank the Reviewer for their positive feedback on our code for the DR-GEM R package. We apologize that https://github.com/Jerby-Lab/fair-lin-ae was not accessible online. We have now made it publicly available for review.

Reviewer #3 (Remarks to the Author):

This manuscript introduces a self-supervised meta-algorithm named DR-GEM, designed to address the limitations of standard dimensionality reduction and clustering methods in identifying rare cell types and distinguishing biological signals from technical noise in single-cell and spatial omics analysis. The authors propose that existing methods tend to overfit to the dominant patterns in the data, thereby ignoring or incorrectly embedding rare cell populations. DR-GEM addresses this issue through an innovative multi-phase framework that integrates hard-example identification based on reconstruction error, balanced ensemble learning, and the construction of a high-confidence reference atlas. The authors demonstrate the superiority of DR-GEM over standard analysis pipelines on a series of simulated datasets, spatial transcriptomics datasets, and Perturb-seq datasets, particularly in recovering rare cell clusters and accurately annotating cell states.

This work touches upon a central and important problem in the field of single-cell analysis. However, the manuscript in its current form has several issues that need to be addressed. Overall, at the algorithm design level, this study seems to have systematically overlooked a large number of algorithmic tools specifically designed for single-cell transcriptomics data, which often inherently account for the statistical properties of single-cell data (such as sparsity and overdispersion). Additionally, there is a lack of comparison with existing methods specifically designed for identifying rare cell types. It is debatable how far DR-GEM's demonstrated superiority exceeds that of existing methods. In terms of experimental design, the study focuses more on recovering known rare cell types and fails to adequately demonstrate its ability to discover novel, unrevealed rare cell populations in large datasets.

We thank the Reviewer for the insightful review and valuable comments. Addressing the comments raise by the Reviewer, we substantially expanded the manuscript in the three areas highlighted here: algorithmic grounding, benchmarking against relevant methods, and demonstration of biological discovery beyond recovery of known rare populations.

First, we now provide a stronger analytical and empirical foundation for the central design of DR-GEM. We analytically show that cell type frequency directly shapes marker-gene covariance and therefore reconstruction error in PCA-based dimensionality reduction, explaining why minority populations are prone to poor representation. We further show that this is not only a rare-cell problem: class imbalance can also reduce separation of abundant populations. We also directly test whether high reconstruction error may be associated with technical artifacts and show that this is not the case for multiplets, low-quality cells, or batch effects, and that DR-GEM remains highly performant in these technically confounded settings.

Second, we substantially broadened benchmarking against methods relevant to single-cell data. We now test DR-GEM with alternative embedding backbones that explicitly model nonlinearity or single-cell-specific structure, including Kernel PCA, LDA, and scVI, and show that the core phenomenon motivating DR-GEM persists across all of them. We also benchmark DR-GEM directly against three conceptually distinct rare-cell detection frameworks, GiniClust3, scCAD, and CIARA, across diverse datasets and data types. These analyses show that the strength of DR-GEM is substantial and consistent across methods, modalities, and biological settings, rather than being specific to the standard PCA-based pipeline.

Third, we extended the study beyond recovery of previously annotated rare populations to address discovery in larger and more complex datasets. We now show that DR-GEM generalizes to settings with multiple concurrent rare populations, including extreme simulations with 100 minority classes and a real tracheal epithelium dataset with four rare cell types. Importantly, in a large spatial transcriptomic tubo-ovarian cancer dataset, DR-GEM improved recovery of underrepresented patient-specific malignant states and identified a previously undetected malignant subclone that maps to a spatially and morphologically distinct region and was validated against matched H&E by a board-certified pathologist.

Together, these additions directly address the concerns raised in the overview and substantially strengthen the manuscript across theory, benchmarking, and discovery, as we described in a point-by-point response below.

Specifically, the authors need to address the following major and minor concerns.

Major Concerns

1.The core mechanism of DR-GEM relies on the initialization in Phase I, which uses cells with high reconstruction error (RE) to construct the initial embedding. This is a critical step, but its robustness is a concern. In many real-world datasets, cells with high RE may not be driven by rare biological signals but rather by insufficiently filtered technical noise, such as cell doublets, low-quality cells, or edge cells from batch effects. In such cases, DR-GEM's initialization step might focus on the wrong signals, and the subsequent balanced sampling and ensemble learning could amplify rather than correct this initial bias. We suggest the authors provide supplementary experiments to systematically evaluate the performance of DR-GEM in these more challenging scenarios.

We thank the Reviewer for emphasizing the need to test whether high RE can result from common technical artifacts. To address this important comment, we tested the RE values and DR-GEM with new simulated datasets of low-quality cells (i.e. systematically low reads, new **Fig. 5A**) and batch effects mimicking "autofluorescence" (new **Fig. 5B**), providing a more comprehensive evaluation of different types of technical confounders, in addition to the previous simulations of cell multiplets and ambient RNA in Synthetic Dataset 2 (**Fig. 4B-H**). According to these simulations, transcriptomic profiles impacted by these three types of noise are not enriched and are in fact underrepresented in the right tail of the RE distribution (new **Supplementary Fig 7**). Latent class imbalance remains the main correlate of high RE in each of these settings ($p < 4 \times 10^{-14}$, RE of rare vs. all other cell types, one-sided Wilcoxon rank sum test in all 3 settings; new **Figs. 4B, 5A-B)**. DR-GEM detected 98% and 96% of the rare Cell Type 5 when applied to the data simulated with low-quality cells and batch effect (new **Fig. 5A-B**), while the standard pipeline falsely annotated all the rare cells in both settings. This is aligned with the previous results, showing DR-GEM's performances when applied to Synthetic Dataset 2 with cell multiplets and ambient RNA (**Fig. 4B-H**). These new simulations and results are described a new Results section (page 8, lines 312-329) and **Methods** (page 44, lines 1,141-1,147) and figures (new **Fig. 5**, new **Supplementary Fig. 7B-C**).

Interestingly, in a breast cancer imaging mass cytometry dataset[5], stromal cells are also disproportionately enriched in the right tail of the RE distribution (new **Supplementary Fig. 2D**), despite being the second largest cell type subpopulation of the dataset. In this dataset profiling 35 marker proteins, stromal cells have the fewest marker genes in the panel, posing a different bias to this subpopulation in dimensionality reduction, related to the rarity of features as opposed to the rarity of the cells. Yet, despite the additional enrichment of the stromal cells in the right tail of the RE distribution, DR-GEM was still able to decrease the RE of the rare cell type (B cells, new **Supplementary Fig. 10E**) and to improve their detection to 88% from 0% in the standard pipeline. These findings suggest that high RE values mark biologically meaningful classes that are underrepresented either due to sampling or feature-bias, supporting the procedures implemented in Phase I of the algorithm

2.The method essentially relies on the core assumption that "rare cells tend to have high reconstruction errors." However, the universality of this assumption in real data needs stronger evidentiary support. First, we recommend that the authors explicitly state the theoretical basis behind this assumption in the main text. Second, the number of datasets currently used to support this assumption is relatively limited. We suggest the authors to validate it on a more diverse set of public datasets (especially those containing experimentally verified rare cell types) to enhance the persuasiveness of this core assumption.

We thank the Reviewer for surfacing the need to better justify and explain the theoretical and empirical basis for DR-GEM algorithmic design. To address this, we now provide analytical proofs and more extended empirical demonstrations to support DR-GEM design, as described below.

<u>1) Analytically demonstrating that rare cells are prone to have high RE in Principal Component (PC) space (revised manuscript, pages 4-5, lines 115-122; **Methods**, pages 35, lines 786-834):</u>

The principal components (PCs) of the data are the eigenvectors of the original features' (e.g., genes') covariance matrix, sorted based on their eigenvalues, which denote the variance each PC explains. As we show below, the relative abundance of a cell type impacts the covariance of its marker genes in the data, explaining the propensity of rare cell types to have high RE in the context of PCA.

The covariance of two genes X and Y in a population P of N cells can be represented as

$$Cov_P(X,Y) = \mathbb{E}_P\left[\left(X - \mathbb{E}_P(X)\right)\left(Y - \mathbb{E}_P(Y)\right)\right] = \mathbb{E}_P[XY] - \mathbb{E}_P(X)\mathbb{E}_P(Y) = A - B$$

For any split of population P to G groups of cells ($P_1, \dots P_G$) the term A can be represented as:

$$A = \mathbb{E}_P[XY] = \sum_{i \in G} w_i * \mathbb{E}_{P_i}[XY] = \sum_{i \in G} w_i * \left(Cov_{P_i}(X,Y) + \mathbb{E}_{P_i}[X]\mathbb{E}_{P_i}[Y]\right)$$

Where $w_i = \frac{|P_i|}{N}$

As covariance is invariant to data centering, the property is analyzed when considering the data prior to centering. In the case that X and Y are marker genes of a specific group of cells P_j, their average expression and covariance will be significantly lower in other groups of cells P_i ($i \neq j$).

By definition

$$\forall i \neq j: Cov_{P_i}(X,Y) \ll Cov_{P_j}(X,Y), \mathbb{E}_{P_i}[X] \ll \mathbb{E}_{P_j}[X], \mathbb{E}_{P_i}[Y] \ll \mathbb{E}_{P_j}[Y]$$

For simplicity, let the marker genes of population j be expressed only in P_j.

$$\forall i \neq j: Cov_{P_i}(X,Y) = 0, \mathbb{E}_{P_i}[X] = 0, \mathbb{E}_{P_i}[Y] = 0$$

A can be further simplified

$$A = \mathbb{E}_P[XY] = \sum_{i \in G} w_i * \mathbb{E}_{P_i}[XY] = w_j * \mathbb{E}_{P_j}[XY]$$

And the average expression of a marker gene (e.g., X) in the whole population P is

$$\mathbb{E}_P(X) = \sum_{i \in G} w_i * \mathbb{E}_{P_i}(X) = w_j * \mathbb{E}_{P_j}[X]$$

Together

$$Cov_P(X,Y) = A - B = w_j * \left(Cov_{P_j}(X,Y) + \mathbb{E}_{P_j}[X]\mathbb{E}_{P_j}[Y]\right) - \left(w_j * \mathbb{E}_{P_j}[X]\right)\left(w_j * \mathbb{E}_{P_j}[Y]\right)$$

Let

$$C = \mathbb{E}_{P_j}[X]\mathbb{E}_{P_j}[Y]$$

$$Cov_P(X,Y) = A - B = w_j * \left(Cov_{P_j}(X,Y) + C\right) - w_j^2 * C$$

$$= w_j * Cov_{P_j}(X,Y) + w_j\left(1 - w_j\right) * C$$

The covariance can thus be represented as a weighted average of marker gene strength and within cell type covariance

$$Cov_P(X,Y) = \underbrace{w_j * Cov_{P_j}(X,Y)}_{\substack{\text{Within cell type} \\ \text{covariance}}} + \underbrace{w_j\left(1 - w_j\right) * \mathbb{E}_{P_j}[X]\mathbb{E}_{P_j}[Y]}_{\text{Cell type gene marker strength}}$$

As shown in **PBP Fig. 1** below, the term $w_j\left(1 - w_j\right)$ is at most 0.25 (when $w_j = 0.5$) and is monotonically increasing with w_j when $w_j \in [0,0.5)$.

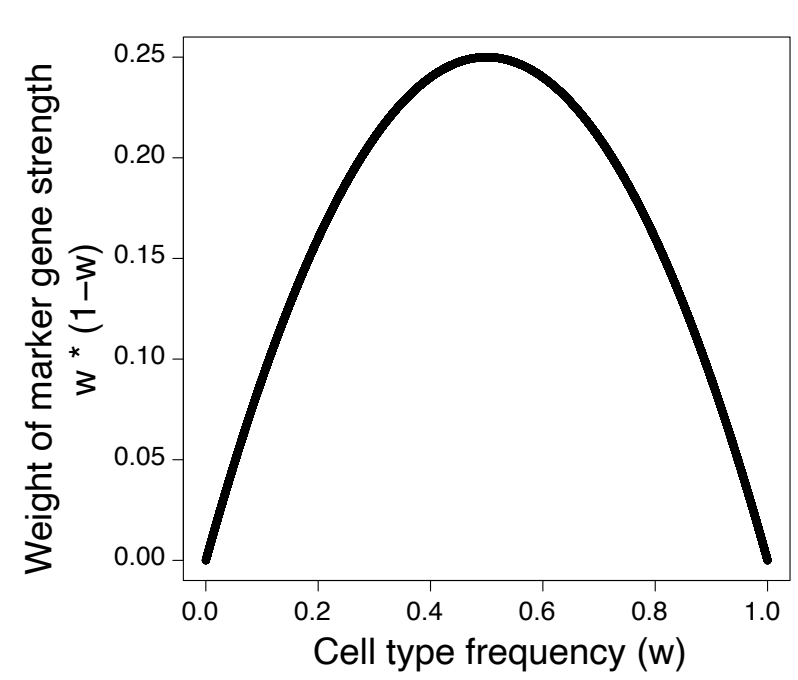

PBP Fig. 1

As the data is not centered, $C \geq 0$ and the covariance between marker genes in the relevant cell type population is expected to be positive, i.e., $Cov_{P_j}(X, Y) \geq 0$.

Thus, for $w_j \in [0,0.5)$ the lower w_j the lower the covariance of X and Y is in the entire population.

When $w_j \in (0.5,1]$ the covariance of marker genes of population P_j depends more on their covariance within P_j, and less so on the level of their expression in the cell type they mark. If the marker genes of P_j do not covary within P_j, then their covariance will drop with the increase in w_j once $w_j > 0.5$. $w_j = 0.5$ thus represents a tipping point, above which PCA will focus more on the within (dominant) cell type variation compared to the cross-cell-type variation.

By rebalancing the data DR-GEM hence obtains a clearer distinction between cell types for both the rare and more abundant cell types, consistent with the notion that the former is blended with the latter in unbalanced settings. This also demonstrates why a subsequent embedding per cell type population is needed, where the intra-cell-type covariation can be captured more accurately without being tied to cross-cell-type variation. DR-GEM improve cell type annotations and hence this downstream cell-type-specific analyses will be less likely to be contaminated by signals coming from other cell types as misassigned rare cells.

2) Contextualizing DR-GEM within ML theory: DR-GEM leverages a well-established tendency of standard empirical risk minimization (ERM)–based models to under-optimize minority/rare populations in the data. Models trained by minimizing average loss disproportionately prioritize majority groups, because the contribution of any subgroup to the objective function scales with its prevalence. Consequently, errors incurred on rare subpopulations have a smaller influence on the learned representation and are more likely to reside in the tail of the loss distribution. This phenomenon, otherwise termed "minority subgroup bias" or "long-tail error amplification", has been described in existing literature[6–8] on distributional robustness. PCA is a special case of this broader principle. Classical PCA implicitly minimizes the mean squared reconstruction error (MSE) across all samples, that is, the L2 norm of the difference between the original and reconstructed data. As a result, the optimal projection is dominated by directions of variance that explain the majority of the data mass, even when these directions systematically under-represent smaller or structurally distinct subgroups. Prior theoretical and empirical work has shown that PCA can explicitly trade off subgroup-specific reconstruction error to minimize global loss, thereby "sacrificing" minority groups when their variance structure is misaligned with that of the majority[9–12]. We have added the appropriate citations to the revised manuscript on page 5, lines 322-327 to better reference this important theoretical foundation.

3) Additional datasets demonstrate that rare cell types exhibiting high RE values (pages 4-5, lines 146-165; **Methods**, page 46, lines 1,219-1235, new **Supplementary Fig. 2**): We have analyzed two additional scRNA-seq datasets where biologically and clinically important rare cell types were identified: (1) human developing thymus[13] where rare transient progenitor and differentiation-state populations were identified via integrated developmental trajectories, conserved external gene signatures, and independent validation across donors and held-out datasets; and (2) tracheal epithelium of control and mouse models of cystic fibrosis[14], where rare epithelial subtypes were identified via integrated analyses with complementary bulk sequencing, histology, and mouse genotype. The minority/rare cells are enriched in the right tail of reconstruction error distribution in these two datasets (new **Supplementary Fig. 2E-F)**, and in new types of simulated datasets (new **Supplementary Fig. 7B-C**), as we have previously shown across synthetic, scRNA-seq, spatial transcriptomics, imaging mass cytometry proteomics, and Perturb-seq data in diverse biological contexts (**Fig. 1C-E, Supplementary Figs. 2A-D,G-K, 7A**).

3. A major weakness of this manuscript is the lack of comprehensive comparisons with relevant state-of-the-art methods in the field.

In Figure 1, the authors use PCA as the basic dimensionality reduction tool to demonstrate the problem but do not compare it with other non-linear dimensionality reduction or embedding methods specifically designed for single-cell data (such as VAE-based models like scVI). Given that these methods have advantages in modeling the characteristics of single-cell data, re-evaluating the phenomenon of "high RE in rare cells"

using them as a baseline would make the conclusions more convincing. It is also necessary to clarify whether the DR-GEM framework itself is affected by the choice of the underlying dimensionality reduction/reconstruction method.

Other advanced algorithms aimed at improving the detection of rare cell types already exist in the field (e.g., GiniClust). A comprehensive benchmark comparison of DR-GEM with these more challenging state-of-the-art methods is crucial for an objective assessment of its performance and novelty.

We thank the Reviewer for surfacing the need to test DR-GEM with other (including non-linear) dimensionality reduction methods and against other algorithms developed to identify rare cells from single cell data. Below, we describe these new analyses and results.

a) DR-GEM outperforms the standard pipeline also when applied with other dimensionality reduction methods (revised manuscript, page 7 lines 262-268; **Methods**, page 43, lines 1,107-1,117; new **Supplementary Figs. 4-5**): We tested DR-GEM compared to the standard pipeline, where this time, instead of PCA, both frameworks were applied to Synthetic Dataset 1 with three other dimensionality reduction methods: Kernel PCA with polynomial kernel (degree = 2, new **Supplementary Fig. 4**), Latent Dirichlet Allocation (LDA, new **Supplementary Fig. 5A-B**), and the VAE-based method scVI (new **Supplementary Fig. 5C-D**). Despite significant differences between these underlying dimensionality reduction methods, under all methods, the minority/rare cell type exhibits high RE compared to the other cell types. Applying DR-GEM markedly improved the embedding and annotation of the minority/rare cell types across all these methods compared to the standard pipeline.

b) Benchmarking DR-GEM compared to rare cell type detection methods (revised manuscript, pages 12-13, lines 508-541; **Methods**, pages 46-47, lines 1245-1269, page 43, lines 1,089-0,099; revised **Supplementary Table 2**):

We benchmarked DR-GEM against three conceptually diverse end-to-end algorithmic frameworks for rare cell detection and/or cell type annotation: GiniClust3[15] (the most recent of the GiniClust series), scCAD[16], and CIARA[17]. While these methods were tested exclusively on scRNA-seq datasets, here we benchmarked performances across 9 different single cell datasets and 14 rare cell detection tasks (revised **Supplementary Table 2**), spanning diverse biological contexts (human tubo-ovarian cancer, mouse preoptic hypothalamus, genetically perturbed experimental cell lines, mouse models of cystic fibrosis, human developing thymus, human peripheral blood mononuclear cells, and human head and neck cancer) and data types (synthetic simulation, scRNA-seq, spatial transcriptomics, and Perturb-Seq).

DR-GEM outperforms the other three methods across 12 of 14 of the datasets, as quantified via Accuracy, Balanced Accuracy, Sensitivity (True Positive Rate), Specificity (True Negative Rate), balanced clustering metrics, and standard clustering metrics (revised **Supplementary Table 2**). When applied to the PBMC scRNA-seq dataset, scCAD outperformed all other methods by detecting precisely all the rare cells (Platelets), though the performance in this dataset was strong across all the methods tested, even with the standard pipeline with PCA. When applied to the human developing thymus scRNA-seq dataset, CIARA, a method built and tested on developmental datasets, outperformed other methods. However, performance across all other methods in this dataset was also strong.

DR-GEM significantly outperforms the other methods in our synthetic, spatial transcriptomics, and Perturb-seq datasets. Notably, GiniClust3 was not compatible with datasets with fewer than 200 features, precluding results for Synthetic Dataset 1, Synthetic Dataset 2, and the Mouse Preoptic Hypothalamus MERFISH dataset. In the tubo-ovarian cancer SMI dataset and across all Perturb-Seq settings, DR-GEM's Sensitivity for the rare cell type was higher than GiniClust3's. DR-GEM also outperformed GiniClust3 across both balanced clustering and standard clustering metrics across all spatial transcriptomics and Perturb-Seq datasets. scCAD did not detect any rare cells at all in Synthetic Dataset 1, Synthetic Dataset 2, and in 3 Perturb-seq settings (MED19i, CASP8A2i, and NRDE2i vs NTC). Moreover, scCAD's Sensitivity in detecting the ground truth rare cells is 0 across the remaining spatial transcriptomics and Perturb-Seq datasets, despite the method identifying some rare cells. CIARA did not detect any rare cells across all 6 Perturb-Seq datasets.

In summary, DR-GEM is non-inferior to GiniClust3, scCAD, and CIARA methods for the specific tasks of rare cell detection and annotations in scRNA-seq data, and significantly outperforms these methods when applied to synthetic, spatial transcriptomics, and Perturb-seq datasets. Lastly, we note that DR-GEM objective is to obtain better data representations, which improves down-stream tasks such as rare cell type detection. Yet, beyond rare-cell detection, DR-GEM derives more biologically meaningful lower-dimensional embeddings, clustering, annotations for the whole dataset, as we show analytically and empirically throughout the revised manuscript.

4. An important frontier in current single-cell biology is the exploratory analysis of large-scale cell atlas datasets to discover novel, functionally important rare cell subpopulations. However, all experiments in this manuscript are limited to validating the ability to recover "known" rare cell types. We strongly recommend that the authors apply DR-GEM to one or more large, incompletely annotated cell atlas datasets and attempt to identify new candidate rare cell types that can be validated by marker genes. This would greatly enhance the practical application value and impact of the method.

We thank the Reviewer for surfacing the need to demonstrate DR-GEM's performance for discovery of previously undetected rare cells. To address this comment, we applied DR-GEM to SMI data of malignant cells from primary adnexal tumors in tubo-ovarian cancer, spanning 185,071 high quality single cells representing tumors from 43 patients. Twelve of these patients were underrepresented in this data (<1% representation for each patient). We refer to these as "underrepresented patients".

First, DR-GEM outperformed the standard pipeline in capturing somatic inter-patient heterogeneity. Recapitulating the previously documented pattern that malignant cells tend to cluster by patient, the embedding with the standard pipeline yielded a total of 44 patient-specific clusters that mapped to 34 out of the 43 patients. The standard pipeline analysis may on the surface suggest that the remaining 9 patients do not have a unique malignant cell state signature. However, all "non-unique" patients were also underrepresented (new **Figs. 7A-B**), indicating that these may result from sampling-bias. In contrast, with DR-GEM, 39 out of the 43 patients mapped to a patient-specific cluster, including 8 out of the 12 underrepresented patients (new **Fig. 7C**), demonstrating the ability of DR-GEM to mitigate underrepresentation both at the cell and patient level.

Second, DR-GEM discovered a new *de novo* cluster corresponding to a spatially and morphologically distinct subclone of malignant cells within a patient's tumor. More specifically, DR-GEM uncovered a new cluster formed from 543 (0.29%) cells from patient 1 (cluster 49 in new **Fig. 7C**), thereby detecting 3 clusters within HGSC1 with DR-GEM (clusters 1, 2, and 49 in new **Fig. 7D-E**) versus 2 clusters (clusters 1 and 2) with the standard pipeline. Spatial analysis and independent review by a board-certified pathologist determined that cluster 49 maps to a morphologically distinct region of the tumor tissue.

These new analyses and findings are described and visualized in the revised manuscript (page 10, lines 388-407, new **Fig. 7**) and **Methods** (page 45, lines 1,180-1,186).

Furthermore, the simulation and real-data test scenarios in this study mostly involve only one major rare cell type. However, in real complex tissues (such as the tumor microenvironment or developing organs), there are often multiple rare cell populations with different abundances. Whether DR-GEM's initialization and balanced sampling phases can handle multiple high-RE rare populations simultaneously is an unverified key question. We suggest the authors design simulation experiments involving multiple rare cell types to evaluate the performance and robustness of DR-GEM in such more realistic and complex scenarios.

To address the Reviewer's comment, we tested DR-GEM in simulated datasets with multiple minority/rare cells.

First, we show that the central hypothesis guiding DR-GEM's design holds also in more complex settings with multiple types of rare cells. Simulating a total of 500 synthetic datasets with either 2, 5, 10, 20, or 100 unique minority cell types (new **Supplementary Fig. 8**), we show that the mean reconstruction error for each unique synthetic cell type is systematically higher for all minority cell types (n = 50 per population) compared with majority cell types (defined as $n \geq 4000$ in each population). Testing this behavior in a real-world dataset, the scRNA-seq data of tracheal epithelium[14] includes 4 minority/rare cell types. All 4 are most enriched in the right tail of the reconstruction error distribution (new **Supplementary Fig. 2F**).

Second, we tested DR-GEM in an additional synthetic dataset (Synthetic Dataset 3), which simulates imbalanced labels with 5 distinct minority/rare cell types. DR-GEM outperformed the standard pipeline in Synthetic Dataset 3 (new **Fig. 5C-D**). It equalized RE across the synthetic cell types and clustered all 5 rare cell types as their unique separate cluster, compared to the standard pipeline where the rare cell types did not form their own clusters and instead each of the rare cell types was scattered across the other clusters. DR-GEM correctly annotated 90-98% of each of the rare synthetic cell types, compared to 0% with the standard pipeline.

These new analyses and results are described in the revised manuscript (page 8, lines 312-329, new **Fig. 5C-D,** new **Supplementary Fig. 8**) and **Methods** (pages 44-45, lines 1,149-1,170).

5.The validation on real datasets relies on the annotations from the original studies as the "gold standard." This introduces a potential risk of circular reasoning, as the original annotations themselves may have been obtained through similar computational pipelines. We suggest the authors discuss this limitation and explore other validation strategies.

This is indeed a limitation that we have previously attempted to mitigate by using Perturb-seq data (where ground truth annotations are based on sgRNA detection) and data simulations. We have now also benchmarked DR-GEM in improving the representation of underrepresented patients and recovering histological information using the SMI dataset profiling tubo-ovarian tumors (new **Fig. 7**). As described in response to comment #4 above, DR-GEM: (1) outperformed the standard pipeline in capturing inter-patient heterogeneity (new **Fig. 7A-C**), and (2) discovered a cluster that was missed by the standard pipeline and marks spatially and morphologically distinct subclone of malignant cells, as revealed by projecting the cells to their spatial coordinates and independent annotations of a board-certified pathologist reviewing the matching H&E (new **Fig. 7D-E**).

We revised the manuscript to discuss and further address these limitations and present these new analyses and results (page 4-5, lines 146-164, page 10, lines 388-407, new **Fig. 7,** new **Supplementary Fig. 2E-F**) and **Methods** (page 45, lines 1,180-1,186).

6. DR-GEM introduces several hyperparameters that could have a significant impact on the results. The manuscript lacks detailed guidance on the selection of these parameters and a systematic sensitivity analysis. We recommend that the authors provide a thorough analysis showing the impact of different choices within a reasonable parameter range on the final clustering results and rare cell identification performance to demonstrate the method's stability and ease of use.

<u>Number of dimensions for dimensionality reduction:</u> We have performed a sensitivity analysis across 500 datasets to examine the relationship between the number of PCs used and the RE bias (new **Supplementary Fig. 10A**). The minority/rare cell types exhibit higher RE compared to the largest cell type population across 5, 10, 20, 50, and 100 PCs. Using more PCs decreases the enrichment of the minority/rare cell population in the top 10% of the RE distribution in Synthetic Dataset 1, though the minority/rare cell population is still disproportionately enriched in the tail even when using 100 PCs (new **Supplementary Fig. 10B**). To select how many dimensions to reduce data to, we recommend using tools most appropriate for each dataset at the user's discretion (e.g., elbow/scree plot or the Jack straw method).

<u>Selecting β in Phase I of DR-GEM:</u> The β hyperparameter defines the proportion of the right tail to use to "refocus" Phase I's embeddings on. Across multiple synthetic datasets with and without technical noise (**Fig. 1C**, new **Supplementary Fig. 7**) and real-world datasets (**Fig. 1D-E**, new **Supplementary Fig. 2),** the smaller the β, the higher the enrichment of the minority/cell type. Applying DR-GEM to Synthetic Dataset 1 whilst varying only the β hyperparameter shows that smaller values are better at improve the detection of the minority/rare cells (new **Supplementary Fig. 10C**). With β = 0.5 and β = 0.8 DR-GEM did not correctly identify any of the minority/rare cells. We have set β = 0.05 as the default in the DR-GEM package.

<u>Selecting number of iterations (*r*) in DR-GEM's Phase II</u>: The optimal *r* depends on the degree of latent class imbalance, level of ambient RNA and other sources of noise, as we now show by tracking Phase II convergence across iterations and datasets (new **Supplementary Fig. 11**). For example, in the Perturb-seq data, there is a

steady increase in prediction accuracy with each iteration, until the prediction accuracy plateau above ~150 iterations. As expected, the number of iterations required to sample each cell type population scales with the size of the population. To support users in selecting the number of Phase II iterations to reach convergence, we have now added a new optional functionality where:

(1) DR-GEM assesses every *r1* iterations after all cells have been sampled at least 5 times, how much the annotations change for each final assigned label (δ_k). If the change of proportion of assignments across all final predicted annotations ($\delta_1 ... \delta_K$) are below δ_{target} (default=0.05), DR-GEM can terminate earlier than initially specified by *r*.
(2) A user can add *r2* additional iterations without repeating the full run to reach convergence.

Confidence level (*t*) to select the cells for the reference map in DR-GEM's Phase III: Applying the confidence level *t* for selecting cells in the reference map occurs after DR-GEM already finalizes the cell type assignments and therefore selection of *t* does not impact the rare cell detection task. However, *t* does impact the fairness-aware downstream embedding, and we thus revised the manuscript to provide additional guidance here for choosing a reasonable *t*. The distribution of s_i is data dependent. We demonstrate in several datasets that lower scores are associated with (i) ambient RNA signal bleeding in spatial single cell data (new **Supplementary Fig. 12A**), (ii) cell doublets or multiplets in droplet-based data (**Fig. 4B-H**), or (iii) true intertypic cells in single cell datasets with more continuous structure (new **Supplementary Fig. 12B**). The most ideal scenario is for cells selected for the reference map to (1) have highest confidence scores as possible and (2) for final cell populations to be as balanced in frequency as possible. The constraint is that higher levels of *t* to optimize (1) could exclude cells such that there are insufficient numbers of cells in each cell type population to subsample and achieve goal (2). We thus revised DR-GEM to include output plots that visualizes s_i distributions across the final clusters and notify the user in cases where a specific cluster has significantly lower s_i values.

These guidelines, new analyses and features are described in the revised manuscript (page 6, lines 223-225, 240-245), new **Supplementary Figs. 10-12**, and **Methods** (page 42, lines 1,052-1,061, 1,070-1,072).

Minor Concerns:

1.When using non-linear dimensionality reduction methods (like Variational Autoencoders, VAEs) instead of PCA, their reconstruction error (RE) calculation and interpretation differ from PCA. How does the DR-GEM framework standardize or adjust the definition of RE to accommodate different dimensionality reduction algorithms, ensuring its effectiveness as a "hard example" metric is comparable across different algorithms?

In the case of non-linear methods, if there is a "decoder" or "projection" that maps the reduced dimensions to the input feature space, one can use the mean squared error (MSE) for each sample as the RE, as we have done for PCA and now also in applications of DR-GEM with non-linear polynomial kernel PCA (new **Supplementary Fig. 4**). In the case of probabilistic generative models such as LDA and scVI (a VAE), the equivalent of RE is the negative log likelihood per sample[3,4]. We utilized the negative log likelihood per sample as the RE when applying DR-GEM with LDA[3] and scVI[4], demonstrating DR-GEM's performance on embedding and downstream annotations in this setting as well (new **Supplementary Fig. 5**).

2. In Phase II, when the number of clusters generated in an iteration is inconsistent with the number of initial "weak label" clusters (e.g., due to dynamic changes in clustering resolution), how does the Kuhn-Munkres alignment strategy handle this? Is there a mechanism for merging or splitting clusters?

In Phase II, the Kuhn–Munkres (Hungarian) does not require the number of clusters to match the number of initial weak-label clusters. When more clusters are produced than the number weak labels, the optimal assignment *implicitly* merges multiple clusters by mapping them to the same weak-label cluster using the scoring matrix/bipartite graph. When fewer clusters are produced than the number of weak labels, the algorithm will match each cluster to its most similar weak label, such that one or more of the weak labels will not be assigned to a new cluster. We revised the **Methods** to explain and clarify this point (page 41, lines 1,028-1,031).

3. The final step of DR-GEM is to project all cells onto the reference embedding space constructed from high-confidence cells. What is the main consideration behind adopting this projection method rather than directly using the original embeddings of all cells combined with consensus clustering labels for visualization? Could this approach potentially lose unique variation information from cells not selected for the reference set?

Using all the cells with to generate the final embedding suffers from the same problems that drove us to develop DR-GEM, that is, the distortion of distance definition, inadequate representation of clusters and inter-cluster variation, and propensity to fit to technical noise. Throughout the study we show, both analytically and empirically that these are indeed problems in the original embedding.

To address the Reviewers comment, we added to DR-GEM another output – the original embedding of all cells combined with the consensus clustering labels and confidence scores for visualization and interpretation of DR-GEM's outputs, as described in the last section of our tutorial at https://htmlpreview.github.io/?https://github.com/Jerby-Lab/drgem/blob/main/vignettes/tutorial_tubo-ovarian.html.

We acknowledge the reviewer's important observation: a low cluster assignment confidence score can also reflect biologically meaningful variation—particularly of differentiation or other intermediate states in cell state transitions. Thus, in biological contexts where such transitions are expected or in datasets and data types where ambient RNA is less of a concern, we recommend to further examine the cells with a low confidence score and/or use a lower cutoff on the confidence score, which will retain inter-state cells.

These additions and guidelines are now included in the revised manuscript (page 6, lines 240-245).

4. In a cell population that is highly heterogeneous but numerically dominant (such as tumor cells with high intrinsic heterogeneity), outlier cells within it may also exhibit high RE. In this case, would DR-GEM's initialization step mistakenly focus on this internal heterogeneity rather than on a truly independent rare cell type?

This is indeed another important point. As described below, DR-GEM's definitions of distances are completely data-driven and not bound to prior notions of how the data should be split to groups, though "annotations-in-the-loop" provides a mechanism to control/reorient this if deemed.

More specifically, DR-GEM can be interpreted through the lens of hierarchical clustering, as it aims to obtain an accurate clustering of cells at the highest level of the dendrogram (i.e., first set of splits, closer to the root of the tree). These data splits can reflect different cell type identities, different genetic backgrounds, etc. Accuracy in this context can be examined based on the following property: if the distance between cells A and B is d, and cells A and B are assigned to different clusters, then any other cell C that is of distance d from both A and B should form a separate cluster. As we show, the standard pipeline does not fulfill this requirement when C is a rare cell.

If the data consists of different cell types, the cells are likely to first cluster by cell type, but this is not necessarily the case in all datasets. The definition of a "group" is data driven and can reflect different cell types, genetically distinct cancer cells, etc., as we show in response to major comment #4 above. In settings where there is a mega-group (e.g., malignant cells) consisting of multiple groups (e.g., malignant cells from different patients or clones) that the user is not interested in, the "annotations-in-the-loop" mechanism allows users to constrain or merge such variation, ensuring DR-GEM prioritizes user-defined notions of relevant variation to focus on. We now clarify this in the revised manuscript and explicitly guide users on leveraging "annotations-in-the-loop" when analyzing cancer or other heterogeneous cell populations.

These new analyses, guidelines, and results are described and visualized in the revised manuscript (page 2, lines 111-114, page 9, lines 337-340; page 10, lines 388-407; **Fig. 7**) and **Methods** (page 45, lines 1,180-1,186).

5. The simulated data used in the manuscript are mainly based on discrete cell types with clear boundaries. However, many biological processes manifest as continuous transcriptional trajectories. DR-GEM relies on

discrete "weak labels" for balanced sampling, and this mechanism may face challenges when dealing with continuous data structures. We suggest the authors to discuss or experimentally validate their method's potential performance on such data.

The datasets used were simulated or selected to reflect underlying discrete structure as DR-GEM was designed for the task of embedding and annotating discrete groups with latent class imbalance. To address the Reviewer's comment, we applied DR-GEM scRNA-seq dataset profiling thymic epithelial cells (TECs) of the human developing thymus[13] where Cycling TECs were found to bifurcate at the mcTEC (intertypic cell type with both medullary and cortical TEC signatures) stage to develop to either cTEC or mTEC 2 state:

1. Cycling TEC → mcTEC → cTEC (cortical TEC)
2. Cycling TEC → mcTEC → mTEC 1 (medullary TEC 1) → mTEC 2

DR-GEM correctly matched the majority of each of the published cell types, such that incorrect labeling was primarily to the preceding or proceeding state and was matched with lower confidence scores (new **Supplementary Fig. 12B**). In this continuous data structure, lower scores may reflect the true "in between" single cell expression of these cells despite both the original authors and DR-GEM forcing each cell into a discrete label. In practice, we recommend that unless the goal of researchers is to robustly discretize otherwise suspected continuous data, that researchers utilize tools specifically for trajectory analysis rather than or in addition to DR-GEM (revised manuscript, page 6, lines 24-245, page 14, lines 579-581).

6. The iterative process of DR-GEM seems focused on optimizing and stabilizing existing cluster assignments. If a genuinely existing rare cell type fails to form an independent cluster (even a small one) in the initial clustering, can DR-GEM "create" this new cluster in subsequent steps? Does this, to some extent, limit the algorithm's ability to discover entirely new, unforeseen cell types?

In the default pipeline, if "new" clusters are generated in Phase II, they are matched by the Kuhn-Munkres algorithm to the available annotations created in Phase I, thus indeed limiting discovering unforeseen cell types in Phase II. In the "annotations-in-the-loop" version, however, new clusters can be "created" in Phase II and either matched to one of the input signatures or annotated as "Undetermined". Cells that are repeatedly mapped to the "Undetermined" label can still get a high confidence score and be selected and used in Phase III to generate the reference map. We recommend users of DR-GEM with "annotation-in-the-loop" to investigate these cells to see if they may represent unforeseen new cell types. We revised the **Methods** to explain the difference between the two DR-GEM modes in this regard (page 28, lines 1047-1,051).

7.We suggest the authors provide a more detailed explanation of what types of cells generally constitute those with high confidence scores, and whether this score is related to the abundance or heterogeneity of specific cell types.

High confidence cells are cells that do not "switch" clusters often across DR-GEM's Phase II. We hypothesize that the "switching" clusters can be from cells that have a mix of gene signature expression that reflect (i) ambient RNA signal bleeding in spatial single cell data, (ii) cell doublets or multiplets in droplet-based data, or (iii) true intertypic cells in single cell datasets with more continuous structure. (i) is manifested in the tubo-ovarian cancer dataset, where cells in *less* heterogeneous tissue regions tend to have higher confidence scores (modal score = 1), than those in *more* heterogeneous tissue regions (Kruskal-Wallis p-value = 1.65e-28, new **Supplementary Fig. 12A**); (ii) is manifested in simulations of cell multiplets in Synthetic Dataset 2, where the confidence scores are inversely associated with cell contamination (**Fig. 4D-G**); (iii) is manifested in the human developing thymus dataset, where discordantly labeled cells with low confidence scores may represent a true continuum of gene expression (new **Supplementary Fig. 12B**).

8.To more intuitively demonstrate the working mechanism of DR-GEM, the authors could consider adding a supplementary figure that visually illustrates the evolution of a rare cell cluster from its initial "weak label"

to the final consensus label (e.g., the change in the number of cells it contains and its purity). This would also help to illustrate the necessity of the consensus clustering step.

We have added a visual representation of evolution of all cell populations across iterations in Phase II of DR-GEM for Synthetic Dataset 1, Synthetic Dataset 2, the tubo-ovarian cancer SMI dataset, and the Perturb-Seq dataset of *PTPN1*i and non-targeting controls (new **Supplementary Fig. 11**). We see that Phase II is particularly important for datasets that have known or suspected noise from ambient RNA, where the risk of "switching" of cluster labels: e.g. in Synthetic Dataset 2 (new **Supplementary Fig. 11B**), particularly for Cell Type 1 and the real-world tubo-ovarian cancer SMI dataset (new **Supplementary Fig. 11C**), particularly for B cells.

Typographical Errors:

In the caption of Figure 1B, "synthetic" is misspelled as "dynthetic".

Thank you, we have fixed this typographical error in **Fig. 1B**.

References

1. Yeh CY, Aguirre K, Laveroni O, Kim S, Wang A, Liang B, et al. Mapping spatial organization and genetic cell-state regulators to target immune evasion in ovarian cancer. Nat Immunol. 2024 Aug 23;1–16. doi:10.1038/s41590-024-01943-5
2. Moffitt JR, Bambah-Mukku D, Eichhorn SW, Vaughn E, Shekhar K, Perez JD, et al. Molecular, spatial, and functional single-cell profiling of the hypothalamic preoptic region. Science. 2018 Nov 16;362(6416):eaau5324. doi:10.1126/science.aau5324
3. Blei D, Ng A, Jordan M. Latent Dirichlet Allocation. In: Advances in Neural Information Processing Systems [Internet]. MIT Press; 2001 [cited 2026 Jan 2]. Available from: https://proceedings.neurips.cc/paper/2001/hash/296472c9542ad4d4788d543508116cbc-Abstract.html
4. Lopez R, Regier J, Cole MB, Jordan MI, Yosef N. Deep generative modeling for single-cell transcriptomics. Nat Methods. 2018 Dec;15(12):1053–8. doi:10.1038/s41592-018-0229-2
5. Jackson HW, Fischer JR, Zanotelli VRT, Ali HR, Mechera R, Soysal SD, et al. The single-cell pathology landscape of breast cancer. Nature. 2020 Feb;578(7796):615–20. doi:10.1038/s41586-019-1876-x
6. Sagawa S, Koh PW, Hashimoto TB, Liang P. Distributionally Robust Neural Networks for Group Shifts: On the Importance of Regularization for Worst-Case Generalization [Internet]. arXiv; 2020 [cited 2025 Apr 16]. Available from: http://arxiv.org/abs/1911.08731 doi:10.48550/arXiv.1911.08731
7. Duchi J, Namkoong H. Learning Models with Uniform Performance via Distributionally Robust Optimization [Internet]. arXiv; 2020 [cited 2024 Sep 17]. Available from: http://arxiv.org/abs/1810.08750 doi:10.48550/arXiv.1810.08750
8. Duchi J, Hashimoto T, Namkoong H. Distributionally Robust Losses for Latent Covariate Mixtures [Internet]. arXiv; 2022 [cited 2026 Jan 2]. Available from: http://arxiv.org/abs/2007.13982 doi:10.48550/arXiv.2007.13982
9. Samadi S, Tantipongpipat U, Morgenstern JH, Singh M, Vempala S. The Price of Fair PCA: One Extra dimension. In: Advances in Neural Information Processing Systems [Internet]. Curran Associates, Inc.; 2018 [cited 2024 Jun 18]. Available from: https://papers.nips.cc/paper_files/paper/2018/hash/cc4af25fa9d2d5c953496579b75f6f6c-Abstract.html
10. Tantipongpipat U, Samadi S, Singh M, Morgenstern J, Vempala S. Multi-Criteria Dimensionality Reduction with Applications to Fairness [Internet]. arXiv; 2020 [cited 2024 Jun 21]. Available from: http://arxiv.org/abs/1902.11281 doi:10.48550/arXiv.1902.11281
11. Kamani MM, Haddadpour F, Forsati R, Mahdavi M. arXiv.org [Internet]. 2019 [cited 2024 Jun 18]. Efficient Fair Principal Component Analysis. Available from: https://arxiv.org/abs/1911.04931v2
12. Rathore V, Manwani N. Achieving Fair PCA Using Joint Eigenvalue Decomposition [Internet]. arXiv; 2025 [cited 2026 Jan 2]. Available from: http://arxiv.org/abs/2502.16933 doi:10.48550/arXiv.2502.16933

13. Mohammed A, Wang W, Arreola M, Solomon BD, Slepicka PF, Hubka KM, et al. Distinct type I and II interferon responses direct cortical and medullary thymic epithelial cell development. Science Immunology. 2025 May 2;10(107):eado4720. doi:10.1126/sciimmunol.ado4720
14. Montoro DT, Haber AL, Biton M, Vinarsky V, Lin B, Birket SE, et al. A revised airway epithelial hierarchy includes CFTR-expressing ionocytes. Nature. 2018 Aug;560(7718):319–24. doi:10.1038/s41586-018-0393-7
15. Dong R, Yuan GC. GiniClust3: a fast and memory-efficient tool for rare cell type identification. BMC Bioinformatics. 2020 Apr 25;21(1):158. doi:10.1186/s12859-020-3482-1
16. Xu Y, Wang S, Feng Q, Xia J, Li Y, Li HD, et al. scCAD: Cluster decomposition-based anomaly detection for rare cell identification in single-cell expression data. Nat Commun. 2024 Aug 31;15(1):7561. doi:10.1038/s41467-024-51891-9
17. Lubatti G, Stock M, Iturbide A, Ruiz Tejada Segura ML, Riepl M, Tyser RCV, et al. CIARA: a cluster-independent algorithm for identifying markers of rare cell types from single-cell sequencing data. Development. 2023 Jun 8;150(11):dev201264. doi:10.1242/dev.201264

We thank the reviewers for the thoughtful review and insightful feedback. We are pleased that the reviewers appreciate the revisions and for surfacing opportunities for further improvement. As described in detail in our point-by-point response below, we addressed the Reviewers' comments by providing analytical proof of DR-GEM's convergence and error bounds and adding new commentary and clarifications in the revised text.

In the manuscript files, revised sections are marked in blue.

Point-by-point response to Reviewer comments

Reviewer #1 (Remarks to the Author):

Authors have addressed most of my comments, which is good and I appreciate their efforts. However, the mathematical guarantees of their methods are still weak as I mentioned in previous comment 5/6. It would be better for the author to give a better illustration.

We thank the Reviewer for emphasizing the need for convergence proof and error bounds for Phase II of the algorithm, which we have now added to the revised manuscript (pages 27-28, lines 1084-1111; new **Supplementary Fig. 13**), as described below.

Point 5, especially point 6. The author provides empirical convergence analysis, an auto-convergence mechanism, and a confidence score, but doesn't actually provide a formal convergence proof or error bounds. Therefore, this point should be further clarified.

We revised the manuscript to provide a formal proof and derive mathematical guarantees of DR-GEM Phase II convergence and error bounds (revised **Methods**; pages 27-28, lines 1084-1111).

DR-GEM Phase II convergence and error bounds

Given the input data, let S include all the data subsets consistent with the sampling procedure of Phase II, such that the total size of S is N. Let c_{ij} be the annotation of cell i in a subset $j \in S$. The target annotation of cell i is defined as the cell's most frequent annotation across all N subsets:

$$c_i^* = argmax_k \sum_{j=1}^{N} 1[c_{ij} = k]$$

Below, we show that this is the annotation that Phase II converges to, as a function of γ_i, defined as

$$\gamma_i = \sum_{j=1}^{N} \frac{1[c_{ij} = c_i^*]}{N}$$

The confidence score s_i is the estimate of γ_i based on $S_r \subset S$, where $|S_r| = r$.

Let $\hat{c_i}$ be the annotation that Phase II converges to within r iterations. The probability of correct labeling across r iterations thus follows a binomial distribution:

$$\Pr(\hat{c_i} = c_i^* |\, s_i \geq s_i^*) = \frac{\Pr(\hat{c_i} = c_i^*, s_i \geq s_i^*)}{\Pr(s_i \geq s_i^*)} \geq \Pr(\hat{c_i} = c_i^*, s_i \geq s_i^*) = \sum_{n=\lceil r*s_i^* \rceil}^{r} \binom{r}{n} \gamma_i^n (1-\gamma_i)^{r-n}$$

Thus, within 1, 3, 5, 7, 9, 17, 29, 67, and 269 iterations, the probability of an error is ≤ 5% for cells with γ_i = 0.95, 0.9, 0.85, 0.8, 0.75, 0.7, 0.65, 0.6, and 0.55, respectively (new **Supplementary Fig. 13A**).

The Odds Ratio (OR) between the probability of correct and incorrect labeling of cell i increases as a function of s_i.

$$OR_i = \frac{\Pr(\hat{c_i} = c_i^* |\, s_i > \hat{s_i} > 0.5)}{\Pr(\hat{c_i} \neq c_i^* |\, s_i > \hat{s_i} > 0.5)} = \frac{\Pr(\hat{c_i} = c_i^*, s_i > \hat{s_i} > 0.5)}{\Pr(\hat{c_i} \neq c_i^*, s_i > \hat{s_i} > 0.5)} \geq \frac{\sum_{n=\lceil r*\hat{s_i} \rceil}^{r} \binom{r}{n} \gamma_i^n (1-\gamma_i)^{r-n}}{\sum_{n=\lceil r*\hat{s_i} \rceil}^{r} \binom{r}{n} (1-\gamma_i)^n {\gamma_i}^{r-n}}$$

Thus, the cells used for the reference map in Phase III are less likely to be mislabeled.

The Hoeffding's inequality for Bernoulli random variables provides an exponential bound on the probability that s_i deviates from γ_i by a specific margin t, as a function of r (new **Supplementary Fig. 13B**).

$$\Pr(\, s_i - \gamma_i \geq t) \leq e^{-2rt^2}$$

$$\Pr(\gamma_i > s_i - \, t) \geq 1 - e^{-2rt^2}$$

Thus, if selecting cells with $s_i \geq 0.95$ for the reference maps, as done here, it is estimated that within 17 iterations per cell, 74.3% of the cells selected for the reference map will have a $\gamma_i > 0.75$ and thus correctly annotated with an error rate ≤ 5%, such that considering all the reference cells, the total error rate is ≤ 15%.

$$\Pr(\gamma_i \geq 0.75 \mid s_i \geq 0.95, r \geq 17) \geq 1 - e^{-34*0.2^2} = 0.743$$

Complementing these convergence and error bounds, we have demonstrated, both analytically and empirically, that balanced subsampling improves the embedding for both rare and abundant cells by mitigating distortions in distance definitions caused by class imbalance. PCA is a particularly interesting case in which we show analytically that feature covariance is distorted by class imbalance (pages 20-21, lines 796-844, in the revised manuscript).

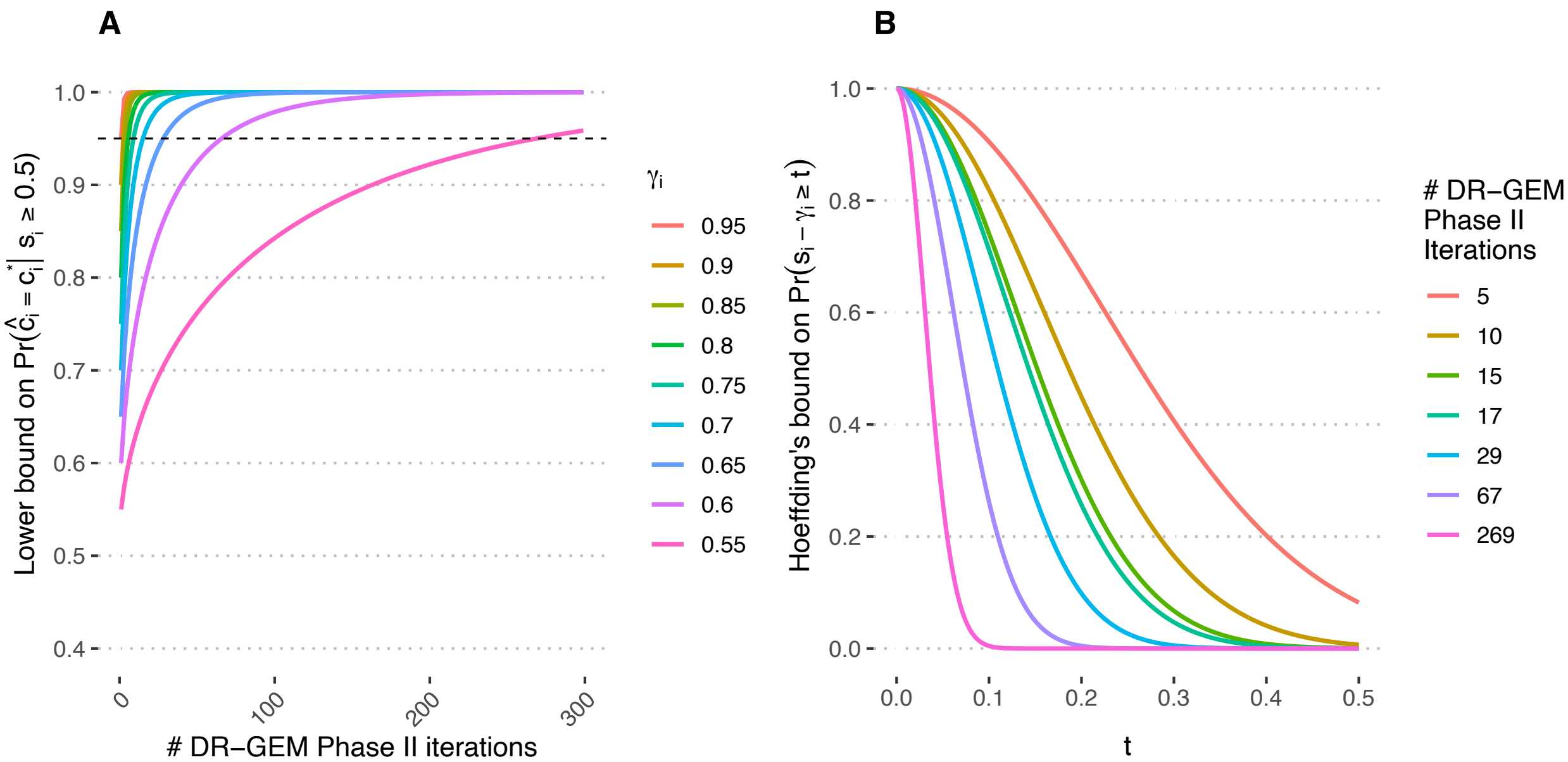

Supplementary Figure 13. Phase II convergence rate and error bounds. (A) The lower bound on the probability that a cell with $s_i^* = 0.5$ is labeled correctly (*y*-axis) as a function of the number of iterations ran (*x*-axis), with each line representing a different γ_i value [0.55, 0.95]. **(B)** The Hoeffding's inequality bounds of the probability (*y*-axis) that the confidence score s_i deviates from the γ_i by t (*x*-axis) as a function of the number of Phase II iterations (r, lines).

Reviewer #1 (Remarks on code availability):

The code is good now.

We thank the reviewer for their feedback on the code.

Reviewer #2 (Remarks to the Author):

I co-reviewed this manuscript with one of the reviewers who provided the listed reports. This is part of the Nature Communications initiative to facilitate training in peer review and to provide appropriate recognition for Early Career Researchers who co-review manuscripts.

We thank the Reviewer for co-reviewing our manuscript and providing valuable feedback.

Reviewer #3 (Remarks to the Author):

The revised manuscript and the supplementary materials are comprehensive, and the authors have rigorously addressed the concerns raised in the first round of review. We have no remaining major issues regarding the main content of the manuscript. We recommend that the authors briefly discuss the following minor points in the Discussion or Supplementary Information.

We are pleased that the Reviewer found the revisions satisfactory and thank them for their feedback and comments that have helped us improve the study.

1. The new auto-convergence feature in Phase II is a practical addition. However, as spatial transcriptomics datasets continue to grow rapidly (such as merged cohorts with millions of cells or Visium HD data), the repeated dimensionality reduction and matching steps will still take a heavy toll on memory and runtime. To help users manage VRAM and computing bottlenecks, we suggest noting DR-GEM's resource usage for large-scale data or future GPU optimization plans in the Supplementary Information.

We thank the Reviewer for raising the need to address these important aspects of memory and runtime, which are undoubtedly critical for scalability and usability. We have added a note on computational resource usage and scalability (**Supplementary Information**, page 1; revised Discussion, page 14, lines 594-599), a table summarizing DR-GEM computational resource usage when applied to three representative datasets (new **Supplementary Table 4**), as well as analytical convergence and error bounds to help users make informed decisions about the number of iterations needed and resource allocation (**Methods**, page 27-28, lines 1084-1111). The analytical convergence and error bounds are outlined in response to Reviewer #1 above.

The Supplementary Note and Table are provided below.

DR-GEM runtime, memory usage, and practical strategies for resource-limited settings

DR-GEM runtime scales primarily with the number of Phase II iterations (r) and the average number of cells per iteration (**Supplementary Table 4**). As datasets increase in scale in merged cohorts and under high resolution platforms, repeated dimensionality reduction and label-matching steps may create practical bottlenecks.

For large-scale datasets, it is recommended to run Phase II with single runs executed in parallel, in batches, to activate the auto-convergence mode between batches. The analytical convergence and error bounds are provided to help users make informed decisions about the number of iterations needed for a given dataset, as well as general guidelines demonstrating that 17 iterations per cell is sufficient to reach an 85% probability for correct annotations among reference cells ($s_i \geq 0.95$; **Methods**).

For exceptionally large datasets or limited resources we recommend running Phase II on a subsample of the data. More specifically, start by applying Phase I to the entire data to obtain the initial embedding and weak cluster annotation for all cells. Next, based on the initial embedding, obtain a balanced subsample of the data to be provided as input for Phase II, where only clusters with a large number of cells will be subsampled, thus avoiding subsampling cells which are rare or underrepresented to begin with. Lastly, once the DR-GEM reference embedding is obtained for this balanced subset in Phases II and III, the remaining cells can be projected onto it in Phase III.

Additional practical strategies for reducing memory and runtime include limiting the runs to highly variable features (or any other feature selection method most relevant to study system) based on the balanced embedding of Phase I, adjusting the per-cluster sampling cap, preserving sparse matrix representations where possible, and, as outlined above, parallelization of Phase II, and using the auto-convergence criterion to stop Phase II once consensus annotations stabilize.

DR-GEM Phase II is currently implemented with parallelized CPU execution across balanced resampling runs and can be further parallelized on high performance computing clusters. Future implementations may incorporate GPU-accelerated implementation of common dimensionality reduction methods, graph construction, and clustering operations to improve throughput, while preserving the underlying DR-GEM algorithm. These GPU-based optimizations can be implemented alongside future developments in incorporating online-learning for Phase II of DR-GEM as described in the Discussion.

Supplementary Table 4: DR-GEM computational resource usage for representative datasets

Dataset name	# Cells	# Features	# Phase II Iterations	Size of Phase II sub-samples	CPU cores	Peak RAM	Wall time
Synthetic Dataset 2	21550	200	100	4041	10	00.40 GB	0:06:26
Tubo-Ovarian Dataset (SMI)	82808	960	500	3460	10	07.23 GB	4:12:14
Tubo-Ovarian Dataset (MERFISH)*	425578	140	800	15000	4 batches x 16 cores	25.05 GB total	4:30:24

*Run on high-performance computing cluster with "Annotations in the Loop" mode

2. One interesting functionality is the "annotations-in-the-loop" mode for label mapping. How does the model behave when the user-provided signature dictionary is incomplete? Specifically, is there a risk that this mode might force unknown rare cells sharing basic lineage markers into existing categories? A brief discussion on the algorithm's ability to robustly retain such cells as "Undetermined" would be valuable to ensure that novel subpopulations are not inadvertently masked.

We thank the Reviewer for surfacing the need to further discuss the "annotations-in-the-loop". The following paragraph was added to the Discussion section (page 14, lines 576-586):

"DR-GEM "annotations-in-the loop" mode can be used to further orient the model to biological variation of interest. In "annotations-in-the loop" mode the signature dictionary can impact the output, and it is thus recommended to match the granularity of the signatures to the annotation level of interest (e.g., cell type, subtype, state). Importantly, as the criterion for assigning a cluster to a signature is relatively stringent, the risk that rare cells will be forced to predefined categories that do not adequately represent them is low. A cluster that does not meet the assignment criteria for any of the signatures is labeled as "Undetermined". Cells that are repeatedly labeled as "Undetermined" get a high confidence score and are hence likely to be included in the final reference map."

3. The authors suggest using the "annotations-in-the-loop" mode to manage the massive internal heterogeneity of dominant "mega-groups" like malignant cells. If the initial splits predominantly capture the intra-tumor heterogeneity of a mega-group, is there a risk that rare microenvironmental cells are overlooked?

Yes, malignant cells that reflect unique microenvironments as "microenvironmental cells", can be overlooked if using the "annotations-in-the-loop" mode with cell type signatures. To clarify this we revised the Discussion to note that (page 14-15, lines 604-609): "If a dataset consists of a heterogenous mega-group of cells, it is recommended to use annotations-in-the-loop with a signature of the mega-group as the first step to identify these cells and then apply DR-GEM only to the meta-group to identify the intra-group clusters, as done here for malignant cells. Future developments of DR-GEM could benefit from automation of these nested modes for end-to-end embedding, clustering, and annotations across cluster levels."

We also revised the Discussion (page 14, lines 576-579) to note that: "DR-GEM "annotations-in-the loop" mode can be used to further orient the model to biological variation of interest. In "annotations-in-the loop" mode the

signature dictionary can impact the output, and it is thus recommended to match the granularity of the signatures to the annotation level of interest (e.g., cell type, subtype, state).".

If the Reviewer is referring to non-malignant cells as "microenvironmental cells", then these cells are more likely to be more abundant in the balanced sampling under the annotations-in-the-loop mode, as all the mega group cells (e.g., malignant cells) will be mapped to a single group which has a cap on the number of cells sampled.

4. The authors noted that stromal cells exhibited high reconstruction error in the imaging mass cytometry dataset due to "feature-bias" rather than cell rarity. To what extent might severe feature-imbalance skew the Phase I initialization in targeted panels? We suggest adding a note in the Discussion on whether users should preprocess or adjust highly skewed targeted panels before running the algorithm.

We thank the Reviewer for encouraging us to further discuss and provide user guidance for data obtained with targeted panels. We revised Discussion (page 14, lines 587-593) to note that: "Interestingly, in accordance with prior studies[1], there is evidence that in datasets collected with targeted panels, high RE can also reflect and be used to identify feature-imbalance. More specifically, in the imaging mass cytometry data spanning 35 proteins, highly abundant stromal cells exhibited disproportionately elevated RE, in accordance with the relatively low number of stromal proteins included in the panel (**Supplementary Fig. 2D**). Importantly, DR-GEM will still down-sample also high RE abundant cells and hence improve rare cell type detection also in such cases (B cells: 88% vs. 0% with the standard pipeline; **Supplementary Fig. 10E**)."

5. The authors have thoroughly clarified that their method is termed "self-supervised" because it leverages reconstruction error (RE) and weak label consistency as internal supervisory signals. However, with the fast growth of foundation models in single-cell and spatial omics, the term "self-supervised learning" now mostly makes people think of masked modeling or contrastive learning. At its core, DR-GEM's mechanism is actually closer to bootstrapped ensemble learning and distributionally robust optimization. To avoid confusing the readers, we suggest adding a short discussion in the manuscript. This discussion should simply point out the differences between DR-GEM's signal-driven self-supervision and the common "representational self-supervision" used in deep learning models today.

We thank the Reviewer for the comment and agree that the term 'self-supervised' warrants clarification. We revised the Discussion (page 13, lines 560-566) stating that: "It is important to note that the self-supervision applied by DR-GEM is different from representational self-supervision where models learn to perform a task of interest by training to predict parts of the data, as done in masked modeling (e.g., BERT[2], MAE[3]), generative pre-training (GPT)[4], and contrastive learning (e.g., SimCLR)[5]. Given DR-GEM's strong performances when applied with the VAE-based method scVI (**Supplementary Fig. 5C-D**), it may be relevant to enhance foundation models in single cell and potentially other settings where data scarcity or class imbalance are limiting performances."

We also state that DR-GEM "takes principles often used in the context of supervised machine learning such as distributionally robust optimization[6–10], hard-example mining[11,12] and boosting methods[13–15] where a model detects and learns from its own errors." (revised Discussion, page 14, lines 556-559).

REFERENCES

1. Khani, F. & Liang, P. Feature noise induces loss discrepancy across groups. in *International conference on machine learning* 5209–5219 (PMLR, 2020).
2. Devlin, J., Chang, M.-W., Lee, K. & Toutanova, K. BERT: Pre-training of Deep Bidirectional Transformers for Language Understanding. 4171–4186 (2019).
3. K. He *et al.* Masked Autoencoders Are Scalable Vision Learners. in *2022 IEEE/CVF Conference on Computer Vision and Pattern Recognition (CVPR)* 15979–15988 (2022). doi:10.1109/CVPR52688.2022.01553.
4. Brown, T. B. *et al.* Language models are few-shot learners. in *Proceedings of the 34th International Conference on Neural Information Processing Systems* Article 159 (Curran Associates Inc., Vancouver, BC, Canada, 2020).
5. Chen, T., Kornblith, S., Norouzi, M. & Hinton, G. A simple framework for contrastive learning of visual representations. in *Proceedings of the 37th International Conference on Machine Learning* vol. 119 Article 149 (JMLR.org, 2020).
6. Koh, P. W. *et al.* WILDS: A Benchmark of in-the-Wild Distribution Shifts.
7. Duchi, J. & Namkoong, H. Learning Models with Uniform Performance via Distributionally Robust Optimization. Preprint at https://doi.org/10.48550/arXiv.1810.08750 (2020).
8. Levy, D., Carmon, Y., Duchi, J. C. & Sidford, A. Large-Scale Methods for Distributionally Robust Optimization. Preprint at http://arxiv.org/abs/2010.05893 (2020).
9. Sagawa, S., Koh, P. W., Hashimoto, T. B. & Liang, P. Distributionally Robust Neural Networks for Group Shifts: On the Importance of Regularization for Worst-Case Generalization. Preprint at https://doi.org/10.48550/arXiv.1911.08731 (2020).
10. Hamed Rahimian & Sanjay Mehrotra. Frameworks and Results in Distributionally Robust Optimization. *Open J. Math. Optim.* **3**, 1–85 (2022).
11. P. F. Felzenszwalb, R. B. Girshick, D. McAllester, & D. Ramanan. Object Detection with Discriminatively Trained Part-Based Models. *IEEE Trans. Pattern Anal. Mach. Intell.* **32**, 1627–1645 (2010).
12. P. Viola & M. Jones. Rapid object detection using a boosted cascade of simple features. in *Proceedings of the 2001 IEEE Computer Society Conference on Computer Vision and Pattern Recognition. CVPR 2001* vol. 1 I–I (2001).
13. Chen, T. & Guestrin, C. XGBoost: A Scalable Tree Boosting System. *Proceedings of the 22nd ACM SIGKDD International Conference on Knowledge Discovery and Data Mining* 785–794 (2016).
14. Ke, G. *et al.* LightGBM: a highly efficient gradient boosting decision tree. in *Proceedings of the 31st International Conference on Neural Information Processing Systems* 3149–3157 (Curran Associates Inc., Long Beach, California, USA, 2017).
15. Mason, L., Baxter, J., Bartlett, P. & Frean, M. Boosting Algorithms as Gradient Descent. in *Advances in Neural Information Processing Systems* (eds Solla, S., Leen, T. & Müller, K.) vol. 12 (MIT Press, 1999).

Point-by-Point Response to Reviewers

We thank the Reviewers for their time and engagement with the work throughout the review process. We are pleased that the previous revisions addressed all the Reviewers' comments. As no additional comments were raised by the Reviewers, the changes in this round of final revisions address the editorial comments, as detailed in the accompanying checklist and cover letter.

Reviewer #1 (Remarks to the Author):

Author has addressed my concerns. I have no more questions.

Reviewer #2 (Remarks to the Author):

I co-reviewed this manuscript with one of the reviewers who provided the listed reports. This is part of the Nature Communications initiative to facilitate training in peer review and to provide appropriate recognition for Early Career Researchers who co-review manuscripts.

Reviewer #3 (Remarks to the Author):

My questions have been resolved, and I have no further concerns.
